# Core Proteome of the Minimal Cell: Comparative Proteomics of Three Mollicute Species

**DOI:** 10.1371/journal.pone.0021964

**Published:** 2011-07-19

**Authors:** Gleb Y. Fisunov, Dmitry G. Alexeev, Nicolay A. Bazaleev, Valentina G. Ladygina, Maria A. Galyamina, Ilya G. Kondratov, Nadezhda A. Zhukova, Marina V. Serebryakova, Irina A. Demina, Vadim M. Govorun

**Affiliations:** 1 Scientific Research Institute of Physical-Chemical Medicine, Federal Bio-Medical Agency of Russia, Moscow, Russia; 2 Russian Research Centre Kurchatov Institute, Moscow, Russia; 3 Moscow Institute of Physics and Technology, Moscow, Russia; Dana-Farber Cancer Institute, United States of America

## Abstract

*Mollicutes* (mycoplasmas) have been recognized as highly evolved prokaryotes with an extremely small genome size and very limited coding capacity. Thus, they may serve as a model of a ‘minimal cell’: a cell with the lowest possible number of genes yet capable of autonomous self-replication. We present the results of a comparative analysis of proteomes of three mycoplasma species: *A. laidlawii*, *M. gallisepticum,* and *M. mobile*. The core proteome components found in the three mycoplasma species are involved in fundamental cellular processes which are necessary for the free living of cells. They include replication, transcription, translation, and minimal metabolism. The members of the proteome core seem to be tightly interconnected with a number of interactions forming core interactome whether or not additional species-specific proteins are located on the periphery. We also obtained a genome core of the respective organisms and compared it with the proteome core. It was found that the genome core encodes 73 more proteins than the proteome core. Apart of proteins which may not be identified due to technical limitations, there are 24 proteins that seem to not be expressed under the optimal conditions.

## Introduction

A bacterial genome's length varies from 180 Kb in obligate intracellular symbiont *Carsonella rudii* to 13 Mb in soil bacterium *Sorangium cellulosum*. The distribution of genome sizes between bacterial species with sequenced genomes has a bimodal structure with two peaks at 2 Mb and 5 Mb, dividing bacterial genomes into ‘small’ and ‘large’ [1]. Mycoplasmas belong to the group of bacteria with small genomes; their genomes rarely exceed 1 Mb. *Mycoplasma genitalium,* with a 580 Kb chromosome, is considered to be an organism with the smallest genome among autonomously-replicating bacteria. The sequencing of a *M. genitalium* genome led to the emergence of the minimal cell and minimal genome concepts [2].

Since the publication of the first sequenced genomes [3], a number of approaches to the identification of minimal gene sets from autonomously-replicating organisms have been developed. These include comparative genomics, comparative proteomics, experimental identification of essential genes, and theoretical modeling.

The comparative genomics approach was developed first and initially resulted in the identification of 256 genes [3]. However, as the number of sequenced genomes increased, the set of genes conserved in all known genomes dropped to about 50 [4]. It is now clear that, on the one hand, the comparison of distantly related organisms leads to the underestimation of the minimal gene set because of the non-orthologous displacement of essential genes [5]. On the other hand, the identification of the genes conserved between closely-related species results in the overestimation of the minimal gene set because, apart from essential genes, some non-essential genes (but important under the particular conditions) are included in the conserved core.

The next approach to discover the minimal gene set was the identification of essential genes by global transposon mutagenesis or single gene deletions. The first attempt to find essential genes of *M. genitalium* showed that from 265 to 350 out of 517 genes are essential [6]. Enhancing the method gave 382 essential genes [7]. To date, there are a number of publications on the allocation of essential genes; their results are summarized in the Database of Essential Genes (DEG) [8]. For example, a set of essential genes for *Bacillus subtilis* comprises 271 genes [9]. Nonetheless, the estimation of the minimal gene set through gene deletion may face some obstacles. First, some identifications may be false-positive because of mutated genes and their altered products may affect a number of cellular processes like the metabolism and expression of downstream genes. Second, the effect of knocking out all non-essential genes at one time may not be equivalent to the step by step deletion in terms of cell survival. At the same time, some non-essential genes may be conserved among a number of species. According to Fang et al, these genes are involved in cellular homeostasis maintenance and adaptation to stress [10]. The deletion of such genes does not have any effect under laboratory conditions, but they may be crucial for cell survival in long-term periods in the natural environment.

The theoretical modeling of the minimal function set required for cell replication results in the allocation of 151 genes which are necessary and sufficient to sustain a living cell under the most favorable conditions [11]. However, cellular organization on the whole is far from being fully understood. In particular, transcriptome and interactome complexity issues are left out of consideration in this approach.

It is now clear that a minimal gene set greatly depends on selected approaches and organisms and may constitute from 151 [11] to several hundreds [7]. The number of genes in a minimal set tends to rise as the complexity of the selected organism increases. For example, the gene inactivation approach reveals 712 [12] and 614 [13] essential genes found in large bacteria, *E. coli* and *B. subtilis,* and only 382 [7] essential genes are found in *M. genitalium*. Moreover, taking into account the recent data on a large number of untranslated RNAs [14], it is reasonable to consider that the use of only the genomic or proteomic approach is insufficient to discover a minimal gene set. Thus, a proteogenomic approach should be applied in this case. Comparative proteogenomics, proposed by Gupta et al [15], imply the use of genomic techniques to characterize genome and also imply proteomic techniques to increase reliability, correct genome annotation, and identify expressed open reading frames (ORFs).

Callister et al [16] applied the proteogenomic approach to identify a conserved core of 6 bacterial species. However, the selected species lived in different environments, were grown under different conditions and were phylogenetically distant from each other. This resulted in some genes, previously thought to be indispensable, not being included in the core genome.

In this study, we propose to explore the core proteome shared by three mycoplasma species using the proteogenomic approach. These species occupy different ecological niches, but can grow under the same conditions and are phylogenetically close to each other. Thus, this gives us a chance to avoid the non-orthologous displacement of essential genes, and, at the same time, it may help to exclude genes responsible for adaptation to the niche specific conditions.

## Methods

### Strains and Growth Conditions

*Acholeplasma laidlawii PG-8A* and *Mycoplasma gallisepticum S6* were grown in a modified Edward's medium (Tryptose 20 g/L, NaCl 5 g/L, NaOAc 5 g/L, KCl 1.3 g/L, Tris 3 g/L, yeast dialysate 5%, horse serum 6%, glucose 0.5%, pH 7.6) at 37°C for 18 and 24 hours, respectively. The cells were cultured in 500 mL flasks containing 300 mL medium under aerobic conditions. The mycoplasma mobile was grown in an Aluotto medium (Heart infusion broth 25 g/L, yeast extract 5%, horse serum 20%, pH 7.6) [17]. The *Acholeplasma laidlawii PG-8A* strain was provided by Prof. H. Wroblewsky, Université de Rennes. The *Mycoplasma gallisepticum S6* strain was provided by Prof. S.N. Borkhsenius, Institute of Cytology, Russian Academy of Science.

### RNA isolation and real-time PCR

The total RNA was extracted from a cell culture in the mid-logarithm growth phase with the aid of a Trizol LS reagent (Invitrogen). Then, RNA samples were treated with DNAse I (Fermentas) and used for cDNA synthesis with Mu-MLV reverse transcriptase (Fermentas). Real-time PCR using SYBR Green PCR Master Mix (ABI) and an ABI Prism SDS 7000 (ABI) instrument was then performed. Amplicons were designed to cover the middle of each annotated ORF. Primers were designed with PerlPrimer software (Supplementary tables S13 and S14).

### SDS-PAGE

Proteins were solubilized by boiling them in a sample buffer and were then separated by SDS/PAGE gels consisting of 7.5% T or 16.5% T and 2.6% C (% T, gel acrylamide concentration; % C, degree of crosslinking within the polyacrylamide gel), according to the Laemmli method [18]. The gels were fixed and stained with Coomassie G-250.

### Two-dimensional PAGE

Before carrying out, the 2D PAGE cells were treated with a nuclease mix (Amersham Bioscience). The cells were centrifuged and the cell pellet was dissolved in a buffer (10 µl): 8 M urea, 2 M thiourea, 4% CHAPS, 2% (w/v) NP-40, 1% Triton X-100, 2% Ampholytes, pH range 3 to 10, 80 mM DTT. Protein concentration was determined using Quick start Bradford dye reagent (Bio-Rad, USA). Isoelectrofocusing was performed using tube gels (20 cm×1.5 mm) containing carrier ampholytes and applying a voltage gradient in an IEF-chamber Protean II XL cell (Bio-Rad). After IEF, the ejected tube gels were incubated in an equilibration buffer (125 mM TrisHCl, 40% (w/v) glycerol, 3% (w/v) SDS, 65 mM DTT, pH 6.8) for 30 min. The tube gels were placed onto polyacrylamide gels (9–16%) of 1.5-mm thickness, 20×18 cm (Protean II Multi-Cell, Bio-Rad, USA), and fixed using 0.9% (w/v) agarose containing 0.01% (w/v) bromphenol blue. The electrophoresis was carried out for 12–14 hours.

### Gel Staining and Detection of Proteins

The gels were fixed and silver stained as described by Shevchenko et al. [19]. An image analysis was performed using PDQest software (Bio-Rad, USA). All spots were extracted for MALDI- MS analysis.

### Trypsin Digestion and Mass Spectrometry

The protein bands/spots after 1D or 2D-PAGE were subjected to trypsin in-gel hydrolysis, mainly as described in [20]. 1 mm^3^ gel pieces were excised and washed twice with 100 µL of 0.1 M NH4HCO3 (pH 7.5) and 40% acetonitrile mixture for 30 min at 37°C, dehydrated with 100 µL of acetonitrile, and air-dried. Then, they were treated by 3 µL of 12 mg/mL solution of trypsin (Promega) in 50 mM ammonium bicarbonate for 12 h at 37°C. Peptides were extracted with 6 µL of 0.5% trifluoroacetic acid water solution for 30 min.

### MALDI analysis

Aliquots (1 µL) from the sample were mixed on a steel target with 0.3 µL of 2,5-dihydroxybenzoic acid (Aldrich) solution (10 mg mL^–1^ in 30% acetonitrile/0.5% trifluoroacetic acid), and the droplet was left to dry at room temperature. Mass spectra were recorded on the Ultraflex II MALDI-ToF-ToF mass spectrometer (Bruker Daltonik, Germany) equipped with an Nd laser. The [MH]^+^ molecular ions were measured in reflector mode, the accuracy of the mass peak measurement was 0.007%.

Fragment ion spectra were generated by laser-induced dissociation, slightly accelerated by low-energy collision-induced dissociation, using helium as a collision gas. The accuracy of the fragment ions mass peak measurement was 1Da. Correspondence of the found MS/MS fragments to the proteins was performed with the help of Biotools software (Bruker Daltonik, Germany) and a Mascot MS/MS ion search.

Protein identification was carried out by a peptide fingerprint search with the use of Mascot software (Matrix Science Inc., USA) through a NCBI A. laidlawii protein database. One missed cleavage, Met oxidation and Cys-propionamide settings were permitted for peptide search. Protein scores greater than 44 were considered to be significant (p<0.05). Two dimensional PAGE and subsequent MALDI analysis was performed five times for each species with three replicates per sample.

### LC-ESI-MS analysis

LC-ESI-MS analyses (of tryptic peptides after 1D-SDS-PAGE separation of proteins) were performed on a Agilent 1100 series HPLC-ESI/MSD Trap (Agilent Technologies, USA) equipped with a Zorbax 300-SB C18 column and nano-ESI source. The elution conditions consisted of a 0.3 µl/min 20-min ablution by 5% solvent B (80% acetonitrile, 20% water, 0.1% formic acid), a 50-min gradient 5–60%, and then a 20-min gradient 60–90% from solvent B into A (0.1% formic acid water solution). The [MH]^1+-3+^ ions were detected in the 200–2200 m/z range optimized to 800. MS/MS spectra were obtained automatically for all perceptible MS signals. The accuracy of the mass peak measurement was 0.5 Da. Protein identification was carried out by a MS/MS ion search using Mascot software, as mentioned before. Protein scores greater than 31 were considered significant.

Additional validation was performed on ad-hoc software modules taking into account gel bands, physical properties of proteins, and unspecific trypsin digestion for high abundant proteins. The same software allowed us to scan the acquired spectra for incorrectly annotated N-terminal sequences or misannotated proteins. There were three biological replicates and one technical replicate per each biological replicate of each species subjected to LC-MS analysis.

### Hydrophobic proteins extraction

Cells were resuspended in water and exposed to ultrasound (220Hz) on ice four times for 15 sec. Then, the intact cells were centrifuged on 25000 g for 5 min at 4°C. Supernatant was transferred to new tubes and centrifuged on 85000 g for 1 h at 4°C. The pellet was resuspended in water and centrifuged a second time under the same conditions. The pellet was then resuspended in a buffer containing mM NaCl, 10 mM Tris, and 1% Triton X-114 (рН 7.5), and incubated on ice for 30 min. Then, the solution was centrifuged on 16000 g for 30 min, transferred to a new tube, and left for 5 min to allow the phases to separate. The lower phase was used to perform PAGE.

### Comprehensive proteome analysis

**Figure 1:**
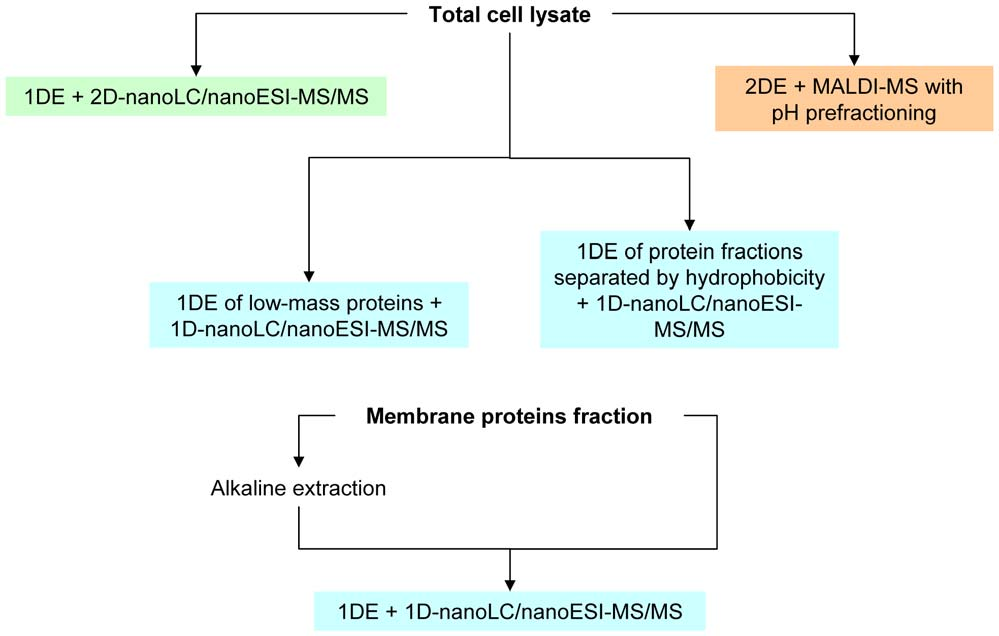
Saturating proteomics workflow chart.

Comprehensive proteome analysis workflow implies a sequential set of protein identification runs. After several runs it reaches a maximum of identified proteins when further runs don't give more proteins. The identified proteins are considered, taking into account technical limitations, to sustain a comprehensive proteomic content of a cell [21]. The overall view on comprehensive proteome analysis workflow is depicted in figure 1. The main issue in comprehensive proteome analysis is a completeness of an acquired proteome. We considered ESI mass spectrometry to be a limiting stage in the way of protein's identification. If there are multiple peptides in a single chromatography peak analyzed by ESI at a time, only major ones will be detected.

To ensure the comprehensiveness of obtained data we used 1D gels zooming. First, the gel gradient was adjusted to a particular proteins mass range. Next, we obtained a full size 1D gel for a selected mass range which gave a significantly higher resolution. We came to the conclusion that gels with 100–120 bands allow to identify all possible proteins for a particular species on our equipment. Further zooming didn't result in the increase in the identified proteins number.

The abundance of high-copied molecular complexes (ribosome, GroEL, pyruvate dehydrogenase, and RNA-polymerase) was estimated by Kuhner et al [22] and was about several hundreds of copies per mycoplasma cell. This means that the dynamic range of protein abundance per mycoplasma cell is about 10^2^.

The sensitivity of 2D electrophoresis coupled with MALDI is about 10^2^. LC-MS allows for the identification of a substantial number of additional proteins, like DNA-polymerase III subunits or components of DNA repair machinery (uvrA, uvrB, uvrC). The sensitivity of LC-MS is 100 fmol and the dynamic range of peptide identification reaches 10^5^. Thus, the dynamic range of our technique sufficiently covers the dynamic range of protein abundance in mycoplasma cells and allows for the identification of several copies of protein per cell.

### *Mycoplasma mobile* proteomic data

The results of the proteomic study for *M. mobile* were taken from work of Jaffe et al [17].

## Results

### Identification of core proteomes

The studied species have small genomes (633 genes in *M. mobile*, 763 genes in *M. gallisepticum,* and 1380 genes in *A. laidlawii*) and, in spite of living in different environments, the species can be cultivated in the same medium. This may allow one to identify the minimal protein set required to sustain a free living cell and to cut off proteins involved in niche adaptation and stress response, which in turn may shed light on the understanding of a vital function set.

Recently, we sequenced the *Acholeplasma laidlawii* genome (Refseq ID: NC_010163) and carried out its proteogenomic annotation. It resulted in the identification of 1380 ORFs. We also resequenced *Mycoplasma gallisepticum S6 strain* genome. There are 762 ORFs (Whole Genome Shotgun ID: AFFR00000000) and there are 633 ORFs in the *Mycoplasma mobile* genome (Refseq ID: NC_006908) according to NCBI.

The proteomic core and genomic core of the minimal free living cell was identified by a comprehensive proteome analysis and genome analysis, respectively, for three mycoplasma species. A comprehensive proteomic analysis of *A. laidlawii* showed an expression of 803 ORFs (58% of annotated ORFs, Supplementary Table S8). The respective proteomic analysis of *M. gallisepticum* allowed us to identify 481 expressed proteins (66% of annotated ORFs, Supplementary Table S9) [23]. The proteome of *M. mobile,* identified by Jaffe et al, consisted of 557 proteins (88% of annotated ORFs).

**Figure 2:**
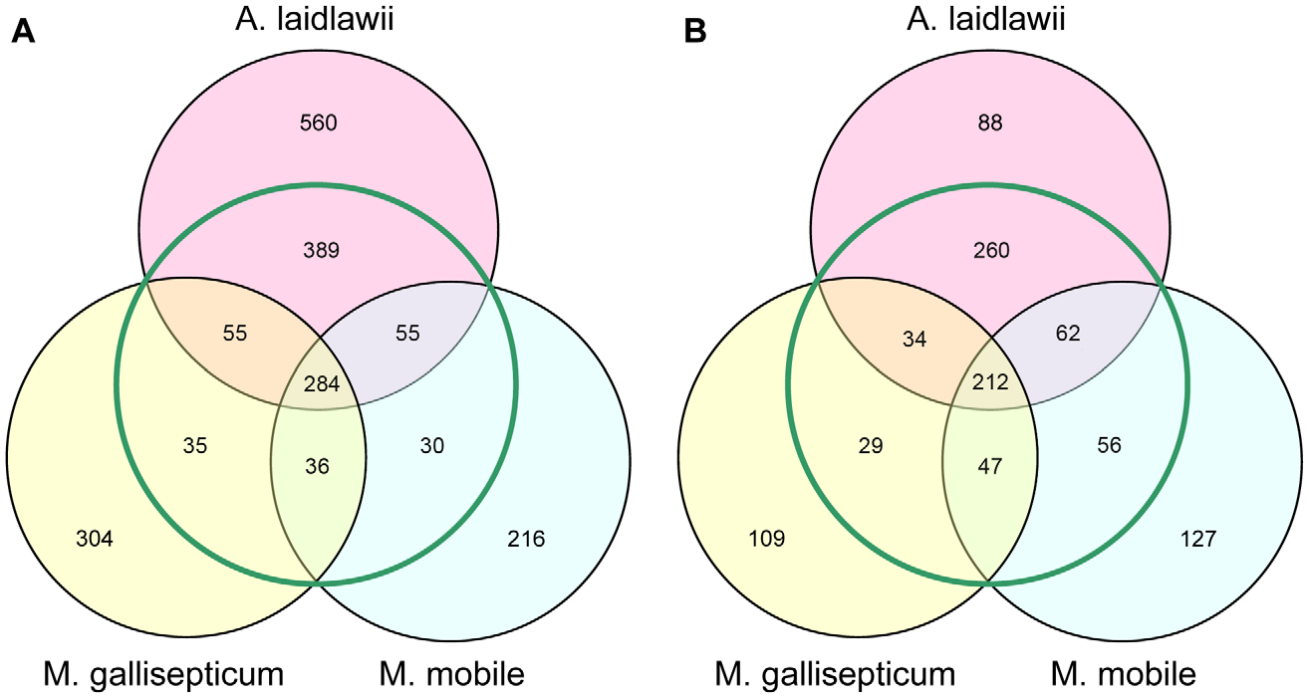
Comparison of genomes and proteomes of three mycoplasma species. Venn diagrams shows the number of common COG's, numbers outside from green circle represent genes and respective proteins without COG. A-diagram for genomes, B-diagram for proteomes.

Clusters of orthologous genes (COGs) assigned to the annotated ORFs were used to compare the genomes and proteomes (Fig. 2). The *A. laidlawii* genome encodes 783 COGs and 560 unique ORFs (without COG assignment); the *M. gallisepticum* genome encodes 409 COGs and 304 unique ORFs; the *M. mobile* genome encodes 404 COGs and 216 unique ORFs. The respective proteomes consist of 567 COGs and 88 unique proteins in *A. laidlawii*, 321 COGs and 109 unique proteins in *M. gallisepticum,* and 374 COGs and 127 unique proteins in *M. mobile*.

The distribution of COGs in genomes and proteomes common for three species and for the pairs of species, for unique COGs, and for unique proteins without a COG were investigated. Genomic and proteomic cores shared by the three species are composed of 284 and 212 COGs, respectively. The proteomic core further underwent a functional analysis in order to study the completeness of essential cellular machineries (replication, transcription, translation, and protein folding) and metabolic pathways.

Comparison of the proteome core with the genome core showed 72 additional ORFs. These may be absent from the proteome core for two reasons: first, they may have extreme physical and chemical properties, like low mass and high hydrophobicity, which makes them hard to be detected by proteomics techniques; second, those proteins may be dispensable under the optimal growth conditions and expressed only in stress. Some of the COGs absent from the proteome core are membrane proteins and small ribosomal proteins and they were likely not detected in our study. There are 37 proteins of this type. Another 35 proteins were predicted not to have extremal properties and should be detected by our approach if presented in a few copies per cell. Their absence in identified proteins means that they are found in less than every cell, even if they are expressed on some level in the whole population. It can be concluded that other proteins are apparently not required for cells to grow under the optimal conditions (Supplementary, Table S1).

The genome and proteome cores were compared with other minimal gene sets obtained by other methods. In particular, the comparison of the proteomic core with the list of essential genes may help to estimate the viability of an organism with such proteomic content. Taking into account the list of essential genes of *M. genitalium* determined by Venter el al [7], only 26 proteins from the proteome core are dispensable (Supplementary Table S10). There are also proteins essential for *M. genitalium,* but absent from the proteome core (111 COGs, Supplementary Table S11). Most of them are small or membrane proteins and may not be identified, or they are absent in one or more of the three mycoplasmas. Thus, the proteomic core meets a good agreement with the list of *M. genitalium* essential genes.

However, it would not be correct to conclude that close relations cause dispensable proteins to be considered as essential ones. This may be true for organisms with larger genomes' and redundant proteins' functionality. In the case of mycoplasmas, which underwent significant genome deterioration and then diverged into different niches, it is not correct. Rather, it indicates the impossibility of building a minimal proteome core based on evolutionary distant species because it leads to the loss of truly essential genes.

### Potential antisense RNAs in core proteome

Remarkably, the proteome core has very few regulation potentials, compared to real cells, even when reduced as mycoplasmas. The role of possible antisense RNA in the regulation of the proteome core was evaluated using data on antisense RNA found in *M. pneumonia* [24]. The intersection of *M. pneumonia* antisense RNAs with the sequences of core proteome genes shows that 35 of its members have antisense transcripts in *M. pneumonia* (Supplementary Table S5).

### Functional characteristics of core proteome

The obtained proteome core contains all or a nearly complete list of the molecular machinery known to be necessary for a free living cell (Supplementary Table S10). It has all the components of basic a DNA replication apparatus except for DNA polymerase I which is absent in genomes of some mycoplasmas. DNA repair systems of the proteome core are represented by a very limited number of proteins. The most complete one is the nucleotide excision repair (NER) system with a total number of 5 proteins, including uvrA, uvrB, uvrC, and two uvrD-like helicases.

The core contains all subunits of RNA polymerase and three transcription factors, which is the maximum number of transcriptional apparatus components in some mycoplasmas. It also has all ribosomal parts that are known to be necessary to its functioning including ribosomal proteins and translation factors, except for IF-1 and RF-2. IF-1 is rather small and was likely to be missed in our proteomics study, while RF-2 is possibly not essential in mycoplasmas as it recognizes stop codon which is replaced by tryptophan codon in mycoplasmas (but not in *Acholeplasma*). In addition, we identified a set of translation coupled proteins like protein chain release factor A, the ribosome recycling factor, and several other proteins.

Moreover, the core proteome contains several molecular chaperones including the DnaK-DnaJ system. The core components found also form a limited number of metabolic pathways, including glycolysis, the non-oxidative part of pentose phosphate pathway, and glycerophospholipid biosynthesis from fatty acids and glycerol. The synthesis of nucleotide triphosphates seems to be a main metabolic capacity of the core.

The most surprising thing in the core proteome is low abundance of cell division proteins compared to the amount of proteins from other essential cellular machineries. There are only two such proteins in the core: FtsH and a Smc-like protein. Although some two of three species have FtsK and FtsZ expressed in the proteome, *M. mobile* doesn't even have corresponding genes in its genome. This may indicate that the cell division mechanism shows greater plasticity compared to other essential systems of the cell.

### Comparative genomics versus comparative proteomics

**Figure 3:**
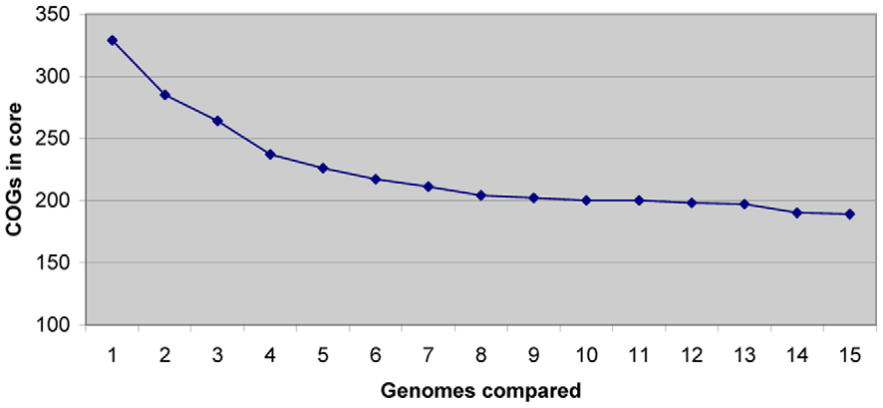
Dependence of genome core size on the number of compared genomes. The plot reaches saturation.

To test comparative proteomics versus comparative genomics we built a genome core for 16 mollicute species and compared the results with the proteome core to estimate the strong and weak points of the two approaches (Supplementary Table S12). The more genomes are taken into comparison the fewer COGs are found in the core (Fig 3). Nevertheless, the curve eventually comes to saturation, i.e. the minimal gene set for the particular phylogenetic group is reached. The common dispensable genes are likely to already be present in this set.

The intersection of the 16 mollicute genomes gives a core of 189 COGs. The proteome core has an amount similar to COGs: 212. However, there are only 156 COGs common for the two cores. The detailed view on the 16 genome cores shows that 15 protein products are supposed to have extreme physico-chemical properties and should be excluded from consideration.

The proteome core, in its turn, has 56 COGs more than the genomic core for 16 mollicutes. More than a half of them are involved in nutrients transport and metabolism. This may indicate that the metabolism is the most variable component of the cell. Metabolic COGs may be treated as adaptive ones because energy sources and available nutrients depend on the particular environment. On the assumption of the proteome core functionality, the main function of the metabolism is to provide the cell with nucleotide triphosphates.

However, the proteome core lacks 14 COGs involved in the efficiency and fidelity of translation and DNA repair. On the one hand, these functions are adaptive, as different environmental conditions require different systems of fidelity maintenance. On the other hand, they may have a complex effect on fidelity and cell survival. These COGs may be essential if all or most of them are deleted at once, being, at the same time, dispensable if deleted individually.

The comparison of the genomic core and our proteome core for the 16 mollicute species shows that there are some conserved metabolic enzymes that do not line up in a complete pathway. For example, there are not any annotated phosphofructokinase and fructose-bisphosphate aldolase enzymes in *M. arthritidis,* nor are there any glyceraldehyde-3-phosphate dehydrogenase enzymes in the *Ureaplasma species*. According to Commichau et al [25], some glycolytic enzymes form complexes with proteins from other cellular systems, like transcription and translation machines. Metabolic enzymes, besides their primary functions, seem to have other activities which they may carry out even in the absence of their metabolic pathways [25].

Thus, comparative genomics help to allocate a core that consists of the basic mechanisms of life but is viable on its own. Comparative proteomics allow us to identify an extended core that approximates the composition of real living cells.

### Transcriptional analysis of ORFs from *A. laidlawii and M. gallisepticum*

We studied the transcriptional activity of ORFs with unknown functions which were not found in proteomic studies of *A. laidlawii* and *M. gallisepticum* by real-time PCR. The respective protein products were supposed not to have extreme physico-chemical functions and should be detected by proteomic techniques. It was found that 151 from 165 studied ORFs of *A. laidlawii* and 101 from 162 studied ORFs of *M. gallisepticum* are expressed (Supplementary Tables S6 and S7). mRNAs of 101 ORFs of *A. laidlawii* and 88 ORFs of *M. gallisepticum* were found at amounts not less than the amount of a β-subunit of RNA polymerase mRNA indicating that a significant number of annotated ORFs in mycoplasmas produce transcripts which do not undergo translation. Rasmussen et al. [14] and Toledo-Arana et al. [26] obtained similar results for *Lysteria monocytogenes* and *B. subtilis*. These studies showed that 98% and 77% of annotated ORFs are expressed in the respective organisms.

### The core interactome

**Figure 4:**
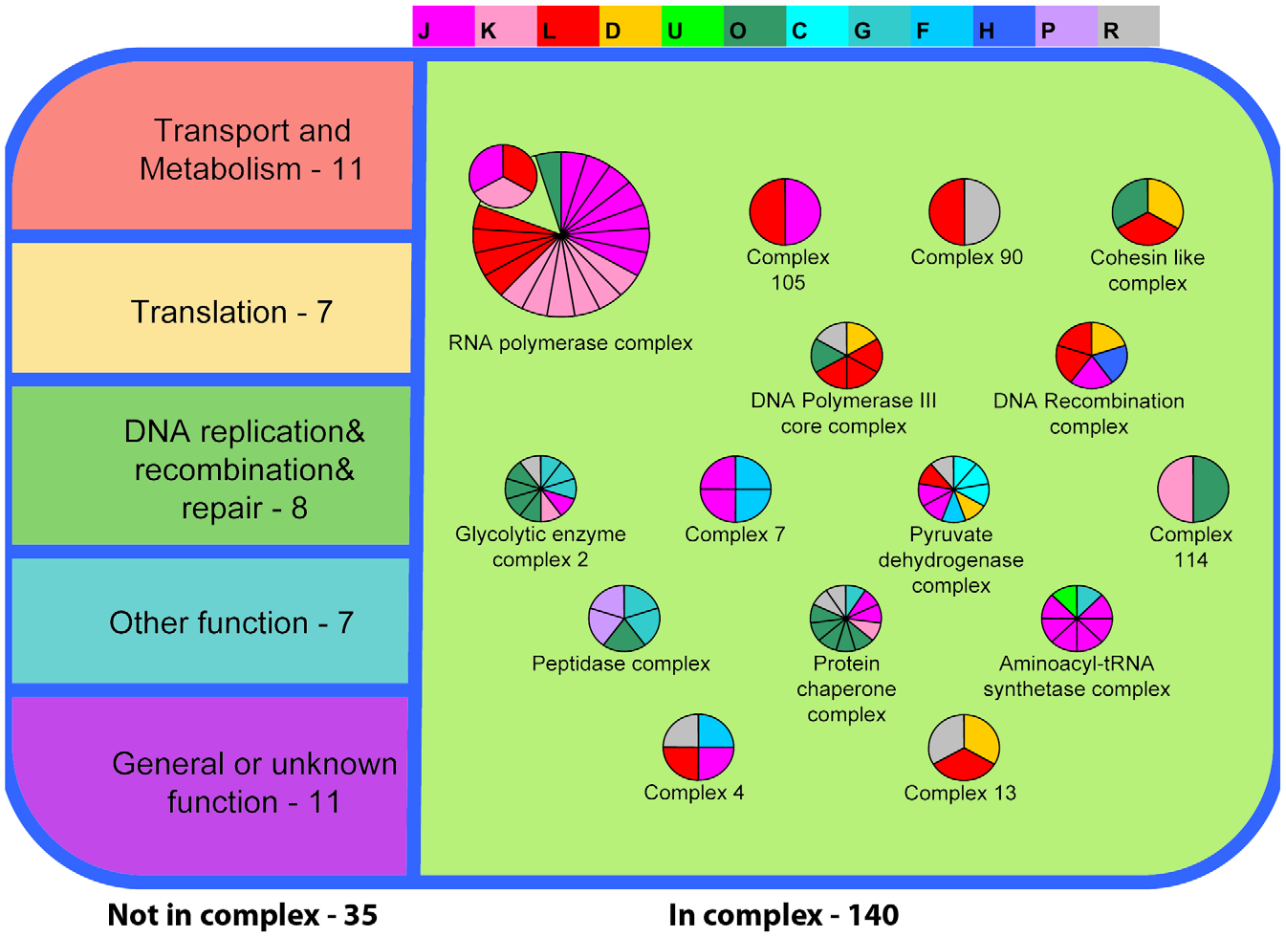
Complexes in the proteome core based on *M. pneumonia* data. Proteins that do not form complexes (35 proteins) are shown on the left. Proteins that are found in complexes (140 proteins) are shown on the right. Color indicates COG functional category (1 sector per protein). Only the most representative complexes are shown. The key for COG functional categories letter code: C Energy production and conversion. D Cell cycle control, cell division, chromosome partitioning. F Nucleotide transport and metabolism. G Carbohydrate transport and metabolism. H Coenzyme transport and metabolism. J Translation, ribosomal structure and biogenesis. K Transcription. L Replication, recombination and repair. O Posttranslational modification, protein turnover, chaperones. P Inorganic ion transport and metabolism. R General function prediction only. U Intracellular trafficking, secretion, and vesicular transport.

We estimated a possible interaction that may be found in the proteome core based on *Mycoplasma pneumonia* interactome data [22]. According to *M. pneumonia* data, most of the COGs in the proteome core (140 COGs) are associated in complexes (Fig 4, Supplementary Table S2). Moreover, 54 COGs participate in more than one complex. Most of the COGs that do not form complexes are ribosomal proteins that are absent in *M. pneumonia*, are transport COGs, or are unknown proteins (Supplementary Table S3).

Among the proteins found only in the genome core, 35 proteins take part in complex formation. Most of them probably were not identified during proteomic studies due to their extreme physico-chemical properties of having low molecular weight, hydrophobicity, and other preventing factors for reliable MS identification. Only 20 proteins which are not likely to be expressed in the proteome core but are found in the genome core actually make complexes (Supplementary Table S4). Most of them are the parts of the defense and DNA repair systems.

**Figure 5:**
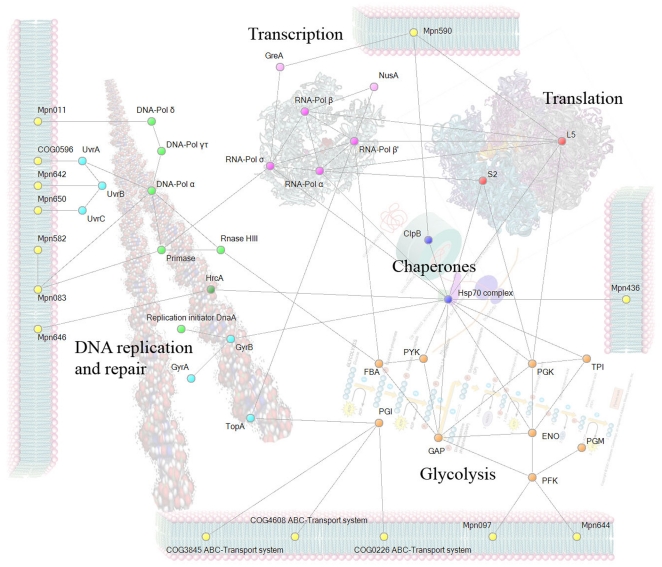
Core proteins interaction chart based on *M. pneumoniae* and *B. subtilis* data. Proteins are divided to groups: DNA handling, transcription, translation, chaperones, glycolysis. Yellow indicates membrane-bound proteins of *M pneumonia* that interact with core proteins. ENO, PGK, S2 and L5 are found in a large number of bacterial species.

Based on *M. pneumonia* and *B. subtilis* protein complexes data, it is possible to estimate the composition of protein complexes in the proteome core. The interactions among selected members of the proteome core are displayed in Figure 5. Proteins of the proteome core form multifunctional complexes. For example, phosphoglycerate kinase, enolase, and two ribosomal proteins (S2 and L5), according to Commichau et al [25], form interactional bridges between glycolysis, a translation apparatus; chaperones hold together glycolysis, translation, transcription, and DNA-binding proteins.

Core proteins, in addition, are bound to membrane proteins (Fig. 5), and respective membrane proteins are specific to *M. pneumonia*. This may indicate that the proteome core is anchored to the membrane through interactions between protein complexes. Thus the main part of the proteome core is bound together into a single complex, starting from DNA and ending on the membrane. Such an arrangement of cellular content may allow for the faster procedure of biological processes, as though it takes place on a conveyor belt.

## Discussion

Undoubtedly, the amount of information provided by comparative proteomics is not less than that of comparative genomics or transcription profiling. The complexity of building a comparison model, which implicates the careful selection of organisms and media, is rewarded by more precise results.

It is now clear that universally conserved genes are too few to sustain a free living cell [4]. Thus life is not invariant to the number of genes in different organisms. More or less, the universal core may be established only for a particular phylogenetic group of organisms. Our core is, more or less, close to the invariant of the mycoplasma clade and may not fit other bacteria.

In a similar study, the core proteome common for 17 bacterial species was found to be composed of 105 proteins, which make up about 74% of the total number of orthologs [16]. In comparison with the mycoplasma proteome core identified by the current study, almost the same proportion of genes (70%) conserved in the genome was found in the proteome core. These two proteome cores, bacterial and mycoplasma, do not match each other completely. This may be explained by the significant genome reduction of mycoplasmas when a number of proteins common in larger bacteria is absent in the mycoplasmas and in the proteome core. At the same time, our core contains 100 more proteins, possibly because we analyzed more close species in contrast with Callister et al [16].

Researchers are paying increased attention to the spatial structure of the cell. For example, Kuhner et al [22], besides a huge amount of interactions covering most cellular proteins, found complexes formed by functionally distant proteins like those involved in translation and metabolism. This led to the conclusion that there are a number of interactions besides classical protein interactions. However, the role of these interactions is poorly understood. The recent data for *B. subtilis* [25] leads to the suggestion that the persistence of glycolytic enzymes genes may have an explanation other than having a metabolic role. They may represent a scaffold for complex formation and modulate the activity of their partners in such complexes.

The possibility of an alternative role of known proteins easily explains the existence of disrupted metabolic pathways, like in *Mycoplasma hominis* [27]. This bacterium has an incomplete glycolytic pathway, the existence of which is even more questionable taking into account that it may fully cover its energy costs through the utilization of arginine. It is possible that those glycolytic proteins are complex-formers rather than metabolic enzymes.

An increasing number of publications show that the cellular organization of bacteria is not less complex than the eukaryotic one [28]. Like eukaryotes, bacteria have a network of intracellular filaments for ordering cellular space, keeping DNA in a particular orientation, and helping it to distribute to daughter cells after replication.

Most, if not all, cellular proteins are participants in multiple complexes, and their partners are also members of other complexes. Thus, cellular proteins make a spatial and functional network, which probably holds all cellular content together with the membrane. Moreover, the periphery of the complex network seems to bind species-specific membrane proteins (Fig. 5) which may be required for fine tuning of complexes. Different species possess different membrane proteins which are bound to different complex components. The changes in membrane protein expression may also affect the spatial organization of the bound complexes in the inner cellular space. This may be a way for cellular reaction on different stimuli in the absence of other regulators, which is common in mycoplasmas.

The composition and structure of protein complexes may maintain genome stability or direct the evolution, as the changes in internal components with a large number of interactions is less likely than changes in periphery which may not affect basic life machinery. Thus, the more a given protein is integrated into the complexes, the less dispensable it is. This is demonstrated by mycoplasmas preservation of the main parts of the proteome core and the changed or lost peripheral parts.

According to this concept, a minimal genome is treated not as a set of essential functions but as a set of essential structures. On the one hand, this structure exposes integrity, consistency, and compactness, and, on the other hand, it has potential to interact with the periphery and to change its configuration and composition.

It is important that the proteome core matches the list of essential genes. However, the comparative proteomic approach may remove issues of non-orthologous gene displacement and false-positive essential genes because it shows the real proteome content of the cell. The issue of importance of persistent genes – genes that are conserved but not essential – also gets a solution. Those that keep expression and participate in complex formation under the optimal conditions are important. Those which are not expressed are dispensable.

According to Jain et al [29], with the increase in the number of interacting partners it becomes harder to remove or alter a single protein in the network. Hence, all main proteins of the most important complexes should tend to conserve their sequence, even under a high level of mutagenesis, and keep their expression level under the different conditions.

Returning to the mycoplasmas, it is reasonable to pay attention to the high divergence of several genes [30] and overall high intraspecie proximity as a result of a limited MMR system [31]. This results in the rapid adaptation of mycoplasmas to the particular environmental conditions by the use of periphery proteins alterations while keeping genes of the core proteins intact. Taking this into account, the successful transplantation of the *Mycoplasma mycoides* genome into *Mycoplasma capricolum* [32] is not surprising because these species have essentially the same protein complex core.

The role of a large amount of non-translated RNAs, described in recent publications, is still unclear. It was found that *B. subtilis* expresses 84 non-coding and 127 antisense RNAs [14]. Similar phenomena were found in small bacteria like *M. pneumonia;* with a genome size six times smaller than the *B. subtilis* genome, it produces 117 non-coding transcripts which comprise 15% of the total number of transcripts [24]. Taking into account a very limited regulatory potential of mycoplasmas (1–2 transcriptional factors per genome), it is reasonable to conclude that non-coding RNAs may play a significant role in gene expression regulation. It is also known that gene regulation does not just control the organism's responses to stimuli but may also be treated as a second genetic code, a key factor which drives genetic differences in more complex organisms [33]. Non-coding RNAs may connect metabolism and gene expression through the riboswitch mechanism [34]. Some core proteome genes also seem to have non-coding antisense transcripts. This may indicate an active usage of antisense RNAs in gene regulation in reduced cells. Hence, the studies of the minimal cell are not possible without taking into account non-coding RNAs.

The obtained systemic comparison of proteomes is in accordance with the latest publications on the composition of the minimal cell and is consistent with the concept of the organization of inner cellular machinery into a number of complexes modulating the member proteins. The common proteome core of the three mycoplasmas seems to support all processes required for a minimal cell to survive in a rich medium and is consistent with the data on essential genes. Moreover, the comparative proteomics approach allows for the separation of proteins required to sustain main cellular functions and proteins involved in adaptation to a particular lifestyle. These facts shows that mycoplasmas are the most suitable object for studying minimal cell content, and the used approach has to include all levels, from genomic to proteomic.

It becomes obvious that the additional layer of comparison on the protein expression level does not just allow us to obtain information that is unavailable on the genomic level, but to introduce a new dimension in the information flow: the spatial organization of the main cellular functions. Thus, studies on the spatial interactions between DNA, RNA, and proteins are inseparable from the studies on minimal cells.

## Supporting Information

Table S1 Proteins with non-extreme properties found in genome core but absent from proteome core.
(DOC)

Table S2 Proteins from core proteome found in *M pneumonia* complexes.
(DOC)

Table S3 Proteins of the core proteome that are not found in complexes.
(DOC)

Table S4 Proteins found in complexes of *M pneumoniae* and found in genome core but not included in proteome core.
(DOC)

Table S5 Antisense transcripts of *Mycoplasma pneumonia* overlapping with genes coding proteins of the proteome core.
(DOC)

Table S6 ORFs of Acholeplasma laidlawii which are transcribed but not translated.
(DOC)

Table S7 ORFs of Mycoplasma gallisepticum which are transcribed but not translated.
(DOC)

Table S8 Peptide coverage of predicted Acholeplasma laidlawii ORFs.
(DOC)

Table S9 Peptide coverage of predicted Mycoplasma gallisepticum ORFs.
(DOC)

Table S10 COG's found in the core genome (the whole table) and the core proteome (highlighted in the “Core Proteome” column).
(DOC)

Table S11 COG's essential in Mycoplasma genitalium but not found in genome core.
(DOC)

Table S12 COG's conserved in genomes of 16 Mycoplasma species.
(XLS)

Table S13 Primers for *Acholeplasma laidlawii* ORFs.
(DOC)

Table S14 Primers for *Mycoplasma gallisepticum* ORFs.
(DOC)
