# Supplementary material for: Core Proteome of the Minimal Cell: Comparative Proteomics of Three Mollicute Species
Source: PLoS One. 2011 Jul 19;6(7):e21964. doi: 10.1371/journal.pone.0021964 (PMC3139596; doi:10.1371/journal.pone.0021964)
Supplement: Table S1 — Proteins with non-extreme properties found in genome core but absent from proteome core. (DOC) [file pone.0021964.s001.doc]

Table S1. Proteins with non-extreme properties found in genome core but absent from proteome core.

| **COG** | **Function** | **Protein** |
| --- | --- | --- |
| COG0009J | Translation, ribosomal structure and biogenesis | Putative translation factor (SUA5) |
| COG0100J | Translation, ribosomal structure and biogenesis | Ribosomal protein S11 |
| COG0130J | Translation, ribosomal structure and biogenesis | Pseudouridine synthase |
| COG0219J | Translation, ribosomal structure and biogenesis | Predicted rRNA methylase (SpoU class) |
| COG0220R | General function prediction only | Predicted S-adenosylmethionine-dependent methyltransferase |
| COG0223J | Translation, ribosomal structure and biogenesis | Methionyl-tRNA formyltransferase |
| COG0225O | Posttranslational modification, protein turnover, chaperones | Peptide methionine sulfoxide reductase |
| COG0229O | Posttranslational modification, protein turnover, chaperones | Conserved domain frequently associated with peptide methionine sulfoxide reductase |
| COG0262H | Coenzyme transport and metabolism | Dihydrofolate reductase |
| COG0266L | Replication, recombination and repair | Formamidopyrimidine-DNA glycosylase |
| COG0286V | Defense mechanisms | Type I restriction-modification system methyltransferase subunit |
| COG0295F | Nucleotide transport and metabolism | Cytidine deaminase |
| COG0319R | General function prediction only | Predicted metal-dependent hydrolase |
| COG0355C | Energy production and conversion | F0F1-type ATP synthase, epsilon subunit (mitochondrial delta subunit) |
| COG0357M | Cell wall/membrane/envelope biogenesis | Predicted S-adenosylmethionine-dependent methyltransferase involved in bacterial cell division |
| COG0389L | Replication, recombination and repair | Nucleotidyltransferase/DNA polymerase involved in DNA repair |
| COG0468L | Replication, recombination and repair | RecA/RadA recombinase |
| COG0482J | Translation, ribosomal structure and biogenesis | Predicted tRNA(5-methylaminomethyl-2-thiouridylate) methyltransferase, contains the PP-loop ATPase domain |
| COG0594J | Translation, ribosomal structure and biogenesis | RNase P protein component |
| COG0610V | Defense mechanisms | Type I site-specific restriction-modification system, R (restriction) subunit and related helicases |
| COG0632L | Replication, recombination and repair | Holliday junction resolvasome, DNA-binding subunit |
| COG0692L | Replication, recombination and repair | Uracil DNA glycosylase |
| COG0712C | Energy production and conversion | F0F1-type ATP synthase, delta subunit (mitochondrial oligomycin sensitivity protein) |
| COG0732V | Defense mechanisms | Restriction endonuclease S subunits |
| COG0736I | Lipid transport and metabolism | Phosphopantetheinyl transferase (holo-ACP synthase) |
| COG0775F | Nucleotide transport and metabolism | Nucleoside phosphorylase |
| COG0816L | Replication, recombination and repair | Predicted endonuclease involved in recombination (possible Holliday junction resolvase in Mycoplasmas and B. subtilis) |
| COG0822C | Energy production and conversion | NifU homolog involved in Fe-S cluster formation |
| COG1159R | General function prediction only | GTPase |
| COG1162R | General function prediction only | Predicted GTPases |
| COG1451R | General function prediction only | Predicted metal-dependent hydrolase |
| COG1481S | Function unknown | Uncharacterized protein conserved in bacteria |
| COG1624S | Function unknown | Uncharacterized conserved protein |
| COG2131F | Nucleotide transport and metabolism | Deoxycytidylate deaminase |
| COG2255L | Replication, recombination and repair | Holliday junction resolvasome, helicase subunit |
| COG3331R | General function prediction only | Penicillin-binding protein-related factor A, putative recombinase |
| COG3611L | Replication, recombination and repair | Replication initiation/membrane attachment protein |
| COG3763S | Function unknown | Uncharacterized protein conserved in bacteria |
