# Supplementary material for: Core Proteome of the Minimal Cell: Comparative Proteomics of Three Mollicute Species
Source: PLoS One. 2011 Jul 19;6(7):e21964. doi: 10.1371/journal.pone.0021964 (PMC3139596; doi:10.1371/journal.pone.0021964)
Supplement: Table S2 — Proteins from core proteome found in M pneumonia complexes. (DOC) [file pone.0021964.s002.doc]

Table S2. Proteins from core proteome found in *M pneumonia* complexes.

| **COMPLEX_NAME** | **COMPLEX_FUNCTION** | **GENE** | **UNIPROT_NAME** |
| --- | --- | --- | --- |
| Aminoacyl-tRNA synthetase complex | Translation, ribosomal structure and biogenesis | Fba | Fructose-bisphosphate aldolase |
| Aminoacyl-tRNA synthetase complex | Translation, ribosomal structure and biogenesis | Ffh | Signal recognition particle protein |
| Aminoacyl-tRNA synthetase complex | Translation, ribosomal structure and biogenesis | PheS | Phenylalanyl-tRNA synthetase ? chain |
| Aminoacyl-tRNA synthetase complex | Translation, ribosomal structure and biogenesis | PheT | Phenylalanyl-tRNA synthetase ? chain |
| Aminoacyl-tRNA synthetase complex | Translation, ribosomal structure and biogenesis | ThrS | Threonyl-tRNA synthetase |
| Aminoacyl-tRNA synthetase complex | Translation, ribosomal structure and biogenesis | TyrS | Tyrosyl-tRNA synthetase |
| ATP synthase complex | Energy production and conversion | AtpF | ATP synthase B chain precursor |
| Cohesin like complex | Cell division and chromosome partitioning | UvrB | UvrABC system protein B |
| Cohesin like complex | Cell division and chromosome partitioning | Lon | ATP-dependent protease La |
| Complex 1 | Function unknown | Mpn195 | Uncharacterized protein MG181 homolog |
| Complex 1 | Function unknown | Apt | Adenine phosphoribosyltransferase |
| Complex 100 | DNA replication, recombination and repair | RuvB | Holliday junction ATP-dependent DNA helicase RuvB |
| Complex 105 | DNA replication, recombination and repair | UvrA | UvrABC system protein A |
| Complex 106 | Function unknown | Fba | Fructose-bisphosphate aldolase |
| Complex 108 | Function unknown | Prs | Ribose-phosphate pyrophosphokinase |
| Complex 108 | Function unknown | ArgS | Arginyl-tRNA synthetase |
| Complex 110 | Function unknown | RpsM | 30S ribosomal protein S13 |
| Complex 110 | Function unknown | TpiA | Triosephosphate isomerase |
| Complex 112 | Function unknown | Mpn521 | Uncharacterized tRNA/rRNA methyltransferase MG346 homolog |
| Complex 114 | Transcription | RpoD | RNA polymerase ? factor RpoD |
| Complex 114 | Transcription | DnaK | Chaperone protein DnaK |
| Complex 116 | Function unknown | Tuf | Elongation factor Tu |
| Complex 13 | Function unknown | AcpS | Holo-[acyl carrier protein] synthase |
| Complex 13 | Function unknown | GidA | tRNA uridine 5-carboxymethylaminomethyl modification enzyme |
| Complex 13 | Function unknown | Mpn563 | Uncharacterized GTP-binding protein MG384 homolog |
| Complex 19 | Function unknown | ThyA | Thymidylate synthase |
| Complex 20 | Function unknown | DnaG | DNA primase |
| Complex 22 | Function unknown | Pth | Peptidyl-tRNA hydrolase |
| Complex 22 | Function unknown | Mpn547 | Uncharacterized protein MG369 homolog |
| Complex 23 | Function unknown | Mpn547 | Uncharacterized protein MG369 homolog |
| Complex 24 | Function unknown | Mpn525 | Uncharacterized protein MG349 homolog |
| Complex 25 | Function unknown | HprK | HPr kinase/phosphorylase |
| Complex 25 | Function unknown | Mpn241 | Uncharacterized protein MG103 homolog |
| Complex 26 | Function unknown | HprK | HPr kinase/phosphorylase |
| Complex 32 | Function unknown | Tig | Trigger factor |
| Complex 33 | Function unknown | RplP | 50S ribosomal protein L16 |
| Complex 33 | Function unknown | UvrB | UvrABC system protein B |
| Complex 33 | Function unknown | Mpn418 | Putative Holliday junction resolvase |
| Complex 36 | Function unknown | TrmE | Probable tRNA modification GTPase TrmE |
| Complex 36 | Function unknown | Mpn009 | Uncharacterized deoxyribonuclease MG009 homolog |
| Complex 36 | Function unknown | ThrS | Threonyl-tRNA synthetase |
| Complex 4 | Function unknown | Gmk | Guanylate kinase |
| Complex 4 | Function unknown | Def | Peptide deformylase |
| Complex 4 | Function unknown | AlaS | Alanyl-tRNA synthetase |
| Complex 46 | Carbohydrate transport and metabolism | Mpn569 | Putative metalloprotease Mpn569 |
| Complex 46 | Carbohydrate transport and metabolism | Eno | Enolase |
| Complex 46 | Carbohydrate transport and metabolism | TpiA | Triosephosphate isomerase |
| Complex 48 | Function unknown | AtpF | ATP synthase B chain precursor |
| Complex 5 | Function unknown | Gmk | Guanylate kinase |
| Complex 5 | Function unknown | Def | Peptide deformylase |
| Complex 51 | Translation, ribosomal structure and biogenesis | RpsZ | 30S ribosomal protein S14 type Z |
| Complex 51 | Translation, ribosomal structure and biogenesis | ThrS | Threonyl-tRNA synthetase |
| Complex 56 | Function unknown | Mpn071 | UPF0011 protein MG056 homolog |
| Complex 61 | Function unknown | Nfo | Probable endonuclease 4 |
| Complex 62 | Function unknown | DeoD | Purine nucleoside phosphorylase DeoD-type |
| Complex 64 | Function unknown | Efp | Elongation factor P |
| Complex 67 | Function unknown | Mpn350 | UPF0078 membrane protein Mpn350 |
| Complex 67 | Function unknown | GreA | Transcription elongation factor GreA |
| Complex 7 | Translation, ribosomal structure and biogenesis | Upp | Uracil phosphoribosyltransferase |
| Complex 7 | Translation, ribosomal structure and biogenesis | DeoA | Thymidine phosphorylase |
| Complex 7 | Translation, ribosomal structure and biogenesis | InfB | Translation initiation factor IF-2 |
| Complex 7 | Translation, ribosomal structure and biogenesis | RbfA | Ribosome-binding factor A |
| Complex 72 | Function unknown | Mpn047 | Uncharacterized protein MG037 homolog |
| Complex 74 | Function unknown | PtsI | Phosphoenolpyruvate-protein phosphotransferase |
| Complex 75 | Function unknown | Mpn461 | Uncharacterized protein MG323 homolog |
| Complex 75 | Function unknown | Cmk | Cytidylate kinase |
| Complex 78 | Function unknown | MsrB | Peptide methionine sulfoxide reductase |
| Complex 83 | Function unknown | SerS | Seryl-tRNA synthetase |
| Complex 85 | Function unknown | GpmI | 2,3-bisphosphoglycerate-independent phosphoglycerate mutase |
| Complex 88 | Function unknown | Prs | Ribose-phosphate pyrophosphokinase |
| Complex 90 | DNA replication, recombination and repair | UvrC | UvrABC system protein C |
| Complex 90 | DNA replication, recombination and repair | Mpn563 | Uncharacterized GTP-binding protein MG384 homolog |
| Complex 91 | Function unknown | SecA | Preprotein translocase subunit SecA |
| Complex 92 | Carbohydrate transport and metabolism | Mpn259 | Uncharacterized protein MG120 homolog |
| Complex 96 | Translation, ribosomal structure and biogenesis | ArgS | Arginyl-tRNA synthetase |
| Complex 98 | Function unknown | EngA | GTP-binding protein EngA |
| Complex 99 | Function unknown | AlaS | Alanyl-tRNA synthetase |
| Cytidine deamination-ribosome complex | Translation, ribosomal structure and biogenesis | Cdd | Cytidine deaminase |
| Cytidine deamination-ribosome complex | Translation, ribosomal structure and biogenesis | RplL | 50S ribosomal protein L7/L12 |
| DNA Polymerase III ? complex | DNA replication, recombination and repair | Mpn007 | Uncharacterized protein MG007 homolog |
| DNA Polymerase III ? complex | DNA replication, recombination and repair | DnaX | DNA polymerase III subunit ?/? |
| DNA Polymerase III core complex | DNA replication, recombination and repair | Gcp | Probable O-sialoglycoprotein endopeptidase |
| DNA Polymerase III core complex | DNA replication, recombination and repair | DnaG | DNA primase |
| DNA Polymerase III core complex | DNA replication, recombination and repair | DnaE | DNA polymerase III subunit ? |
| DNA Polymerase III core complex | DNA replication, recombination and repair | GidA | tRNA uridine 5-carboxymethylaminomethyl modification enzyme |
| DNA Polymerase III core complex | DNA replication, recombination and repair | Mpn563 | Uncharacterized GTP-binding protein MG384 homolog |
| DNA Primase complex | DNA replication, recombination and repair | Mpn014 | Uncharacterized protein MG010 homolog |
| DNA Primase complex | DNA replication, recombination and repair | Mpn014 | Uncharacterized protein MG010 homolog |
| DNA Primase complex | DNA replication, recombination and repair | Ung | Uracil-DNA glycosylase |
| DNA Recombination complex | DNA replication, recombination and repair | ProS | Prolyl-tRNA synthetase |
| DNA Recombination complex | DNA replication, recombination and repair | Mpn424 | UPF0122 protein Mpn424 |
| DNA Recombination complex | DNA replication, recombination and repair | RecA | Protein RecA |
| DNA Recombination complex | DNA replication, recombination and repair | RecA | Protein RecA |
| DnaA complex | DNA replication, recombination and repair | DnaA | Chromosomal replication initiator protein dnaA |
| Dnak-GrpE complex | Posttranslational modification, protein turnover, chaperones | DnaK | Chaperone protein DnaK |
| Glycolytic enzyme complex 1 | Carbohydrate transport and metabolism | Fba | Fructose-bisphosphate aldolase |
| Glycolytic enzyme complex 1 | Carbohydrate transport and metabolism | Pyk | Pyruvate kinase |
| Glycolytic enzyme complex 2 | Carbohydrate transport and metabolism | HrcA | Heat-inducible transcription repressor |
| Glycolytic enzyme complex 2 | Carbohydrate transport and metabolism | GlyQS | Glycyl-tRNA synthetase |
| Glycolytic enzyme complex 2 | Carbohydrate transport and metabolism | Nox | Probable NADH oxidase |
| Glycolytic enzyme complex 2 | Carbohydrate transport and metabolism | Pgk | Phosphoglycerate kinase |
| Glycolytic enzyme complex 2 | Carbohydrate transport and metabolism | DnaK | Chaperone protein DnaK |
| Glycolytic enzyme complex 2 | Carbohydrate transport and metabolism | Eno | Enolase |
| L29-S12 complex | Translation, ribosomal structure and biogenesis | RpmC | 50S ribosomal protein L29 |
| L29-S12 complex | Translation, ribosomal structure and biogenesis | RpsL | 30S ribosomal protein S12 |
| Peptidase complex | Amino acid transport and metabolism | PstB | Phosphate import ATP-binding protein PstB |
| Peptidase complex | Amino acid transport and metabolism | GpmI | 2,3-bisphosphoglycerate-independent phosphoglycerate mutase |
| Peptidase complex | Amino acid transport and metabolism | FtsH | Cell division protease FtsH homolog |
| Phenylalanine-tRNA synthetase complex | Translation, ribosomal structure and biogenesis | PheS | Phenylalanyl-tRNA synthetase ? chain |
| Phenylalanine-tRNA synthetase complex | Translation, ribosomal structure and biogenesis | PheT | Phenylalanyl-tRNA synthetase ? chain |
| Phosphotransfer system complex | Metabolism | Mpn268 | Putative phosphotransferase enzyme IIB component |
| Protein chaperone complex | Posttranslational modification, protein turnover, chaperones | HrcA | Heat-inducible transcription repressor HrcA |
| Protein chaperone complex | Posttranslational modification, protein turnover, chaperones | GlyQS | Glycyl-tRNA synthetase |
| Protein chaperone complex | Posttranslational modification, protein turnover, chaperones | Nox | Probable NADH oxidase |
| Protein chaperone complex | Posttranslational modification, protein turnover, chaperones | Pgk | Phosphoglycerate kinase |
| Protein chaperone complex | Posttranslational modification, protein turnover, chaperones | DnaK | Chaperone protein DnaK |
| Protein chaperone complex | Posttranslational modification, protein turnover, chaperones | RplJ | 50S ribosomal protein L10 |
| Protein chaperone complex | Posttranslational modification, protein turnover, chaperones | Mpn547 | Uncharacterized protein MG369 homolog |
| Pyruvate dehydrogenase complex | Metabolism | Tmk | Thymidylate kinase |
| Pyruvate dehydrogenase complex | Metabolism | TrmE | Probable tRNA modification GTPase TrmE |
| Pyruvate dehydrogenase complex | Metabolism | Mpn009 | Uncharacterized deoxyribonuclease MG009 homolog |
| Pyruvate dehydrogenase complex | Metabolism | PdhB | Pyruvate dehydrogenase E1 component subunit ? |
| Pyruvate dehydrogenase complex | Metabolism | PdhA | Pyruvate dehydrogenase E1 component subunit ? |
| Pyruvate dehydrogenase complex | Metabolism | ThrS | Threonyl-tRNA synthetase |
| Restriction enzyme complex | Defense mechanisms | RpmI | 50S ribosomal protein L35 |
| Restriction enzyme complex | Defense mechanisms | Adk | Adenylate kinase |
| Restriction enzyme complex | Defense mechanisms | DnaB | Replicative DNA helicase |
| Restriction enzyme complex | Defense mechanisms | FtsH | Cell division protease FtsH homolog |
| Ribonucleoside-diphosphate reductase complex | Metabolism | NrdE | Ribonucleoside-diphosphate reductase ? subunit |
| Ribosome complex | Translation, ribosomal structure and biogenesis | Tmk | Thymidylate kinase |
| Ribosome complex | Translation, ribosomal structure and biogenesis | InfC | Translation initiation factor IF-3 |
| Ribosome complex | Translation, ribosomal structure and biogenesis | RplT | 50S ribosomal protein L20 |
| Ribosome complex | Translation, ribosomal structure and biogenesis | NusA | Transcription elongation protein NusA |
| Ribosome complex | Translation, ribosomal structure and biogenesis | InfB | Translation initiation factor IF-2 |
| Ribosome complex | Translation, ribosomal structure and biogenesis | RplC | 50S ribosomal protein L3 |
| Ribosome complex | Translation, ribosomal structure and biogenesis | RplD | 50S ribosomal protein L4 |
| Ribosome complex | Translation, ribosomal structure and biogenesis | RplW | 50S ribosomal protein L23 |
| Ribosome complex | Translation, ribosomal structure and biogenesis | RplB | 50S ribosomal protein L2 |
| Ribosome complex | Translation, ribosomal structure and biogenesis | RpsS | 30S ribosomal protein S19 |
| Ribosome complex | Translation, ribosomal structure and biogenesis | RpsC | 30S ribosomal protein S3 |
| Ribosome complex | Translation, ribosomal structure and biogenesis | RplP | 50S ribosomal protein L16 |
| Ribosome complex | Translation, ribosomal structure and biogenesis | RpsQ | 30S ribosomal protein S17 |
| Ribosome complex | Translation, ribosomal structure and biogenesis | RplX | 50S ribosomal protein L24 |
| Ribosome complex | Translation, ribosomal structure and biogenesis | RplE | 50S ribosomal protein L5 |
| Ribosome complex | Translation, ribosomal structure and biogenesis | RpsZ | 30S ribosomal protein S14 type Z |
| Ribosome complex | Translation, ribosomal structure and biogenesis | RpsH | 30S ribosomal protein S8 |
| Ribosome complex | Translation, ribosomal structure and biogenesis | RplF | 50S ribosomal protein L6 |
| Ribosome complex | Translation, ribosomal structure and biogenesis | RpsE | 30S ribosomal protein S5 |
| Ribosome complex | Translation, ribosomal structure and biogenesis | RpmJ | 50S ribosomal protein L36 |
| Ribosome complex | Translation, ribosomal structure and biogenesis | RpsM | 30S ribosomal protein S13 |
| Ribosome complex | Translation, ribosomal structure and biogenesis | RplQ | 50S ribosomal protein L17 |
| Ribosome complex | Translation, ribosomal structure and biogenesis | RpsB | 30S ribosomal protein S2 |
| Ribosome complex | Translation, ribosomal structure and biogenesis | RplK | 50S ribosomal protein L11 |
| Ribosome complex | Translation, ribosomal structure and biogenesis | RplA | 50S ribosomal protein L1 |
| Ribosome complex | Translation, ribosomal structure and biogenesis | Pth | Peptidyl-tRNA hydrolase |
| Ribosome complex | Translation, ribosomal structure and biogenesis | RpsL | 30S ribosomal protein S12 |
| Ribosome complex | Translation, ribosomal structure and biogenesis | RpsG | 30S ribosomal protein S7 |
| Ribosome complex | Translation, ribosomal structure and biogenesis | RpsF | 30S ribosomal protein S6 |
| Ribosome complex | Translation, ribosomal structure and biogenesis | RpsR | 30S ribosomal protein S18 |
| Ribosome complex | Translation, ribosomal structure and biogenesis | PgsA | CDP-diacylglycerol-glycerol-3-phosphate 3-phosphatidyltransferase |
| Ribosome complex | Translation, ribosomal structure and biogenesis | RpmA | 50S ribosomal protein L27 |
| Ribosome complex | Translation, ribosomal structure and biogenesis | RpsD | 30S ribosomal protein S4 |
| Ribosome complex | Translation, ribosomal structure and biogenesis | EngA | GTP-binding protein EngA |
| Ribosome complex | Translation, ribosomal structure and biogenesis | IleS | Isoleucyl-tRNA synthetase |
| Ribosome complex | Translation, ribosomal structure and biogenesis | RuvB | Holliday junction ATP-dependent DNA helicase RuvB |
| Ribosome complex | Translation, ribosomal structure and biogenesis | RplJ | 50S ribosomal protein L10 |
| Ribosome complex | Translation, ribosomal structure and biogenesis | RplL | 50S ribosomal protein L7/L12 |
| Ribosome complex | Translation, ribosomal structure and biogenesis | RpsT | 30S ribosomal protein S20 |
| Ribosome complex | Translation, ribosomal structure and biogenesis | ThrS | Threonyl-tRNA synthetase |
| Ribosome complex | Translation, ribosomal structure and biogenesis | RpsI | 30S ribosomal protein S9 |
| Ribosome complex | Translation, ribosomal structure and biogenesis | RpsO | 30S ribosomal protein S15 |
| Ribosome complex | Translation, ribosomal structure and biogenesis | Frr | Ribosome recycling factor |
| Ribosome complex | Translation, ribosomal structure and biogenesis | RplS | 50S ribosomal protein L19 |
| Ribosome complex | Translation, ribosomal structure and biogenesis | RpsP | 30S ribosomal protein S16 |
| Ribosome complex | Translation, ribosomal structure and biogenesis | Tuf | Elongation factor Tu |
| RNA polymerase complex | Transcription | Mpn009 | Uncharacterized deoxyribonuclease MG009 homolog |
| RNA polymerase complex | Transcription | Mpn014 | Uncharacterized protein MG010 homolog |
| RNA polymerase complex | Transcription | Mpn067 | Protein MG054 homolog |
| RNA polymerase complex | Transcription | NusA | Transcription elongation protein NusA |
| RNA polymerase complex | Transcription | InfB | Translation initiation factor IF-2 |
| RNA polymerase complex | Transcription | RpmC | 50S ribosomal protein L29 |
| RNA polymerase complex | Transcription | RpsZ | 30S ribosomal protein S14 type Z |
| RNA polymerase complex | Transcription | InfA | Translation initiation factor IF-1 |
| RNA polymerase complex | Transcription | RpoA | DNA-directed RNA polymerase ? chain |
| RNA polymerase complex | Transcription | RplK | 50S ribosomal protein L11 |
| RNA polymerase complex | Transcription | TrpS | Tryptophanyl-tRNA synthetase |
| RNA polymerase complex | Transcription | RpoD | RNA polymerase ? factor RpoD ?-A) |
| RNA polymerase complex | Transcription | GlyQS | Glycyl-tRNA synthetase |
| RNA polymerase complex | Transcription | RecA | Protein RecA |
| RNA polymerase complex | Transcription | RpoC | DNA-directed RNA polymerase ?' chain |
| RNA polymerase complex | Transcription | RpoB | DNA-directed RNA polymerase ? chain |
| RNA polymerase complex | Transcription | RplL | 50S ribosomal protein L7/L12 |
| ScpA-ThyA complex | Metabolism | ThyA | Thymidylate synthase |
| Translation elongation factor complex | Translation, ribosomal structure and biogenesis | Tmk | Thymidylate kinase |
| Translation elongation factor complex | Translation, ribosomal structure and biogenesis | RplB | 50S ribosomal protein L2 |
| Translation elongation factor complex | Translation, ribosomal structure and biogenesis | RpmC | 50S ribosomal protein L29 |
| Translation elongation factor complex | Translation, ribosomal structure and biogenesis | RplK | 50S ribosomal protein L11 |
| Translation elongation factor complex | Translation, ribosomal structure and biogenesis | RpsL | 30S ribosomal protein S12 |
| Translation elongation factor complex | Translation, ribosomal structure and biogenesis | Tsf | Elongation factor Ts |
| Translation elongation factor complex | Translation, ribosomal structure and biogenesis | Tuf | Elongation factor Tu |
