# Supplementary material for: Core Proteome of the Minimal Cell: Comparative Proteomics of Three Mollicute Species
Source: PLoS One. 2011 Jul 19;6(7):e21964. doi: 10.1371/journal.pone.0021964 (PMC3139596; doi:10.1371/journal.pone.0021964)
Supplement: Table S3 — Proteins of the core proteome that are not found in complexes. (DOC) [file pone.0021964.s003.doc]

Table S3. Proteins of the core proteome that are not found in complexes.

| **COG** | **GENE** | **PRODUCT** | **FUNCTIONAL GROUP** |
| --- | --- | --- | --- |
| COG0783P | dps | starvation-inducible DNA-binding protein, ferritin-like protein | DNA Replication&Maintenance |
| COG3331R | recU | recombination protein U | DNA Replication&Maintenance |
| COG2256L | - | Holliday junction DNA helicase-like ATPase | DNA Replication&Maintenance |
| COG0353L | recR | recombination DNA repair protein | DNA Replication&Maintenance |
| COG0272L | ligA | DNA ligase, NAD-dependent | DNA Replication&Maintenance |
| COG0632L | ruvA | holliday junction DNA helicase RuvA | DNA Replication&Maintenance |
| COG1466L | holA | DNA polymerase III, delta subunit | DNA Replication&Maintenance |
| COG0356C | atpB | F-type H+-transporting ATPase a chain | Energy |
| COG0712C | atpH | F-type H+-transporting ATPase delta chain | Energy |
| COG0224C | atpG | F-type H+-transporting ATPase gamma chain | Energy |
| COG0355C | atpC | F-type H+-transporting ATPase epsilon chain | Energy |
| COG0112E | glyA | glycine hydroxymethyltransferase | Metabolism |
| COG0775F | pfs | 5'-methylthioadenosine/S-adenosylhomocysteine nucleosidase | Metabolism |
| COG0282C | ackA | acetate kinase | Metabolism |
| COG0039C | ldh | L-lactate dehydrogenase | Metabolism |
| COG0240C | gpsA | NAD(P)-dependent glycerol-3-phosphate dehydrogenase | Metabolism |
| COG0698G | - | sugar-phosphate isomerase, RpiB/LacA/LacB family | Metabolism |
| COG0036G | rpe | ribulose-phosphate 3-epimerase | Metabolism |
| COG0190H | folD | methylenetetrahydrofolate dehydrogenase (NADP+) | Metabolism |
| COG0220R | - | methyltransferase, S-adenosylmethionine-dependent | Metabolism |
| COG0301H | thiI | thiamine biosynthesis ATP pyrophosphatase | Metabolism |
| COG0221C | ppa | inorganic pyrophosphatase | Metabolism |
| COG0236IQ | acpP | acyl carrier protein | Metabolism |
| COG0416I | plsX | fatty acid/phospholipid synthesis protein | Metabolism |
| COG0510M | - | choline/ethanolamine kinase | Metabolism |
| COG0575I | - | cytidylyltransferase, integral membrane protein | Metabolism |
| COG0528F | pyrH | uridylate kinase | Metabolism |
| COG0634F | hpt | hypoxanthine-guanine phosphoribosyltransferase | Metabolism |
| COG1435F | tdk | thymidine kinase | Metabolism |
| COG0504F | pyrG | cytidine triphosphate (CTP) synthetase | Metabolism |
| COG0006E | pepP | X-Pro aminopeptidase | Posttranslational protein processing |
| COG0552U | ftsY | signal recognition particle GTPase | Posttranslational protein processing |
| COG0201U | secY | preprotein translocase, SecY subunit | Posttranslational protein processing |
| COG0822C | sufU | SUF system FeS cluster assembly protein | Posttranslational protein processing |
| COG0520E | sufS | SUF system FeS cluster assembly protein, cysteine desulfurase | Posttranslational protein processing |
| COG1214O | - | putative glycoprotease, peptidase M22 family | Posttranslational protein processing |
| COG0691O | smpB | SsrA-binding protein | Posttranslational protein processing |
| COG0706U | - | integral membrane protein | Posttranslational protein processing |
| COG0225O | msrA | methionine sulfoxide reductase A | Posttranslational protein processing |
| COG0557K | - | 3'-5' exoribonuclease, RNase R/RNase II family | Transcription |
| COG2740K | - | conserved hypothetical protein | Transcription |
| COG0223J | fmt | methionyl-tRNA formyltransferase | Translation |
| COG0227J | rpmB | large subunit ribosomal protein L28 | Translation |
| COG0051J | rpsJ | small subunit ribosomal protein S10 | Translation |
| COG0091J | rplV | large subunit ribosomal protein L22 | Translation |
| COG0093J | rplN | large subunit ribosomal protein L14 | Translation |
| COG0102J | rplM | large subunit ribosomal protein L13 | Translation |
| COG0200J | rplO | large subunit ribosomal protein L15 | Translation |
| COG0230J | rpmH | large subunit ribosomal protein L34 | Translation |
| COG0254J | rpmE | large subunit ribosomal protein L31 | Translation |
| COG0100J | rpsK | small subunit ribosomal protein S11 | Translation |
| COG0333J | rpmF | large subunit ribosomal protein L32 | Translation |
| COG0261J | rplU | large subunit ribosomal protein L21 | Translation |
| COG0256J | rplR | large subunit ribosomal protein L18 | Translation |
| COG0481M | lepA | translation elongation factor 4 (EF4), GTPase | Translation |
| COG0024J | - | methionine aminopeptidase | Translation |
| COG0030J | ksgA | dimethyladenosine transferase | Translation |
| COG0012J | - | YchF family protein, GTPase | Translation |
| COG0594J | rnpA | ribonuclease P | Translation |
| COG2890J | - | SAM-dependent methyltransferase, HemK family | Translation |
| COG0009J | - | dsRNA binding, YrdC domain protein | Translation |
| COG0130J | truB | tRNA pseudouridine synthase B | Translation |
| COG0336J | trmD | tRNA (guanine-N1-)-methyltransferase | Translation |
| COG0215J | cysS | cysteinyl-tRNA synthetase | Translation |
| COG0495J | leuS | leucyl-tRNA synthetase | Translation |
| COG1190J | lysS | lysyl-tRNA synthetase | Translation |
| COG0525J | valS | valyl-tRNA synthetase | Translation |
| COG0601EP | - | ABC-type transport system, permease component | Transporter |
| COG0580G | - | major intrinsic protein (MIP) superfamily | Transporter |
| COG1970M | mscL | large conductance mechanosensitive channel | Transporter |
| COG0704P | phoU | phosphate uptake regulator | Transporter |
| COG1079R | - | ABC-type transport system, permease component | Transporter |
| COG0168P | trkG | trk system potassium uptake protein TrkG | Transporter |
| COG0718S | - | conserved hypothetical protein | Unknown/General function |
| COG1744R | - | hypothetical surface-anchored protein | Unknown/General function |
| COG1162R | - | RNA-binding GTPase | Unknown/General function |
| COG0218R | - | GTPase, YihA subfamily | Unknown/General function |
| COG3763S | - | conserved hypothetical surface-anchored protein | Unknown/General function |
| COG1624S | - | conserved hypothetical membrane-anchored protein | Unknown/General function |
| COG0217S | - | conserved hypothetical protein | Unknown/General function |
| COG1159R | era | GTP-binding protein | Unknown/General function |
| COG1692S | - | conserved hypothetical protein | Unknown/General function |
| COG1451R | - | conserved hypothetical protein | Unknown/General function |
| COG0357M | gidB | glucose inhibited division protein B | Unknown/General function |
