# Supplementary material for: Core Proteome of the Minimal Cell: Comparative Proteomics of Three Mollicute Species
Source: PLoS One. 2011 Jul 19;6(7):e21964. doi: 10.1371/journal.pone.0021964 (PMC3139596; doi:10.1371/journal.pone.0021964)
Supplement: Table S4 — Proteins found in complexes of M pneumoniae and found in genome core but not included in proteome core. (DOC) [file pone.0021964.s004.doc]

Table S4. Proteins found in complexes of *M pneumoniae* and found in genome core but not included in proteome core.

| **COG** | **Locus** | **Annotation** | **Core/Attachment** | **Name** |
| --- | --- | --- | --- | --- |
| COG0468 | RecA | Protein RecA | core | DNA Recombination complex (1) |
| COG0692 | Ung | Uracil-DNA glycosylase | core | DNA Primase complex (2) |
| COG2255 | RuvB | Holliday junction ATP-dependent DNA helicase RuvB | core | Complex 100 |
| COG0468 | RecA | Protein RecA | attachment | DNA Recombination complex (2) |
| COG0468 | RecA | Protein RecA | attachment | RNA polymerase complex |
| COG0610 | Mpn346 | Uncharacterized protein Mpn346 | core | Complex 7 |
| COG0295 | Cdd | Cytidine deaminase | core | Cytidine deamination ribosome complex |
| COG2255 | RuvB | Holliday junction ATP-dependent DNA helicase RuvB | attachment | Ribosome |
| COG0319 | Mpn569 | Putative metalloprotease Mpn569 | core | Complex 46 |
| COG0262 | FolA | Dihydrofolate reductase | attachment | Pyruvate dehydrogenase complex |
| COG0732 | Mpn638 | Putative type I restriction enzyme specificity protein Mpn638 (S protein) | core | Restriction enzyme complex 44 |
| COG0610 | Mpn347 | Putative type I restriction enzyme MpnORFDP R protein part 1 | core | Restriction enzyme complex 44 |
| COG0286 | Mpn342 | Putative type I restriction enzyme MpnORFDP M protein | core | Restriction enzyme complex 45 |
| COG0732 | Mpn089 | Putative type I restriction enzyme specificity protein Mpn089 (S protein) | core | Restriction enzyme complex 46 |
| COG0732 | Mpn507 | Putative type I restriction enzyme specificity protein Mpn507 (S protein) | core | Restriction enzyme complex 46 |
| COG0286 | Mpn342 | Putative type I restriction enzyme MpnORFDP M protein | core | Restriction enzyme complex 46 |
| COG0732 | Mpn365 | Putative type I restriction enzyme specificity protein Mpn365 (S protein) | core | Restriction enzyme complex 47 |
| COG0610 | Mpn345 | Putative type-1 restriction enzyme MpnORFDP R protein part 2 | core | Restriction enzyme complex 47 |
| COG0732 | Mpn089 | Putative type I restriction enzyme specificity protein Mpn089 (S protein) | core | Restriction enzyme complex 48 |
| COG0732 | Mpn343 | Putative type I restriction enzyme specificity protein Mpn343 (S protein) | core | Restriction enzyme complex 49 |
| COG0732 | Mpn365 | Putative type I restriction enzyme specificity protein Mpn365 (S protein) | core | Restriction enzyme complex 49 |
| COG0732 | Mpn365 | Putative type I restriction enzyme specificity protein Mpn365 (S protein) | core | Restriction enzyme complex 50 |
| COG0732 | Mpn615 | Putative type I restriction enzyme specificity protein Mpn615 (S protein) | core | Restriction enzyme complex 50 |
| COG0732 | Mpn638 | Putative type I restriction enzyme specificity protein Mpn638 (S protein) | attachment | Restriction enzyme complex 51 |
| COG0610 | Mpn347 | Putative type I restriction enzyme MpnORFDP R protein part 1 | core | Restriction enzyme complex 51 |
| COG0816 | AlaS | Alanyl-tRNA synthetase | attachment | Complex 4 |
| COG0736 | AcpS | Holo -[acyl carrier protein] synthase | core | Complex 13 |
| COG3611 | Mpn525 | Uncharacterized protein MG349 homolog | core | Complex 24 |
| COG1481 | Mpn241 | Uncharacterized protein MG103 homolog | core | Complex 25 |
| COG0262 | FolA | Dihydrofolate reductase | attachment | Complex 36 |
| COG0732 | Mpn289 | Putative type I restriction enzyme specificity protein Mpn289 (S protein) | core | Complex 41 |
| COG0389 | Mpn537 | Uncharacterized protein MG360 homolog | core | Complex 78 |
| COG0229 | MsrB | Peptide methionine sulfoxide reductase | core | Complex 78 |
| COG0816 | AlaS | Alanyl-tRNA synthetase | core | Complex 99 |
| COG0219 | Mpn521 | Uncharacterized tRNA/rRNA methyltransferase MG346 homolog | core | Complex 112 |
