# Supplementary material for: Core Proteome of the Minimal Cell: Comparative Proteomics of Three Mollicute Species
Source: PLoS One. 2011 Jul 19;6(7):e21964. doi: 10.1371/journal.pone.0021964 (PMC3139596; doi:10.1371/journal.pone.0021964)
Supplement: Table S5 — Antisense transcripts of Mycoplasma pneumonia overlapping with genes coding proteins of the proteome core. (DOC) [file pone.0021964.s005.doc]

Table S5. Antisense transcripts of *Mycoplasma pneumonia* overlapping with genes coding proteins of the proteome core.

| **GENE NAME** | **GENE** | **COG** | **PRODUCT** |
| --- | --- | --- | --- |
| NEW50 | dnaA | COG0593L | chromosomal replication initiation protein |
| NEW84 | mutB1 | COG0210L | DNA helicase II |
| NEW98 | uvrB | COG0556L | excinuclease ABC subunit B |
| NEW43 | atpD | COG0055C | F0F1 ATP synthase subunit beta |
| NEW21 | ackA | COG0282C | acetate kinase |
| NEW96 | adk | COG0563F | adenylate kinase |
| NEW52 | tsr | COG0191G | fructose-bisphosphate aldolase |
| NEW89 | cpsG | COG1109G | phosphomannomutase/phosphoglucomutase |
| NEW88 | - | COG1488H | putative nicotinate phosphoribosyltransferase |
| NEW13 | pdhA | COG1071C | pyruvate dehydrogenase |
| NEW78 | pyk | COG0469G | pyruvate kinase |
| NEW22 | - | COG0301H | thiamin biosynthesis protein |
| NEW3 | smpB | COG0691O | SsrA-binding protein |
| NEW68 | trxB | COG0492O | thioredoxin reductase |
| NEW69 | vacB | COG0557K | 3'-5' exoribonuclease, RNase R |
| NEW66 | rpsL | COG0048J | 30S ribosomal protein S12 |
| NEW101 | rpsG | COG0049J | 30S ribosomal protein S7 |
| NEW99 | rplA | COG0081J | 50S ribosomal protein L1 |
| NEW64 | rplC | COG0087J | 50S ribosomal protein L3 |
| NEW64 | rplD | COG0088J | 50S ribosomal protein L4 |
| NEW15 | alaS | COG0013J | alanyl-tRNA synthetase |
| NEW101 | fus | COG0480J | elongation factor G |
| NEW20 | ileS | COG0060J | isoleucyl-tRNA synthetase |
| NEW75 | lysS | COG1190J | lysyl-tRNA synthetase |
| NEW96 | map | COG0024J | methionine amino peptidase |
| NEW82 | bcrA | COG1131V | ABC transporter ATP-binding protein |
| NEW31 | - | COG0477GEPR | permease |
| NEW61 | ugpC | COG3839G | sn-glycerol-3-phosphate transport system permease protein |
| NEW51 | msbA | COG1132V | transport ATP-binding protein |
| NEW49 | degV | COG1307S | hypothetical protein |
| NEW45 | - | COG0595R | metallo hydrolase |
| NEW47 | rbgA | COG1161R | ribosomal biogenesis GTPase |
| NEW76 | - | COG0595R | single stranded RNA(DNA) processing enzyme |
