# Supplementary material for: Core Proteome of the Minimal Cell: Comparative Proteomics of Three Mollicute Species
Source: PLoS One. 2011 Jul 19;6(7):e21964. doi: 10.1371/journal.pone.0021964 (PMC3139596; doi:10.1371/journal.pone.0021964)
Supplement: Table S6 — ORFs of Acholeplasma laidlawii which are transcribed but not translated. (DOC) [file pone.0021964.s006.doc]

Table S6. ORFs of Acholeplasma laidlawii which are transcribed but not translated.

| **Locus tag** | **Gene** | **Average delta Ct with gap** |
| --- | --- | --- |
| ACL_0607 | - | Not Expressed |
| ACL_1176 | Glyceraldehyde-3-P dehydrogenase | 0 |
| ACL_0521 | - | -3 |
| ACL_0550 | dnaK | -3,4 |
| ACL_1322 | - | -3,7 |
| ACL_1246 | - | -3,9 |
| ACL_0402 | Enolase | -4,1 |
| ACL_1006 | - | -4,9 |
| ACL_0688 | - | -5 |
| ACL_1007 | - | -5,1 |
| ACL_1005 | - | -5,3 |
| ACL_0427 | - | -5,6 |
| ACL_1073 | - | -5,7 |
| ACL_1012 | - | -6,1 |
| ACL_1229 | - | -6,2 |
| ACL_0135 | - | -7,1 |
| ACL_0052 | - | -7,2 |
| ACL_0348 | - | -7,2 |
| ACL_0053 | - | -7,3 |
| ACL_0144 | - | -7,6 |
| ACL_0525 | - | -7,6 |
| ACL_0891 | - | -7,8 |
| ACL_0120 | - | -7,9 |
| ACL_1342 | - | -7,9 |
| ACL_0173 | - | -8 |
| ACL_1359 | - | -8 |
| ACL_0122 | - | -8,1 |
| ACL_1070 | - | -8,1 |
| ACL_0213 | - | -8,2 |
| ACL_1175 | - | -8,3 |
| ACL_0863 | - | -8,4 |
| ACL_1002 | - | -8,4 |
| ACL_0648 | - | -8,5 |
| ACL_0199 | - | -8,7 |
| ACL_0412 | - | -8,8 |
| ACL_0007 | gyrA | -9,1 |
| ACL_0515 | - | -9,1 |
| ACL_0164 | - | -9,2 |
| ACL_0006 | gyrB | -9,3 |
| ACL_1257 | - | -9,3 |
| ACL_0175 | - | -9,4 |
| ACL_0161 | - | -9,5 |
| ACL_0165 | - | -9,5 |
| ACL_0974 | - | -9,7 |
| ACL_0411 | - | -9,8 |
| ACL_0754 | - | -9,8 |
| ACL_0200 | - | -9,9 |
| ACL_0675 | - | -9,9 |
| ACL_0769 | - | -9,9 |
| ACL_0576 | - | -10 |
| ACL_0234 | - | -10,1 |
| ACL_1355 | - | -10,1 |
| ACL_0948 | - | -10,4 |
| ACL_0247 | polC | -10,5 |
| ACL_0929 | - | -10,5 |
| ACL_0950 | - | -10,5 |
| ACL_1328 | - | -10,5 |
| ACL_0056 | - | -10,6 |
| ACL_0903 | - | -10,6 |
| ACL_0148 | - | -10,7 |
| ACL_0439 | - | -10,7 |
| ACL_0771 | - | -10,7 |
| ACL_1049 | - | -10,7 |
| ACL_0340 | - | -11,1 |
| ACL_0959 | - | -11,1 |
| ACL_1031 | - | -11,1 |
| ACL_1242 | - | -11,1 |
| ACL_0212 | - | -11,2 |
| ACL_0407 | - | -11,3 |
| ACL_0696 | - | -11,3 |
| ACL_0511 | - | -11,6 |
| ACL_0750 | - | -11,7 |
| ACL_1266 | - | -11,7 |
| ACL_0239 | - | -11,8 |
| ACL_0415 | - | -11,8 |
| ACL_0785 | - | -11,8 |
| ACL_0333 | - | -11,9 |
| ACL_1115 | - | -11,9 |
| ACL_1273 | - | -11,9 |
| ACL_0605 | - | -12 |
| ACL_0695 | - | -12 |
| ACL_0131 | - | -12,1 |
| ACL_0649 | - | -12,1 |
| ACL_1263 | - | -12,1 |
| ACL_0236 | dnaE | -12,2 |
| ACL_0713 | - | -12,2 |
| ACL_0697 | - | -12,3 |
| ACL_0179 | - | -12,4 |
| ACL_0612 | - | -12,4 |
| ACL_1080 | - | -12,5 |
| ACL_1057 | - | -12,6 |
| ACL_1265 | - | -12,6 |
| ACL_0171 | rpoB | -12,8 |
| ACL_0207 | - | -12,8 |
| ACL_0438 | - | -12,8 |
| ACL_0902 | - | -12,9 |
| ACL_0231 | - | -13 |
| ACL_0934 | - | -13 |
| ACL_0953 | - | -13 |
| ACL_1082 | - | -13,1 |
| ACL_0833 | - | -13,2 |
| ACL_1261 | - | -13,2 |
| ACL_0599 | - | -13,8 |
| ACL_0917 | - | -13,8 |
| ACL_0446 | - | -13,9 |
| ACL_0570 | - | -14 |
| ACL_0021 | - | -14,2 |
| ACL_0582 | - | -14,2 |
| ACL_0909 | - | -14,2 |
| ACL_0260 | - | -14,4 |
| ACL_1060 | - | -14,4 |
| ACL_0307 | - | -14,5 |
| ACL_0616 | - | -14,5 |
| ACL_0847 | - | -14,7 |
| ACL_0299 | - | -14,9 |
| ACL_1240 | - | -14,9 |
| ACL_0598 | - | -15 |
| ACL_0615 | - | -15 |
| ACL_0620 | - | -15,1 |
| ACL_0617 | - | -15,2 |
| ACL_0716 | - | -15,2 |
| ACL_0728 | - | -15,3 |
| ACL_0922 | - | -15,3 |
| ACL_0924 | - | -15,3 |
| ACL_1196 | - | -15,3 |
| ACL_0577 | - | -15,4 |
| ACL_1024 | - | -15,4 |
| ACL_1030 | - | -15,4 |
| ACL_0409 | - | -15,5 |
| ACL_0608 | - | -15,5 |
| ACL_0583 | - | -15,7 |
| ACL_0611 | - | -15,7 |
| ACL_0762 | - | -15,7 |
| ACL_1211 | - | -15,7 |
| ACL_0579 | - | -15,8 |
| ACL_0851 | - | -15,8 |
| ACL_0618 | - | -15,9 |
| ACL_1256 | - | -15,9 |
| ACL_0609 | - | -16 |
| ACL_1398 | - | -16 |
| ACL_0913 | - | -16,3 |
| ACL_1056 | - | -16,3 |
| ACL_0441 | - | -16,4 |
| ACL_0584 | - | -16,4 |
| ACL_0622 | - | -16,4 |
| ACL_0596 | - | -16,6 |
| ACL_0588 | - | -16,8 |
| ACL_0623 | - | -16,8 |
| ACL_0626 | - | -16,9 |
| ACL_0635 | - | -17,1 |
| ACL_0625 | - | -17,3 |
| ACL_1287 | - | -17,3 |
| ACL_0865 | - | -17,4 |
| ACL_0593 | - | -17,5 |
| ACL_0602 | - | -17,7 |
| ACL_0928 | - | -17,7 |
| ACL_0927 | - | -17,8 |
| ACL_1335 | - | -18,1 |
| ACL_0624 | - | -18,2 |
| ACL_0859 | - | -18,3 |
| ACL_0259 | - | -18,4 |
| ACL_1121 | - | -18,4 |
| ACL_0627 | - | -18,7 |
| ACL_0926 | - | -18,7 |
| ACL_1058 | - | -19,1 |
| ACL_0578 | - | -19,4 |
| ACL_1210 | - | -19,9 |
| ACL_0614 | - | -20 |
| ACL_0604 | - | -20,6 |
| ACL_0597 | - | -21,4 |
| ACL_0587 | - | -22 |
| ACL_0606 | - | -23 |
| ACL_0726 | - | -31 |
