# Supplementary material for: Core Proteome of the Minimal Cell: Comparative Proteomics of Three Mollicute Species
Source: PLoS One. 2011 Jul 19;6(7):e21964. doi: 10.1371/journal.pone.0021964 (PMC3139596; doi:10.1371/journal.pone.0021964)
Supplement: Table S7 — ORFs of Mycoplasma gallisepticum which are transcribed but not translated. (DOC) [file pone.0021964.s007.doc]

Table S7. ORFs of Mycoplasma gallisepticum which are transcribed but not translated.

| **Locus tag** | **Gene** | **Average delta Ct with gap** |
| --- | --- | --- |
| MGA_0994 | - | Not Expressed |
| MGA_0754 | - | Not Expressed |
| MGA_0704 | - | Not Expressed |
| MGA_0657 | - | Not Expressed |
| MGA_0523 | - | Not Expressed |
| MGA_0514 | - | Not Expressed |
| MGA_0508 | - | Not Expressed |
| MGA_0337 | - | Not Expressed |
| MGA_0329 | - | Not Expressed |
| MGA_0111 | - | Not Expressed |
| MGA_0049 | - | Not Expressed |
| MGA_0021 | - | Not Expressed |
| MGA_1315 | - | 5,51 |
| MGA_1322d | - | 5,37 |
| MGA_1263 | - | 5,28 |
| MGA_1161 | - | 5,23 |
| MGA_1321d | - | 5,21 |
| MGA_1265 | - | 4,91 |
| MGA_1260 | - | 4,66 |
| MGA_1325 | - | 4,56 |
| MGA_1267 | - | 4,12 |
| MGA_1188 | - | 3,97 |
| MGA_1268 | - | 3,2 |
| MGA_1271 | - | 2,98 |
| MGA_1164 | - | 2,74 |
| MGA_0279 | dnaK | 2,01 |
| MGA_0907 | - | 1,8 |
| MGA_0549 | - | 1,76 |
| MGA_0956 | - | 1,57 |
| MGA_1011 | - | 1,13 |
| MGA_0588 | - | 0,82 |
| MGA_0830 | - | 0,72 |
| MGA_0816 | - | 0,17 |
| MGA_0221 | - | 0,12 |
| MGA_1186 | Glyceraldehyde-3-P dehydrogenase | 0 |
| MGA_0867 | - | -0,11 |
| MGA_0103 | - | -0,17 |
| MGA_0631 | - | -0,21 |
| MGA_0289 | - | -0,29 |
| MGA_0284 | - | -0,3 |
| MGA_0583 | - | -0,57 |
| MGA_0908 | - | -0,68 |
| MGA_0313 | - | -0,86 |
| MGA_0650 | - | -0,91 |
| MGA_0552 | - | -0,99 |
| MGA_0997 | - | -1,04 |
| MGA_0298 | - | -1,04 |
| MGA_0217 | - | -1,07 |
| MGA_0209 | Enolase | -1,23 |
| MGA_0567 | - | -1,24 |
| MGA_0477 | - | -1,32 |
| MGA_0805 | - | -1,33 |
| MGA_1010 | - | -1,48 |
| MGA_0576 | - | -1,74 |
| MGA_0562 | - | -1,78 |
| MGA_0107 | - | -1,87 |
| MGA_0797 | - | -1,88 |
| MGA_0315 | - | -1,89 |
| MGA_0115 | - | -2,03 |
| MGA_0553 | - | -2,1 |
| MGA_1019 | - | -2,12 |
| MGA_0287 | - | -2,32 |
| MGA_0798 | - | -2,35 |
| MGA_0054 | - | -2,39 |
| MGA_0656 | - | -2,42 |
| MGA_0564 | - | -2,55 |
| MGA_0436 | - | -2,57 |
| MGA_0046 | - | -2,63 |
| MGA_0518 | - | -2,82 |
| MGA_0281 | - | -2,83 |
| MGA_0247 | - | -2,88 |
| MGA_0361 | - | -2,91 |
| MGA_0865 | - | -3 |
| MGA_0132 | - | -3,09 |
| MGA_0837 | - | -3,1 |
| MGA_0312 | - | -3,13 |
| MGA_0474 | - | -3,16 |
| MGA_0019 | - | -3,16 |
| MGA_0099 | - | -3,18 |
| MGA_0365 | - | -3,21 |
| MGA_0323 | - | -3,27 |
| MGA_0344 | - | -3,29 |
| MGA_1027 | - | -3,33 |
| MGA_0052 | - | -3,33 |
| MGA_0558 | - | -3,34 |
| MGA_0310 | - | -3,34 |
| MGA_0817 | - | -3,35 |
| MGA_0584 | - | -3,35 |
| MGA_0844 | - | -3,4 |
| MGA_0485 | - | -3,4 |
| MGA_0680 | - | -3,53 |
| MGA_0346 | - | -3,56 |
| MGA_0280 | - | -3,57 |
| MGA_1029 | - | -3,69 |
| MGA_0765 | - | -3,83 |
| MGA_0123 | - | -3,92 |
| MGA_0082 | - | -3,93 |
| MGA_0487 | - | -4 |
| MGA_1000 | rpoB | -4,17 |
| MGA_0016 | - | -4,18 |
| MGA_0028 | - | -4,24 |
| MGA_0635 | - | -4,38 |
| MGA_0578 | - | -4,58 |
| MGA_0507 | - | -4,99 |
| MGA_0847 | - | -5,01 |
| MGA_0831 | - | -5,01 |
| MGA_1031 | - | -5,29 |
| MGA_0689 | - | -5,4 |
| MGA_0018 | - | -5,48 |
| MGA_0484 | - | -5,81 |
| MGA_0800 | - | -5,88 |
| MGA_0760 | - | -6,03 |
| MGA_0482 | - | -6,1 |
| MGA_0832 | - | -6,13 |
| MGA_0626 | - | -6,24 |
| MGA_0789 | - | -6,38 |
| MGA_0022 | - | -10,67 |
