# Supplementary material for: Core Proteome of the Minimal Cell: Comparative Proteomics of Three Mollicute Species
Source: PLoS One. 2011 Jul 19;6(7):e21964. doi: 10.1371/journal.pone.0021964 (PMC3139596; doi:10.1371/journal.pone.0021964)
Supplement: Table S8 — Peptide coverage of predicted Acholeplasma laidlawii ORFs. (DOC) [file pone.0021964.s008.doc]

Table S8. Peptide coverage of predicted Acholeplasma laidlawii ORFs.

| **Locus Tag** | **Coverage (% of sequence)** | **Peptides count** | **Protein score** |
| --- | --- | --- | --- |
| ACL_0001 | 17,7 | 5 | 282,53 |
| ACL_0005 | 8,6 | 2 | 71,14 |
| ACL_0006 | 36,69 | 18 | 981,72 |
| ACL_0007 | 37,95 | 29 | 1540,37 |
| ACL_0008 | 21,5 | 9 | 543,66 |
| ACL_0009 | 30,5 | 9 | 537,86 |
| ACL_0010 | 25,46 | 5 | 260,87 |
| ACL_0011 | 6,62 | 2 | 60,87 |
| ACL_0012 | 34,78 | 3 | 163,52 |
| ACL_0013 | 41,84 | 8 | 410,03 |
| ACL_0014 | 12,22 | 3 | 132,75 |
| ACL_0015 | 17 | 2 | 46,45 |
| ACL_0016 | 11,07 | 2 | 93,41 |
| ACL_0017 | 54,26 | 4 | 202,25 |
| ACL_0018 | 38,63 | 11 | 700,23 |
| ACL_0019 | 10,47 | 2 | 70,41 |
| ACL_0022 | 31,81 | 10 | 476,02 |
| ACL_0023 | 14,36 | 2 | 80,1 |
| ACL_0027 | 14,51 | 3 | 170,53 |
| ACL_0028 | 51,59 | 30 | 1698,06 |
| ACL_0029 | 44,66 | 8 | 417,15 |
| ACL_0030 | 10,82 | 2 | 71,4 |
| ACL_0031 | 15 | 3 | 86,43 |
| ACL_0032 | 27,51 | 12 | 516,1 |
| ACL_0033 | 36,1 | 7 | 337,45 |
| ACL_0036 | 49,03 | 14 | 1092,64 |
| ACL_0039 | 14 | 7 | 218,26 |
| ACL_0040 | 37,67 | 3 | 91,46 |
| ACL_0041 | 20,99 | 16 | 685,68 |
| ACL_0043 | 40 | 7 | 369,37 |
| ACL_0044 | 33,47 | 7 | 377,95 |
| ACL_0047 | 16,14 | 4 | 211,48 |
| ACL_0048 | 46,53 | 13 | 668,99 |
| ACL_0050 | 48,88 | 11 | 582,35 |
| ACL_0051 | 4,59 | 2 | 120,12 |
| ACL_0054 | 20,56 | 5 | 341,8 |
| ACL_0057 | 52,94 | 4 | 224,46 |
| ACL_0058 | 24,37 | 3 | 184,8 |
| ACL_0061 | 16 | 3 | 171,63 |
| ACL_0062 | 51,61 | 22 | 1156,4 |
| ACL_0063 | 14,1 | 6 | 333,99 |
| ACL_0082 | 29,58 | 6 | 326,55 |
| ACL_0083 | 49,65 | 10 | 685 |
| ACL_0084 | 24,14 | 3 | 130,03 |
| ACL_0085 | 2 | 1 | 22,54 |
| ACL_0086 | 44,12 | 6 | 294,42 |
| ACL_0087 | 48,58 | 7 | 428,57 |
| ACL_0088 | 38,65 | 8 | 411,14 |
| ACL_0089 | 38 | 5 | 294,59 |
| ACL_0090 | 17,39 | 5 | 399,55 |
| ACL_0092 | 57,66 | 6 | 275,32 |
| ACL_0093 | 32,81 | 7 | 366,55 |
| ACL_0094 | 36,96 | 5 | 275,77 |
| ACL_0097 | 45,9 | 7 | 323,41 |
| ACL_0098 | 30,41 | 4 | 308,21 |
| ACL_0099 | 68,89 | 10 | 598,76 |
| ACL_0101 | 42,75 | 5 | 300,4 |
| ACL_0102 | 28,49 | 4 | 252,4 |
| ACL_0103 | 40,52 | 3 | 169,08 |
| ACL_0104 | 55,36 | 8 | 381,58 |
| ACL_0106 | 50,68 | 5 | 257,11 |
| ACL_0108 | 42,86 | 6 | 310,83 |
| ACL_0109 | 16,73 | 2 | 145,65 |
| ACL_0112 | 49,19 | 10 | 476,8 |
| ACL_0113 | 21,54 | 3 | 233,73 |
| ACL_0114 | 40,61 | 10 | 581,82 |
| ACL_0115 | 48,74 | 4 | 227,08 |
| ACL_0116 | 21,52 | 3 | 165,04 |
| ACL_0117 | 24,9 | 13 | 685,88 |
| ACL_0119 | 36,94 | 16 | 873,71 |
| ACL_0121 | 27 | 5 | 159,47 |
| ACL_0123 | 54,58 | 8 | 545,86 |
| ACL_0126 | 30 | 5 | 124,53 |
| ACL_0127 | 26,87 | 4 | 232,31 |
| ACL_0129 | 10,88 | 2 | 62,04 |
| ACL_0130 | 12,41 | 3 | 146,42 |
| ACL_0132 | 45,35 | 22 | 1112,74 |
| ACL_0134 | 13 | 1 | 40,67 |
| ACL_0137 | 62,68 | 9 | 455,8 |
| ACL_0138 | 72,73 | 11 | 519,44 |
| ACL_0139 | 22,49 | 9 | 515,93 |
| ACL_0140 | 15,24 | 6 | 371,1 |
| ACL_0141 | 24 | 5 | 155,35 |
| ACL_0142 | 27,03 | 14 | 576,44 |
| ACL_0143 | 18,66 | 6 | 263,14 |
| ACL_0145 | 9,6 | 5 | 320,86 |
| ACL_0146 | 35,65 | 5 | 329,87 |
| ACL_0150 | 11,51 | 2 | 59,64 |
| ACL_0151 | 36,26 | 6 | 342,51 |
| ACL_0153 | 21,32 | 4 | 271,88 |
| ACL_0154 | 30 | 5 | 161,96 |
| ACL_0155 | 19,68 | 15 | 675,94 |
| ACL_0156 | 15,18 | 5 | 259,54 |
| ACL_0159 | 19,49 | 11 | 701,34 |
| ACL_0162 | 11,28 | 2 | 92,03 |
| ACL_0163 | 16,99 | 4 | 236,36 |
| ACL_0166 | 47,55 | 5 | 237,17 |
| ACL_0167 | 37,39 | 9 | 608,5 |
| ACL_0168 | 55,56 | 9 | 464,29 |
| ACL_0169 | 73,98 | 11 | 550,11 |
| ACL_0170 | 10 | 1 | 48,27 |
| ACL_0171 | 47,21 | 44 | 2064,97 |
| ACL_0172 | 26,4 | 31 | 1356,32 |
| ACL_0177 | 30,97 | 19 | 1071,12 |
| ACL_0180 | 20 | 3 | 81,52 |
| ACL_0182 | 11,62 | 3 | 167,83 |
| ACL_0183 | 32,47 | 12 | 763,5 |
| ACL_0184 | 32,19 | 5 | 237,84 |
| ACL_0185 | 35,46 | 3 | 190,09 |
| ACL_0186 | 53,21 | 8 | 413,6 |
| ACL_0187 | 53,48 | 22 | 1227,84 |
| ACL_0188 | 53,16 | 13 | 950,3 |
| ACL_0189 | 16,67 | 3 | 130,39 |
| ACL_0190 | 30,28 | 5 | 404,14 |
| ACL_0191 | 8,84 | 2 | 106,46 |
| ACL_0192 | 56,38 | 6 | 366,45 |
| ACL_0193 | 27 | 2 | 74,66 |
| ACL_0194 | 50 | 5 | 265,73 |
| ACL_0196 | 30,81 | 15 | 736,95 |
| ACL_0197 | 18,47 | 6 | 294,48 |
| ACL_0198 | 68,75 | 6 | 313,99 |
| ACL_0201 | 25,42 | 5 | 372,5 |
| ACL_0202 | 34,63 | 5 | 231 |
| ACL_0203 | 32,42 | 4 | 180,24 |
| ACL_0204 | 24,5 | 10 | 616,85 |
| ACL_0205 | 56,85 | 9 | 495,92 |
| ACL_0206 | 37,98 | 7 | 262,15 |
| ACL_0208 | 18,06 | 5 | 230,61 |
| ACL_0209 | 41,86 | 9 | 499,04 |
| ACL_0210 | 44,04 | 33 | 1883,32 |
| ACL_0211 | 26,56 | 5 | 299,85 |
| ACL_0214 | 10,6 | 3 | 102,25 |
| ACL_0215 | 36,18 | 10 | 538,63 |
| ACL_0216 | 26,71 | 6 | 348,78 |
| ACL_0217 | 60,24 | 20 | 857,2 |
| ACL_0218 | 27,99 | 8 | 451,68 |
| ACL_0220 | 26,92 | 6 | 291,12 |
| ACL_0221 | 34,15 | 7 | 367,63 |
| ACL_0222 | 22,56 | 6 | 221,92 |
| ACL_0224 | 42,63 | 10 | 615,12 |
| ACL_0226 | 29 | 13 | 341,6 |
| ACL_0227 | 36,23 | 8 | 478,94 |
| ACL_0228 | 12,79 | 3 | 148,26 |
| ACL_0229 | 49,26 | 10 | 506,58 |
| ACL_0230 | 84 | 10 | 327,85 |
| ACL_0232 | 7,88 | 3 | 138,95 |
| ACL_0233 | 11,11 | 4 | 258,3 |
| ACL_0235 | 18,44 | 2 | 87,38 |
| ACL_0236 | 5,6 | 4 | 123,48 |
| ACL_0237 | 50,61 | 15 | 756,3 |
| ACL_0238 | 29,69 | 6 | 359,46 |
| ACL_0240 | 15,86 | 4 | 221,3 |
| ACL_0242 | 27,02 | 16 | 715,64 |
| ACL_0245 | 24,52 | 6 | 220,99 |
| ACL_0247 | 19,35 | 23 | 928,67 |
| ACL_0248 | 9,35 | 2 | 81,49 |
| ACL_0249 | 41,25 | 9 | 544,64 |
| ACL_0250 | 49,87 | 29 | 1458,49 |
| ACL_0251 | 15,38 | 4 | 226,71 |
| ACL_0252 | 20,75 | 5 | 220,25 |
| ACL_0253 | 18,57 | 3 | 123,17 |
| ACL_0256 | 33,57 | 13 | 715,53 |
| ACL_0258 | 21,29 | 4 | 177,09 |
| ACL_0261 | 15,58 | 6 | 254,63 |
| ACL_0262 | 33,81 | 7 | 366,38 |
| ACL_0263 | 12,3 | 4 | 157,03 |
| ACL_0265 | 11,14 | 3 | 169,58 |
| ACL_0266 | 29,07 | 25 | 1177,83 |
| ACL_0267 | 32 | 6 | 150,07 |
| ACL_0268 | 2,97 | 2 | 85,52 |
| ACL_0269 | 29,68 | 14 | 880,77 |
| ACL_0270 | 42,45 | 13 | 880,28 |
| ACL_0271 | 33,78 | 26 | 1228,99 |
| ACL_0275 | 14,42 | 6 | 450,68 |
| ACL_0277 | 9,64 | 2 | 150,84 |
| ACL_0279 | 19,26 | 6 | 237,07 |
| ACL_0280 | 41,68 | 16 | 839,44 |
| ACL_0281 | 23,73 | 7 | 294,52 |
| ACL_0282 | 45,07 | 12 | 692,9 |
| ACL_0283 | 35,71 | 4 | 260,6 |
| ACL_0284 | 64,58 | 10 | 623,59 |
| ACL_0285 | 33,85 | 12 | 656,75 |
| ACL_0286 | 50,71 | 11 | 598,77 |
| ACL_0288 | 16,29 | 2 | 117,81 |
| ACL_0291 | 8 | 2 | 82,74 |
| ACL_0292 | 13,25 | 3 | 105,99 |
| ACL_0295 | 28,27 | 6 | 315,81 |
| ACL_0296 | 37,76 | 12 | 545,58 |
| ACL_0297 | 13,59 | 3 | 92 |
| ACL_0298 | 38,1 | 18 | 745,2 |
| ACL_0302 | 41,87 | 5 | 321,74 |
| ACL_0304 | 46,34 | 8 | 359,73 |
| ACL_0306 | 5,72 | 2 | 45,29 |
| ACL_0309 | 47,14 | 20 | 1051,37 |
| ACL_0310 | 16,6 | 3 | 201,91 |
| ACL_0311 | 18,27 | 4 | 184,74 |
| ACL_0312 | 30,99 | 6 | 296,32 |
| ACL_0314 | 14,86 | 3 | 108,51 |
| ACL_0315 | 35,93 | 4 | 206,75 |
| ACL_0316 | 19,18 | 2 | 86,98 |
| ACL_0317 | 37,43 | 8 | 469,3 |
| ACL_0320 | 62,58 | 26 | 1518,33 |
| ACL_0321 | 53,91 | 6 | 330,19 |
| ACL_0324 | 21 | 11 | 302,93 |
| ACL_0325 | 44,08 | 10 | 463,49 |
| ACL_0326 | 52,08 | 8 | 408,63 |
| ACL_0328 | 28,5 | 7 | 399,46 |
| ACL_0329 | 18,52 | 3 | 120,44 |
| ACL_0330 | 46,47 | 28 | 1494,53 |
| ACL_0331 | 23,01 | 7 | 355,91 |
| ACL_0334 | 24,07 | 4 | 233,44 |
| ACL_0336 | 42,81 | 18 | 894,97 |
| ACL_0337 | 29,02 | 6 | 352,61 |
| ACL_0339 | 32,27 | 12 | 621,79 |
| ACL_0341 | 54,27 | 10 | 540,18 |
| ACL_0342 | 10 | 3 | 92,15 |
| ACL_0343 | 4 | 3 | 73,97 |
| ACL_0344 | 50,17 | 12 | 766,77 |
| ACL_0345 | 21,37 | 7 | 314,45 |
| ACL_0346 | 57,93 | 7 | 337,45 |
| ACL_0347 | 27,99 | 7 | 453,51 |
| ACL_0349 | 29,12 | 10 | 621,66 |
| ACL_0350 | 20 | 12 | 515,61 |
| ACL_0352 | 32,01 | 11 | 518,51 |
| ACL_0353 | 9,77 | 2 | 115,84 |
| ACL_0354 | 45,02 | 26 | 1292,79 |
| ACL_0355 | 35,88 | 8 | 518,61 |
| ACL_0358 | 27 | 5 | 169,81 |
| ACL_0361 | 22 | 5 | 176,61 |
| ACL_0362 | 21,21 | 2 | 117,45 |
| ACL_0365 | 28,4 | 9 | 474,94 |
| ACL_0366 | 31 | 4 | 159,48 |
| ACL_0367 | 7 | 1 | 23,52 |
| ACL_0368 | 13,93 | 13 | 488,21 |
| ACL_0369 | 44,09 | 8 | 393 |
| ACL_0370 | 28,49 | 7 | 382,61 |
| ACL_0371 | 18,79 | 4 | 166,21 |
| ACL_0376 | 35,15 | 6 | 276,51 |
| ACL_0377 | 35,06 | 9 | 433,92 |
| ACL_0378 | 37,28 | 8 | 415,93 |
| ACL_0379 | 33,49 | 15 | 683,77 |
| ACL_0380 | 26,02 | 16 | 711,11 |
| ACL_0381 | 54,96 | 22 | 1192,07 |
| ACL_0382 | 16,43 | 5 | 235,71 |
| ACL_0383 | 7,93 | 2 | 62,17 |
| ACL_0387 | 68,85 | 13 | 812,6 |
| ACL_0389 | 18 | 7 | 194,52 |
| ACL_0390 | 16 | 3 | 80,74 |
| ACL_0391 | 19,82 | 4 | 127,44 |
| ACL_0395 | 12,99 | 4 | 120,36 |
| ACL_0396 | 11,9 | 3 | 110,58 |
| ACL_0397 | 19,67 | 2 | 92,52 |
| ACL_0398 | 27 | 7 | 209,92 |
| ACL_0400 | 49,12 | 15 | 753,12 |
| ACL_0402 | 61,02 | 16 | 1198,05 |
| ACL_0404 | 49 | 7 | 243,34 |
| ACL_0405 | 4,67 | 3 | 121,77 |
| ACL_0406 | 15,44 | 2 | 77,99 |
| ACL_0413 | 27,27 | 5 | 263,93 |
| ACL_0414 | 15 | 4 | 124,03 |
| ACL_0417 | 12,2 | 5 | 248,95 |
| ACL_0420 | 26,11 | 7 | 432,84 |
| ACL_0421 | 28,47 | 2 | 85,52 |
| ACL_0422 | 17,23 | 5 | 254,85 |
| ACL_0426 | 12 | 4 | 171,5 |
| ACL_0430 | 27,03 | 4 | 196,69 |
| ACL_0431 | 31,35 | 5 | 202,23 |
| ACL_0432 | 47,9 | 21 | 941,62 |
| ACL_0433 | 34,39 | 10 | 433,24 |
| ACL_0434 | 23,7 | 5 | 242,45 |
| ACL_0435 | 43,38 | 36 | 2119,06 |
| ACL_0436 | 14,21 | 6 | 285,1 |
| ACL_0440 | 9 | 3 | 81,97 |
| ACL_0447 | 13 | 5 | 136,76 |
| ACL_0448 | 36,97 | 14 | 713,82 |
| ACL_0449 | 33,13 | 10 | 648,41 |
| ACL_0450 | 23 | 6 | 167,6 |
| ACL_0451 | 6 | 1 | 37,62 |
| ACL_0452 | 24,52 | 10 | 530,74 |
| ACL_0455 | 6,45 | 7 | 314,89 |
| ACL_0456 | 5 | 1 | 49,79 |
| ACL_0457 | 25,17 | 2 | 73,31 |
| ACL_0458 | 15,03 | 5 | 280,88 |
| ACL_0460 | 8 | 1 | 34,92 |
| ACL_0461 | 21,29 | 4 | 285,08 |
| ACL_0462 | 8,17 | 2 | 128,5 |
| ACL_0463 | 29,84 | 5 | 300,58 |
| ACL_0464 | 20,15 | 6 | 266,8 |
| ACL_0465 | 14,79 | 2 | 110,39 |
| ACL_0466 | 22 | 5 | 124,57 |
| ACL_0467 | 53,17 | 13 | 658,9 |
| ACL_0469 | 19 | 4 | 177,09 |
| ACL_0471 | 32,2 | 7 | 484,81 |
| ACL_0472 | 12,11 | 3 | 191,62 |
| ACL_0473 | 10,73 | 2 | 124,34 |
| ACL_0474 | 45,35 | 10 | 503,68 |
| ACL_0475 | 65,87 | 6 | 297,9 |
| ACL_0476 | 7,62 | 2 | 107,17 |
| ACL_0478 | 12,07 | 2 | 64,5 |
| ACL_0479 | 28,88 | 14 | 696,39 |
| ACL_0480 | 23 | 7 | 237,92 |
| ACL_0481 | 8,77 | 3 | 107,21 |
| ACL_0482 | 27,21 | 6 | 316,5 |
| ACL_0483 | 39,35 | 6 | 345,56 |
| ACL_0485 | 24,87 | 12 | 628,59 |
| ACL_0487 | 32,16 | 9 | 481,53 |
| ACL_0489 | 12,97 | 2 | 106,63 |
| ACL_0490 | 31 | 4 | 128,99 |
| ACL_0492 | 25,31 | 11 | 598,42 |
| ACL_0493 | 6 | 2 | 51,93 |
| ACL_0496 | 46,31 | 11 | 632,67 |
| ACL_0498 | 21 | 4 | 106,85 |
| ACL_0499 | 14,77 | 2 | 166,16 |
| ACL_0500 | 28,21 | 5 | 327,59 |
| ACL_0501 | 53,86 | 21 | 1345,5 |
| ACL_0502 | 14,15 | 3 | 164,47 |
| ACL_0503 | 32,01 | 6 | 348,55 |
| ACL_0504 | 50 | 7 | 466,78 |
| ACL_0505 | 40,99 | 10 | 557,58 |
| ACL_0506 | 23,42 | 7 | 347,36 |
| ACL_0507 | 22,43 | 5 | 203,06 |
| ACL_0508 | 18,71 | 3 | 124,2 |
| ACL_0509 | 34,59 | 12 | 540,43 |
| ACL_0512 | 6,03 | 2 | 49,96 |
| ACL_0513 | 25 | 9 | 354,9 |
| ACL_0519 | 30,64 | 8 | 420,78 |
| ACL_0520 | 13,98 | 23 | 1023,84 |
| ACL_0522 | 8,58 | 8 | 440,82 |
| ACL_0524 | 45 | 5 | 214,93 |
| ACL_0526 | 34,26 | 7 | 299,92 |
| ACL_0527 | 26,72 | 7 | 282,27 |
| ACL_0528 | 39,27 | 25 | 1064,49 |
| ACL_0529 | 6,94 | 4 | 149,81 |
| ACL_0530 | 16,1 | 4 | 214,05 |
| ACL_0531 | 38,77 | 20 | 981,02 |
| ACL_0532 | 34,13 | 6 | 273,57 |
| ACL_0533 | 29,52 | 7 | 348,71 |
| ACL_0534 | 7,58 | 2 | 94,15 |
| ACL_0535 | 65,81 | 21 | 1284,98 |
| ACL_0536 | 45,19 | 25 | 1313,87 |
| ACL_0537 | 9,42 | 2 | 90,51 |
| ACL_0538 | 38,3 | 9 | 445,06 |
| ACL_0539 | 38,97 | 5 | 320,49 |
| ACL_0541 | 9,08 | 7 | 215,06 |
| ACL_0543 | 32,43 | 5 | 251,44 |
| ACL_0544 | 12 | 2 | 68,58 |
| ACL_0545 | 6 | 3 | 76,04 |
| ACL_0548 | 36,26 | 9 | 501,5 |
| ACL_0549 | 55,26 | 8 | 420,54 |
| ACL_0550 | 60,82 | 31 | 1926,35 |
| ACL_0551 | 19,78 | 5 | 252,1 |
| ACL_0552 | 35,24 | 9 | 428,88 |
| ACL_0553 | 19 | 2 | 65,33 |
| ACL_0555 | 28,43 | 6 | 284,95 |
| ACL_0557 | 26,6 | 3 | 116,4 |
| ACL_0558 | 25,32 | 11 | 548,6 |
| ACL_0559 | 22,93 | 7 | 269,14 |
| ACL_0560 | 23,9 | 13 | 614,34 |
| ACL_0561 | 40,89 | 5 | 269,38 |
| ACL_0562 | 8,12 | 2 | 129,5 |
| ACL_0563 | 19 | 5 | 128,72 |
| ACL_0564 | 13,11 | 3 | 115,69 |
| ACL_0566 | 18 | 2 | 63,18 |
| ACL_0569 | 9,74 | 2 | 139,62 |
| ACL_0573 | 34 | 11 | 298,9 |
| ACL_0574 | 6,19 | 5 | 159,6 |
| ACL_0580 | 35 | 14 | 369,14 |
| ACL_0589 | 21,62 | 8 | 337,92 |
| ACL_0590 | 15,04 | 5 | 235,48 |
| ACL_0591 | 17,57 | 14 | 628,33 |
| ACL_0592 | 17 | 5 | 145,64 |
| ACL_0600 | 9 | 2 | 49,12 |
| ACL_0603 | 14,08 | 6 | 358,95 |
| ACL_0610 | 16 | 4 | 96,62 |
| ACL_0621 | 5 | 1 | 23,39 |
| ACL_0633 | 27,64 | 19 | 954,48 |
| ACL_0634 | 6,86 | 2 | 86,26 |
| ACL_0636 | 14 | 2 | 48,8 |
| ACL_0637 | 17,31 | 4 | 170,63 |
| ACL_0639 | 8,02 | 2 | 81,27 |
| ACL_0645 | 5 | 2 | 68,82 |
| ACL_0646 | 33 | 5 | 235,7 |
| ACL_0653 | 12,45 | 2 | 115,84 |
| ACL_0655 | 28,34 | 10 | 520,17 |
| ACL_0656 | 25,13 | 12 | 494,24 |
| ACL_0657 | 22,5 | 10 | 481,81 |
| ACL_0658 | 25,82 | 8 | 353,26 |
| ACL_0660 | 7,94 | 4 | 267,32 |
| ACL_0661 | 6,44 | 3 | 236,49 |
| ACL_0662 | 28,49 | 8 | 512,37 |
| ACL_0663 | 16 | 2 | 62,11 |
| ACL_0665 | 11,11 | 8 | 322,61 |
| ACL_0666 | 21,19 | 2 | 104,23 |
| ACL_0667 | 9,62 | 3 | 160,06 |
| ACL_0668 | 38,83 | 9 | 500,27 |
| ACL_0671 | 15 | 3 | 113,59 |
| ACL_0672 | 37,69 | 10 | 632,01 |
| ACL_0676 | 26,93 | 14 | 643,95 |
| ACL_0678 | 27 | 7 | 228,12 |
| ACL_0679 | 12 | 3 | 71,47 |
| ACL_0680 | 27,46 | 7 | 437 |
| ACL_0682 | 10,99 | 2 | 76,05 |
| ACL_0683 | 47,22 | 15 | 969,21 |
| ACL_0685 | 11,11 | 2 | 63,7 |
| ACL_0686 | 31 | 7 | 225,06 |
| ACL_0687 | 52,94 | 6 | 351,48 |
| ACL_0689 | 38 | 6 | 161,46 |
| ACL_0691 | 13,89 | 3 | 107,28 |
| ACL_0692 | 58,73 | 19 | 1035,82 |
| ACL_0698 | 44,49 | 10 | 379,76 |
| ACL_0699 | 27,93 | 6 | 256,94 |
| ACL_0700 | 23,79 | 6 | 305,01 |
| ACL_0701 | 28,81 | 5 | 214,3 |
| ACL_0702 | 32,48 | 24 | 1103,22 |
| ACL_0703 | 35,19 | 2 | 141,03 |
| ACL_0704 | 38 | 6 | 203,69 |
| ACL_0705 | 49,45 | 13 | 882,28 |
| ACL_0706 | 37,27 | 9 | 494,21 |
| ACL_0707 | 30,77 | 7 | 363,01 |
| ACL_0710 | 30,69 | 5 | 196,81 |
| ACL_0711 | 11,42 | 5 | 272,36 |
| ACL_0712 | 5,06 | 2 | 88,85 |
| ACL_0714 | 13,43 | 4 | 178,9 |
| ACL_0717 | 8,42 | 2 | 57,22 |
| ACL_0718 | 3 | 1 | 26,37 |
| ACL_0720 | 4,17 | 2 | 83,97 |
| ACL_0725 | 45,3 | 15 | 801,12 |
| ACL_0727 | 16,1 | 3 | 173,46 |
| ACL_0730 | 8,62 | 7 | 272,13 |
| ACL_0731 | 35,93 | 6 | 280,83 |
| ACL_0733 | 25,75 | 12 | 644,27 |
| ACL_0736 | 40,86 | 7 | 300,16 |
| ACL_0737 | 18 | 10 | 327,03 |
| ACL_0738 | 32,6 | 6 | 382,82 |
| ACL_0740 | 72,67 | 14 | 909,3 |
| ACL_0741 | 34,93 | 3 | 152,93 |
| ACL_0743 | 9,22 | 2 | 121,64 |
| ACL_0747 | 23,04 | 4 | 243,72 |
| ACL_0751 | 17,29 | 5 | 221,17 |
| ACL_0754 | 13,08 | 5 | 280,52 |
| ACL_0759 | 8,02 | 2 | 70,71 |
| ACL_0760 | 13,22 | 3 | 114,12 |
| ACL_0763 | 47,11 | 11 | 495,22 |
| ACL_0764 | 11 | 4 | 125,16 |
| ACL_0765 | 29,04 | 6 | 358,01 |
| ACL_0767 | 32,85 | 9 | 442,95 |
| ACL_0770 | 7,83 | 3 | 102,02 |
| ACL_0777 | 5,88 | 2 | 80,89 |
| ACL_0780 | 18,57 | 7 | 370,79 |
| ACL_0786 | 28 | 4 | 125,58 |
| ACL_0787 | 22,87 | 5 | 304,07 |
| ACL_0788 | 24,09 | 5 | 184,1 |
| ACL_0789 | 7,5 | 2 | 44 |
| ACL_0790 | 10,95 | 4 | 139,84 |
| ACL_0791 | 16 | 5 | 260 |
| ACL_0792 | 10 | 3 | 78,87 |
| ACL_0793 | 26,46 | 6 | 381,47 |
| ACL_0794 | 15,32 | 2 | 171,85 |
| ACL_0797 | 23 | 6 | 187,24 |
| ACL_0798 | 20 | 4 | 111,44 |
| ACL_0799 | 6 | 1 | 39,28 |
| ACL_0801 | 40,48 | 5 | 229,45 |
| ACL_0802 | 7,59 | 2 | 64,32 |
| ACL_0803 | 10,71 | 6 | 381,26 |
| ACL_0808 | 44,34 | 22 | 1236,12 |
| ACL_0809 | 25,84 | 2 | 89,07 |
| ACL_0810 | 34,58 | 4 | 231,37 |
| ACL_0811 | 7,74 | 2 | 69,1 |
| ACL_0814 | 10,57 | 6 | 272,41 |
| ACL_0815 | 9,82 | 2 | 71,57 |
| ACL_0822 | 32,73 | 4 | 179,92 |
| ACL_0823 | 35,1 | 16 | 706,56 |
| ACL_0824 | 30 | 10 | 543,74 |
| ACL_0825 | 21,98 | 4 | 172,8 |
| ACL_0826 | 18 | 4 | 145,81 |
| ACL_0828 | 18,23 | 10 | 473,51 |
| ACL_0829 | 18,82 | 3 | 159,54 |
| ACL_0830 | 27 | 9 | 291,79 |
| ACL_0831 | 71,1 | 8 | 306,35 |
| ACL_0832 | 48,75 | 21 | 982,45 |
| ACL_0833 | 12,83 | 2 | 54,06 |
| ACL_0835 | 30,65 | 3 | 163,05 |
| ACL_0836 | 9 | 1 | 64,66 |
| ACL_0837 | 55,37 | 11 | 762,16 |
| ACL_0838 | 24,64 | 6 | 277,33 |
| ACL_0839 | 33,22 | 7 | 374,77 |
| ACL_0841 | 37,3 | 4 | 246,62 |
| ACL_0843 | 34,64 | 4 | 274,61 |
| ACL_0848 | 34,7 | 10 | 472,46 |
| ACL_0849 | 15,81 | 6 | 243,88 |
| ACL_0850 | 9 | 2 | 63,95 |
| ACL_0853 | 11,35 | 5 | 183,99 |
| ACL_0857 | 9,33 | 2 | 102,68 |
| ACL_0862 | 11,95 | 2 | 132,36 |
| ACL_0864 | 68,18 | 3 | 146,93 |
| ACL_0866 | 20,87 | 18 | 781,29 |
| ACL_0867 | 24,57 | 6 | 399,38 |
| ACL_0868 | 37,84 | 4 | 167,5 |
| ACL_0869 | 28 | 4 | 159,59 |
| ACL_0871 | 30,06 | 7 | 382,63 |
| ACL_0872 | 56,67 | 5 | 290,28 |
| ACL_0873 | 39,88 | 10 | 539,24 |
| ACL_0874 | 29 | 8 | 569,97 |
| ACL_0875 | 40,21 | 20 | 1211,85 |
| ACL_0876 | 39,64 | 8 | 381,41 |
| ACL_0877 | 30,25 | 5 | 254,3 |
| ACL_0878 | 18,38 | 3 | 184,99 |
| ACL_0883 | 19,26 | 12 | 379,87 |
| ACL_0884 | 28,96 | 16 | 663,73 |
| ACL_0885 | 24 | 3 | 74,63 |
| ACL_0887 | 14,55 | 2 | 117,98 |
| ACL_0888 | 8,93 | 3 | 188,34 |
| ACL_0889 | 19 | 7 | 173,93 |
| ACL_0890 | 29 | 7 | 323,61 |
| ACL_0893 | 28,19 | 7 | 424,91 |
| ACL_0894 | 19,05 | 5 | 301,02 |
| ACL_0896 | 13 | 1 | 23,02 |
| ACL_0897 | 26,92 | 2 | 108,8 |
| ACL_0899 | 7,89 | 2 | 66,71 |
| ACL_0900 | 17,58 | 3 | 156,42 |
| ACL_0901 | 45,57 | 8 | 300,08 |
| ACL_0906 | 37,25 | 20 | 903,61 |
| ACL_0908 | 26 | 7 | 200,71 |
| ACL_0910 | 18,81 | 4 | 208,25 |
| ACL_0914 | 42,4 | 9 | 492,67 |
| ACL_0915 | 7,85 | 2 | 89,76 |
| ACL_0916 | 12 | 3 | 113,45 |
| ACL_0918 | 6,64 | 2 | 97,32 |
| ACL_0930 | 9 | 3 | 75,01 |
| ACL_0931 | 26,05 | 9 | 347,66 |
| ACL_0932 | 20 | 3 | 75,98 |
| ACL_0943 | 13,11 | 3 | 82,83 |
| ACL_0944 | 35,32 | 15 | 838,52 |
| ACL_0945 | 25,74 | 11 | 455,34 |
| ACL_0946 | 41,89 | 14 | 622,84 |
| ACL_0949 | 17,69 | 2 | 107,86 |
| ACL_0951 | 18,93 | 5 | 250,52 |
| ACL_0955 | 15,04 | 3 | 210,44 |
| ACL_0956 | 34 | 5 | 128,5 |
| ACL_0960 | 13,13 | 2 | 136,02 |
| ACL_0961 | 31,11 | 14 | 675,19 |
| ACL_0962 | 10,69 | 4 | 222,59 |
| ACL_0963 | 37,67 | 10 | 560,47 |
| ACL_0964 | 7,5 | 3 | 143,19 |
| ACL_0965 | 24,47 | 8 | 431,73 |
| ACL_0967 | 15,85 | 8 | 452,2 |
| ACL_0968 | 62 | 9 | 236,03 |
| ACL_0970 | 27,93 | 5 | 245,12 |
| ACL_0971 | 11,27 | 2 | 115,49 |
| ACL_0972 | 48,8 | 16 | 923,36 |
| ACL_0973 | 35,98 | 17 | 910,06 |
| ACL_0975 | 19,54 | 5 | 281,65 |
| ACL_0976 | 42,29 | 8 | 444,84 |
| ACL_0977 | 15,95 | 2 | 113,09 |
| ACL_0978 | 12,91 | 7 | 290,42 |
| ACL_0982 | 57,39 | 20 | 1238,07 |
| ACL_0983 | 17,02 | 3 | 195,23 |
| ACL_0984 | 26,75 | 11 | 533,64 |
| ACL_0986 | 20 | 3 | 131,19 |
| ACL_0991 | 48,05 | 16 | 960,3 |
| ACL_0992 | 41,87 | 14 | 765,41 |
| ACL_0993 | 27,76 | 12 | 740,07 |
| ACL_0994 | 31,18 | 14 | 675,66 |
| ACL_0995 | 29,37 | 3 | 129,52 |
| ACL_0996 | 22,6 | 2 | 83,69 |
| ACL_0998 | 37,38 | 10 | 498,62 |
| ACL_0999 | 29,61 | 7 | 356,63 |
| ACL_1000 | 11,6 | 4 | 182,76 |
| ACL_1001 | 25,93 | 5 | 287,94 |
| ACL_1003 | 37,45 | 8 | 477,23 |
| ACL_1004 | 8,46 | 2 | 62,64 |
| ACL_1009 | 25,78 | 11 | 453,95 |
| ACL_1011 | 15,18 | 2 | 99,51 |
| ACL_1013 | 10,53 | 2 | 117,42 |
| ACL_1014 | 6,87 | 2 | 82,26 |
| ACL_1015 | 6,87 | 4 | 134,32 |
| ACL_1016 | 18,18 | 4 | 110,01 |
| ACL_1017 | 39,66 | 8 | 518,8 |
| ACL_1019 | 60,16 | 11 | 640,23 |
| ACL_1020 | 21,86 | 3 | 183,84 |
| ACL_1021 | 28,89 | 14 | 770,35 |
| ACL_1023 | 29,51 | 14 | 562,76 |
| ACL_1025 | 69,82 | 11 | 619,61 |
| ACL_1028 | 23,25 | 7 | 295,47 |
| ACL_1029 | 7 | 1 | 30,88 |
| ACL_1032 | 9 | 3 | 105,86 |
| ACL_1033 | 12,9 | 2 | 79,39 |
| ACL_1034 | 27,92 | 12 | 704,35 |
| ACL_1035 | 12,13 | 3 | 175,43 |
| ACL_1036 | 29,13 | 10 | 574,86 |
| ACL_1037 | 16,74 | 4 | 206,41 |
| ACL_1039 | 34,41 | 10 | 472,34 |
| ACL_1042 | 20 | 5 | 157,59 |
| ACL_1043 | 31,65 | 9 | 534,79 |
| ACL_1044 | 18,7 | 6 | 282,31 |
| ACL_1045 | 31,45 | 7 | 489,29 |
| ACL_1046 | 13,18 | 3 | 183,82 |
| ACL_1047 | 11,16 | 2 | 74,37 |
| ACL_1050 | 22,16 | 5 | 320,91 |
| ACL_1051 | 19 | 2 | 89,13 |
| ACL_1052 | 14,2 | 3 | 106,86 |
| ACL_1054 | 5 | 2 | 49,7 |
| ACL_1061 | 7 | 6 | 196,83 |
| ACL_1063 | 16 | 8 | 202,87 |
| ACL_1069 | 5,47 | 3 | 111,88 |
| ACL_1071 | 17 | 5 | 223,26 |
| ACL_1072 | 15,94 | 4 | 237,57 |
| ACL_1075 | 23,74 | 8 | 608,58 |
| ACL_1084 | 14 | 3 | 121,29 |
| ACL_1086 | 14 | 1 | 80,8 |
| ACL_1088 | 34 | 9 | 265,65 |
| ACL_1089 | 5,94 | 3 | 149,65 |
| ACL_1090 | 55,56 | 17 | 1077,63 |
| ACL_1091 | 41,77 | 13 | 632,44 |
| ACL_1092 | 46,86 | 12 | 727,43 |
| ACL_1093 | 8,54 | 3 | 86,93 |
| ACL_1094 | 4 | 1 | 27,46 |
| ACL_1095 | 30,71 | 8 | 440,84 |
| ACL_1096 | 14,16 | 4 | 203,5 |
| ACL_1099 | 14 | 2 | 64,18 |
| ACL_1100 | 4 | 1 | 73,61 |
| ACL_1102 | 13,4 | 3 | 123,91 |
| ACL_1103 | 44,02 | 20 | 893,88 |
| ACL_1106 | 22,97 | 6 | 333,54 |
| ACL_1109 | 24 | 7 | 198,82 |
| ACL_1110 | 51 | 10 | 270,49 |
| ACL_1117 | 5,73 | 2 | 91,39 |
| ACL_1118 | 7 | 14 | 363,92 |
| ACL_1119 | 1,24 | 3 | 71,45 |
| ACL_1123 | 14 | 3 | 97,03 |
| ACL_1124 | 53,67 | 26 | 1260,16 |
| ACL_1125 | 13,85 | 4 | 191,42 |
| ACL_1127 | 11,94 | 5 | 253,1 |
| ACL_1130 | 16,89 | 2 | 120,01 |
| ACL_1132 | 28 | 13 | 385,41 |
| ACL_1139 | 14,49 | 2 | 86,29 |
| ACL_1141 | 51,21 | 11 | 430,03 |
| ACL_1148 | 13,4 | 4 | 265,43 |
| ACL_1150 | 38 | 5 | 170,01 |
| ACL_1151 | 23,87 | 6 | 320,62 |
| ACL_1154 | 47,83 | 6 | 455,08 |
| ACL_1155 | 8,79 | 2 | 87,83 |
| ACL_1156 | 66,22 | 19 | 1179,97 |
| ACL_1157 | 33,63 | 9 | 453,95 |
| ACL_1158 | 52,74 | 5 | 388,11 |
| ACL_1160 | 7,84 | 2 | 102,74 |
| ACL_1161 | 19,91 | 7 | 281,44 |
| ACL_1162 | 14 | 2 | 75,25 |
| ACL_1167 | 29,52 | 4 | 184,96 |
| ACL_1168 | 30,13 | 10 | 515,18 |
| ACL_1169 | 57,12 | 25 | 1173,76 |
| ACL_1170 | 42,33 | 9 | 451,13 |
| ACL_1171 | 24,04 | 2 | 75,75 |
| ACL_1173 | 9,11 | 4 | 275,41 |
| ACL_1174 | 21,57 | 6 | 288,19 |
| ACL_1176 | 46,59 | 8 | 625,01 |
| ACL_1177 | 30,27 | 5 | 174,79 |
| ACL_1180 | 35,95 | 4 | 253,36 |
| ACL_1181 | 38,67 | 19 | 1013,04 |
| ACL_1182 | 26,65 | 5 | 358,01 |
| ACL_1184 | 13,93 | 3 | 133,85 |
| ACL_1185 | 35,85 | 10 | 607,63 |
| ACL_1186 | 39,09 | 8 | 575,19 |
| ACL_1187 | 59,17 | 5 | 235,49 |
| ACL_1188 | 29,63 | 5 | 206,61 |
| ACL_1193 | 16,02 | 2 | 120,22 |
| ACL_1194 | 18,75 | 2 | 102,53 |
| ACL_1195 | 19,55 | 11 | 471,45 |
| ACL_1198 | 35,44 | 9 | 408,79 |
| ACL_1199 | 25,97 | 3 | 129,22 |
| ACL_1200 | 39,39 | 7 | 342,06 |
| ACL_1201 | 21,3 | 4 | 229,27 |
| ACL_1202 | 11,69 | 3 | 183,13 |
| ACL_1203 | 20,66 | 5 | 240,07 |
| ACL_1204 | 23,91 | 5 | 227,58 |
| ACL_1206 | 60,24 | 11 | 560,65 |
| ACL_1208 | 17,03 | 3 | 120,02 |
| ACL_1209 | 63,16 | 17 | 1083,73 |
| ACL_1212 | 9,81 | 3 | 196,88 |
| ACL_1213 | 38,59 | 13 | 564,41 |
| ACL_1214 | 43,92 | 5 | 162,94 |
| ACL_1215 | 28,25 | 8 | 388,44 |
| ACL_1216 | 41,72 | 9 | 462,56 |
| ACL_1217 | 48,24 | 8 | 461,52 |
| ACL_1219 | 24,88 | 8 | 415,83 |
| ACL_1220 | 55,28 | 14 | 805,43 |
| ACL_1221 | 12,59 | 3 | 165,06 |
| ACL_1222 | 21,32 | 4 | 188,67 |
| ACL_1223 | 59,52 | 31 | 2004,24 |
| ACL_1225 | 19,79 | 12 | 544,62 |
| ACL_1228 | 24,62 | 5 | 341,19 |
| ACL_1231 | 8 | 4 | 122,94 |
| ACL_1236 | 17,12 | 4 | 173,84 |
| ACL_1238 | 4,58 | 3 | 146,4 |
| ACL_1241 | 11,83 | 6 | 280,39 |
| ACL_1243 | 27 | 4 | 111,43 |
| ACL_1247 | 52,8 | 19 | 1067,69 |
| ACL_1248 | 43,02 | 3 | 160,94 |
| ACL_1250 | 18 | 3 | 104,28 |
| ACL_1252 | 10 | 5 | 126,9 |
| ACL_1254 | 20 | 13 | 369,7 |
| ACL_1258 | 8,76 | 4 | 168,73 |
| ACL_1259 | 40 | 3 | 107,8 |
| ACL_1262 | 20,94 | 5 | 333,36 |
| ACL_1271 | 24 | 9 | 227,63 |
| ACL_1272 | 26,98 | 11 | 552,59 |
| ACL_1274 | 28,62 | 13 | 507,11 |
| ACL_1278 | 34,29 | 7 | 438,02 |
| ACL_1279 | 22 | 3 | 130,21 |
| ACL_1281 | 27,56 | 17 | 774,75 |
| ACL_1282 | 25 | 11 | 495,31 |
| ACL_1283 | 7,86 | 2 | 60,36 |
| ACL_1286 | 16,89 | 4 | 202,95 |
| ACL_1289 | 30,08 | 7 | 476,93 |
| ACL_1290 | 42,71 | 24 | 1128,09 |
| ACL_1293 | 41,08 | 25 | 1151,01 |
| ACL_1294 | 34,3 | 11 | 483,2 |
| ACL_1296 | 15,38 | 3 | 119,17 |
| ACL_1297 | 9,64 | 3 | 130,18 |
| ACL_1298 | 20,83 | 3 | 143,81 |
| ACL_1299 | 28,05 | 3 | 169,32 |
| ACL_1300 | 24 | 3 | 111,53 |
| ACL_1303 | 16,88 | 4 | 227,67 |
| ACL_1304 | 28 | 7 | 210,73 |
| ACL_1305 | 19,67 | 4 | 157,42 |
| ACL_1306 | 25 | 5 | 129,29 |
| ACL_1309 | 25,98 | 8 | 434,91 |
| ACL_1310 | 50,18 | 21 | 1126,57 |
| ACL_1311 | 56,27 | 12 | 816,08 |
| ACL_1312 | 49,31 | 16 | 1243,53 |
| ACL_1314 | 16 | 2 | 50,36 |
| ACL_1316 | 29,07 | 6 | 324,64 |
| ACL_1317 | 34,62 | 12 | 646,47 |
| ACL_1318 | 7,04 | 2 | 72,75 |
| ACL_1319 | 11 | 2 | 94,92 |
| ACL_1320 | 15,38 | 6 | 324,79 |
| ACL_1321 | 52,27 | 13 | 670,32 |
| ACL_1332 | 14 | 4 | 138,03 |
| ACL_1333 | 8 | 2 | 54,81 |
| ACL_1334 | 15,9 | 3 | 165,95 |
| ACL_1336 | 30,23 | 7 | 388,72 |
| ACL_1339 | 52,21 | 34 | 1543,72 |
| ACL_1340 | 28,32 | 7 | 351,41 |
| ACL_1343 | 39,2 | 8 | 471,16 |
| ACL_1344 | 28,74 | 7 | 400,69 |
| ACL_1345 | 29,02 | 6 | 411,86 |
| ACL_1347 | 53,04 | 39 | 1868,94 |
| ACL_1348 | 42,79 | 20 | 1084,87 |
| ACL_1350 | 13,31 | 11 | 553,11 |
| ACL_1351 | 10,38 | 4 | 166,18 |
| ACL_1352 | 47,05 | 23 | 1117,41 |
| ACL_1353 | 3,99 | 2 | 100,14 |
| ACL_1354 | 26,59 | 7 | 390,28 |
| ACL_1356 | 30,96 | 4 | 215,79 |
| ACL_1357 | 27,7 | 3 | 125,85 |
| ACL_1358 | 31,91 | 11 | 580,79 |
| ACL_1361 | 24 | 3 | 98,4 |
| ACL_1362 | 15 | 2 | 49,87 |
| ACL_1366 | 13,47 | 5 | 277,23 |
| ACL_1370 | 40 | 11 | 311,3 |
| ACL_1373 | 45,75 | 9 | 460,22 |
| ACL_1374 | 26,8 | 5 | 357,38 |
| ACL_1375 | 60,71 | 7 | 286,17 |
| ACL_1376 | 17,93 | 3 | 247,28 |
| ACL_1377 | 32 | 10 | 299,46 |
| ACL_1378 | 17,86 | 4 | 255,03 |
| ACL_1379 | 37,24 | 7 | 402,78 |
| ACL_1380 | 5 | 2 | 87,91 |
| ACL_1383 | 25,79 | 4 | 208,02 |
| ACL_1384 | 7,88 | 2 | 95,4 |
| ACL_1385 | 40,78 | 5 | 256,1 |
| ACL_1386 | 29,49 | 15 | 1071,02 |
| ACL_1388 | 31,72 | 13 | 649,31 |
| ACL_1390 | 45,61 | 6 | 298,82 |
| ACL_1392 | 18,31 | 4 | 141,38 |
| ACL_1394 | 12,3 | 2 | 95,53 |
| ACL_1395 | 60,98 | 15 | 990,03 |
| ACL_1396 | 27,24 | 10 | 432,69 |
| ACL_1399 | 23,16 | 4 | 202,54 |
| ACL_1400 | 47 | 5 | 182,47 |
| ACL_1401 | 26 | 2 | 72,6 |
| ACL_1402 | 31,03 | 15 | 719,98 |
| ACL_1403 | 8,04 | 2 | 172 |
| ACL_1405 | 39,62 | 8 | 502,76 |
| ACL_1406 | 14,65 | 3 | 152,83 |
| ACL_1407 | 49,75 | 10 | 506,41 |
| ACL_1408 | 21,94 | 5 | 269,32 |
| ACL_1409 | 50,27 | 23 | 1365,29 |
| ACL_1410 | 47,01 | 18 | 1172,92 |
| ACL_1411 | 14 | 3 | 93,59 |
| ACL_1412 | 9,77 | 2 | 59,48 |
| ACL_1413 | 40,9 | 18 | 989,07 |
| ACL_1415 | 18,18 | 4 | 160,77 |
| ACL_1416 | 19,39 | 7 | 342,28 |
| ACL_1418 | 28,76 | 4 | 221,42 |
| ACL_1419 | 10,11 | 2 | 124,73 |
| ACL_1420 | 19,38 | 2 | 98,21 |
| ACL_1423 | 8,08 | 2 | 59,76 |
| ACL_1427 | 24,41 | 9 | 416,02 |
| ACL_1428 | 10,71 | 3 | 162,98 |
| ACL_1429 | 13 | 3 | 128,89 |
| ACL_1430 | 53,17 | 9 | 530,16 |
| ACL_1431 | 9,57 | 3 | 211,63 |
