# Supplementary material for: Core Proteome of the Minimal Cell: Comparative Proteomics of Three Mollicute Species
Source: PLoS One. 2011 Jul 19;6(7):e21964. doi: 10.1371/journal.pone.0021964 (PMC3139596; doi:10.1371/journal.pone.0021964)
Supplement: Table S9 — Peptide coverage of predicted Mycoplasma gallisepticum ORFs. (DOC) [file pone.0021964.s009.doc]

Table S9. Peptide coverage of predicted Mycoplasma gallisepticum ORFs.

| **Locus Tag** | **Coverage (% of sequence)** | **Peptides count** | **Protein score** |
| --- | --- | --- | --- |
| MGA_0001 | 17,4 | 4 | 167,51 |
| MGA_0002 | 5,44 | 3 | 151,77 |
| MGA_0004 | 17 | 9 | 312,68 |
| MGA_0009 | 39 | 7 | 312,71 |
| MGA_0011 | 87 | 10 | 695,32 |
| MGA_0012 | 18,48 | 9 | 399,42 |
| MGA_0013 | 18,59 | 11 | 388,83 |
| MGA_0015 | 19,61 | 7 | 307,99 |
| MGA_0023 | 15 | 2 | 57,03 |
| MGA_0027 | 11,04 | 4 | 142,35 |
| MGA_0029 | 7,57 | 2 | 85,86 |
| MGA_0030 | 18,42 | 3 | 135,09 |
| MGA_0035 | 21,65 | 4 | 232,06 |
| MGA_0037 | 15,86 | 7 | 302,25 |
| MGA_0039 | 15,58 | 4 | 162,81 |
| MGA_0041 | 85 | 9 | 620,7 |
| MGA_0042 | 13,01 | 2 | 76,01 |
| MGA_0043 | 37 | 9 | 414,43 |
| MGA_0045 | 6,84 | 4 | 160,25 |
| MGA_0047 | 26,34 | 4 | 183,48 |
| MGA_0048 | 15 | 9 | 332,22 |
| MGA_0051 | 38 | 5 | 266,23 |
| MGA_0053 | 14 | 4 | 136,89 |
| MGA_0056 | 13,3 | 9 | 398,79 |
| MGA_0060 | 11,11 | 4 | 201,68 |
| MGA_0061 | 17,65 | 2 | 66,88 |
| MGA_0063 | 1,43 | 2 | 40,1 |
| MGA_0068 | 45 | 19 | 613,21 |
| MGA_0069 | 66 | 27 | 796,08 |
| MGA_0071 | 57 | 26 | 1020,52 |
| MGA_0073 | 12 | 3 | 77,38 |
| MGA_0085 | 28,57 | 4 | 244,1 |
| MGA_0087 | 4,35 | 2 | 63,98 |
| MGA_0090 | 23,56 | 3 | 111,24 |
| MGA_0091 | 47,85 | 7 | 424,34 |
| MGA_0093 | 28,72 | 6 | 246,19 |
| MGA_0100 | 18,51 | 8 | 340 |
| MGA_0101 | 25,82 | 10 | 540,58 |
| MGA_0104 | 18,08 | 5 | 264,82 |
| MGA_0105 | 38,43 | 14 | 681,76 |
| MGA_0106 | 13,08 | 2 | 73,07 |
| MGA_0110 | 86 | 25 | 970,53 |
| MGA_0112 | 38 | 3 | 110,46 |
| MGA_0114 | 54,04 | 16 | 961,37 |
| MGA_0116 | 25,25 | 10 | 484,99 |
| MGA_0117 | 28 | 11 | 370,56 |
| MGA_0119 | 24,29 | 5 | 142,84 |
| MGA_0121 | 22,21 | 12 | 600,81 |
| MGA_0124 | 26,11 | 6 | 326,6 |
| MGA_0125 | 14,01 | 4 | 118,05 |
| MGA_0126 | 24 | 6 | 303,02 |
| MGA_0128 | 23,04 | 6 | 326,63 |
| MGA_0129 | 27,1 | 6 | 222,98 |
| MGA_0131 | 9,33 | 2 | 84,09 |
| MGA_0134 | 39 | 6 | 168,23 |
| MGA_0135 | 25,65 | 11 | 501,61 |
| MGA_0137 | 9,51 | 4 | 227,69 |
| MGA_0141 | 11,07 | 5 | 254,57 |
| MGA_0145 | 28 | 8 | 368,71 |
| MGA_0147 | 7 | 2 | 44,62 |
| MGA_0149 | 9,76 | 2 | 92,7 |
| MGA_0152 | 91 | 34 | 3434,01 |
| MGA_0156 | 24,61 | 11 | 629,3 |
| MGA_0157 | 27,33 | 7 | 398,26 |
| MGA_0158 | 30,21 | 8 | 363,36 |
| MGA_0161 | 9,85 | 4 | 268,69 |
| MGA_0162 | 17,95 | 7 | 357,33 |
| MGA_0164 | 53,31 | 15 | 823,51 |
| MGA_0165 | 27,58 | 6 | 409,87 |
| MGA_0167 | 18,86 | 7 | 359,15 |
| MGA_0169 | 52,56 | 15 | 859,5 |
| MGA_0171 | 8 | 4 | 101,48 |
| MGA_0174 | 13,12 | 3 | 101,88 |
| MGA_0175 | 13 | 2 | 71,49 |
| MGA_0178 | 32,5 | 18 | 854,05 |
| MGA_0180 | 5,08 | 3 | 100,05 |
| MGA_0181 | 41 | 8 | 355,16 |
| MGA_0183 | 31,89 | 10 | 540,29 |
| MGA_0188 | 8,24 | 2 | 45,2 |
| MGA_0190 | 14,94 | 4 | 138,11 |
| MGA_0192 | 7,2 | 3 | 103,37 |
| MGA_0194 | 29,71 | 4 | 148,74 |
| MGA_0195 | 14,43 | 5 | 146,2 |
| MGA_0205 | 4,58 | 8 | 399,59 |
| MGA_0209 | 30,11 | 9 | 549,88 |
| MGA_0211 | 13 | 4 | 123,21 |
| MGA_0212 | 45 | 5 | 184,83 |
| MGA_0214 | 17,42 | 2 | 108,38 |
| MGA_0216 | 22,34 | 3 | 157,1 |
| MGA_0218 | 17,46 | 14 | 709,2 |
| MGA_0220 | 17,12 | 5 | 162,68 |
| MGA_0226 | 24 | 14 | 665,93 |
| MGA_0230 | 30,92 | 11 | 610,02 |
| MGA_0232 | 19,55 | 6 | 301,39 |
| MGA_0234 | 8,33 | 2 | 81,12 |
| MGA_0235 | 11,71 | 4 | 198,69 |
| MGA_0237 | 24,36 | 20 | 948,03 |
| MGA_0241 | 21,22 | 27 | 1511,79 |
| MGA_0242 | 26,32 | 7 | 328,02 |
| MGA_0243 | 19,81 | 4 | 169,78 |
| MGA_0244 | 22,4 | 6 | 334,95 |
| MGA_0248 | 5,33 | 2 | 71,21 |
| MGA_0249 | 52 | 11 | 472,32 |
| MGA_0250 | 5 | 1 | 23,88 |
| MGA_0252 | 31,43 | 5 | 223,43 |
| MGA_0254 | 35,85 | 5 | 263,48 |
| MGA_0260 | 26,76 | 11 | 629,22 |
| MGA_0261 | 43,87 | 6 | 305,69 |
| MGA_0262 | 70 | 8 | 321,07 |
| MGA_0264 | 13,53 | 3 | 139,39 |
| MGA_0267 | 15,69 | 7 | 376,48 |
| MGA_0274 | 21 | 12 | 406,17 |
| MGA_0279 | 34,12 | 15 | 1007,33 |
| MGA_0291 | 12,77 | 2 | 128,29 |
| MGA_0293 | 8,57 | 3 | 119,3 |
| MGA_0294 | 19,79 | 3 | 131,48 |
| MGA_0295 | 28,57 | 6 | 276,22 |
| MGA_0297 | 32 | 7 | 197,95 |
| MGA_0306 | 21,36 | 31 | 1452,47 |
| MGA_0319 | 18,86 | 16 | 803,98 |
| MGA_0321 | 37,35 | 31 | 1597,45 |
| MGA_0332 | 58 | 9 | 491,08 |
| MGA_0335 | 18 | 6 | 218,27 |
| MGA_0336 | 12,42 | 3 | 100,93 |
| MGA_0338 | 16,26 | 13 | 471,61 |
| MGA_0339 | 17,99 | 3 | 89,69 |
| MGA_0342 | 12,94 | 5 | 300,91 |
| MGA_0343 | 11,99 | 3 | 111,22 |
| MGA_0346 | 23,96 | 3 | 105,42 |
| MGA_0348 | 19,66 | 5 | 192,86 |
| MGA_0351 | 6,17 | 2 | 59,14 |
| MGA_0353 | 10,38 | 2 | 150,96 |
| MGA_0354 | 20,43 | 5 | 195,77 |
| MGA_0356 | 34 | 11 | 492,73 |
| MGA_0357 | 21,83 | 4 | 164,22 |
| MGA_0358 | 35,6 | 15 | 589,14 |
| MGA_0362 | 4,77 | 2 | 85,65 |
| MGA_0363 | 27,43 | 4 | 173,55 |
| MGA_0364 | 48,1 | 8 | 374,07 |
| MGA_0368 | 22 | 8 | 203,48 |
| MGA_0372 | 7,94 | 4 | 93,58 |
| MGA_0379 | 8,64 | 5 | 224,38 |
| MGA_0380 | 20,16 | 10 | 431,87 |
| MGA_0383 | 37 | 16 | 424,85 |
| MGA_0386 | 5,23 | 2 | 83,2 |
| MGA_0388 | 4,36 | 2 | 62,43 |
| MGA_0390 | 59 | 25 | 937,13 |
| MGA_0393 | 38 | 16 | 436,09 |
| MGA_0395 | 42 | 21 | 651,64 |
| MGA_0398 | 9,76 | 4 | 214,81 |
| MGA_0399 | 8,92 | 2 | 80,59 |
| MGA_0400 | 36,7 | 2 | 103,72 |
| MGA_0401 | 10,58 | 4 | 151,37 |
| MGA_0403 | 20,56 | 7 | 304,31 |
| MGA_0409 | 5,33 | 2 | 129,89 |
| MGA_0412 | 18,92 | 9 | 353,22 |
| MGA_0413 | 6,64 | 2 | 94,09 |
| MGA_0414 | 13 | 4 | 98,87 |
| MGA_0416 | 11,57 | 5 | 181,02 |
| MGA_0419 | 20,59 | 9 | 318,66 |
| MGA_0420 | 31,29 | 5 | 255,96 |
| MGA_0421 | 9,26 | 2 | 97,76 |
| MGA_0422 | 16,83 | 3 | 145 |
| MGA_0423 | 27 | 5 | 265,17 |
| MGA_0424 | 23,71 | 6 | 254,92 |
| MGA_0428 | 20,17 | 7 | 356 |
| MGA_0431 | 18,97 | 4 | 216,1 |
| MGA_0432 | 46,06 | 10 | 497,27 |
| MGA_0433 | 36 | 7 | 272,06 |
| MGA_0438 | 14,17 | 2 | 82,03 |
| MGA_0439 | 27 | 5 | 253,02 |
| MGA_0441 | 33,75 | 3 | 179,29 |
| MGA_0443 | 34,41 | 9 | 424,58 |
| MGA_0447 | 37,9 | 5 | 267,38 |
| MGA_0452 | 34,29 | 4 | 205,86 |
| MGA_0454 | 11,96 | 7 | 319,18 |
| MGA_0455 | 18,98 | 3 | 123,39 |
| MGA_0457 | 43,79 | 16 | 782,34 |
| MGA_0458 | 47 | 9 | 285,68 |
| MGA_0459 | 7,31 | 2 | 72,51 |
| MGA_0461 | 26,79 | 5 | 203,4 |
| MGA_0462 | 21,03 | 3 | 185,34 |
| MGA_0463 | 40,38 | 8 | 375,17 |
| MGA_0468 | 26 | 8 | 238,2 |
| MGA_0469 | 11,58 | 4 | 225,45 |
| MGA_0470 | 13,64 | 3 | 164,56 |
| MGA_0471 | 29 | 6 | 286,87 |
| MGA_0472 | 5 | 1 | 25,15 |
| MGA_0473 | 50 | 9 | 401,77 |
| MGA_0475 | 16,25 | 4 | 142,49 |
| MGA_0480 | 24,7 | 3 | 124,91 |
| MGA_0488 | 27,06 | 10 | 405,65 |
| MGA_0491 | 23,77 | 8 | 373,09 |
| MGA_0493 | 30 | 12 | 425,4 |
| MGA_0495 | 10,87 | 5 | 141,42 |
| MGA_0497 | 40,85 | 5 | 197,68 |
| MGA_0498 | 49,83 | 9 | 514,65 |
| MGA_0500 | 17,17 | 5 | 129,75 |
| MGA_0502 | 9,25 | 2 | 90,06 |
| MGA_0503 | 23,68 | 2 | 96,15 |
| MGA_0504 | 21,24 | 4 | 271,48 |
| MGA_0506 | 43 | 7 | 282,49 |
| MGA_0516 | 5 | 2 | 49,42 |
| MGA_0517 | 3 | 1 | 37,9 |
| MGA_0519 | 11,93 | 15 | 555,33 |
| MGA_0526 | 7,55 | 2 | 63,26 |
| MGA_0532 | 22 | 3 | 123,23 |
| MGA_0533 | 14,29 | 5 | 207,95 |
| MGA_0535 | 5,91 | 4 | 256,56 |
| MGA_0536 | 29,57 | 8 | 368,3 |
| MGA_0541 | 3,04 | 2 | 72,55 |
| MGA_0551 | 27,76 | 6 | 194,88 |
| MGA_0565 | 18,77 | 11 | 587,79 |
| MGA_0569 | 6,28 | 4 | 186,3 |
| MGA_0570 | 4,68 | 4 | 99,64 |
| MGA_0571 | 35,96 | 5 | 249,59 |
| MGA_0573 | 18,88 | 3 | 169,95 |
| MGA_0579 | 15,57 | 9 | 405,36 |
| MGA_0586 | 26 | 15 | 379,59 |
| MGA_0590 | 21,22 | 9 | 368,44 |
| MGA_0591 | 8,72 | 3 | 161,96 |
| MGA_0594 | 15,46 | 6 | 312,14 |
| MGA_0596 | 19,61 | 5 | 203,78 |
| MGA_0597 | 9 | 2 | 47,47 |
| MGA_0599 | 25,87 | 7 | 344,14 |
| MGA_0600 | 4 | 1 | 50,02 |
| MGA_0603 | 27,72 | 4 | 184,15 |
| MGA_0604 | 29,74 | 10 | 443,05 |
| MGA_0605 | 35 | 8 | 282,12 |
| MGA_0606 | 18,78 | 2 | 124,64 |
| MGA_0608 | 70 | 24 | 1114,36 |
| MGA_0612 | 5,32 | 4 | 169,52 |
| MGA_0616 | 6,94 | 3 | 126,22 |
| MGA_0617 | 26,27 | 8 | 302,93 |
| MGA_0618 | 22,61 | 5 | 384,17 |
| MGA_0619 | 20,91 | 6 | 213,6 |
| MGA_0621 | 32,72 | 6 | 312,44 |
| MGA_0622 | 4,99 | 2 | 66,19 |
| MGA_0630 | 15,5 | 2 | 112,39 |
| MGA_0633 | 8,18 | 2 | 57,32 |
| MGA_0636 | 26,5 | 11 | 516,97 |
| MGA_0637 | 5,72 | 2 | 53,9 |
| MGA_0646 | 13,05 | 4 | 216,66 |
| MGA_0648 | 17,68 | 9 | 403,52 |
| MGA_0649 | 17,65 | 2 | 106,48 |
| MGA_0652 | 13,61 | 6 | 291,19 |
| MGA_0654 | 41 | 39 | 1069,52 |
| MGA_0655 | 8,22 | 3 | 122,82 |
| MGA_0658 | 47,8 | 7 | 430,82 |
| MGA_0659 | 32,29 | 16 | 751,66 |
| MGA_0661 | 3 | 1 | 30,42 |
| MGA_0662 | 10 | 3 | 104,44 |
| MGA_0664 | 42,86 | 3 | 173,4 |
| MGA_0666 | 15,47 | 7 | 447,92 |
| MGA_0670 | 7,64 | 5 | 284,89 |
| MGA_0674 | 27,56 | 13 | 616,32 |
| MGA_0676 | 20,65 | 3 | 167,17 |
| MGA_0677 | 55 | 22 | 768,36 |
| MGA_0682 | 11 | 2 | 55,66 |
| MGA_0683 | 14,86 | 3 | 166,88 |
| MGA_0686 | 19,77 | 12 | 422,04 |
| MGA_0687 | 13,77 | 4 | 126,64 |
| MGA_0693 | 15,03 | 4 | 133,71 |
| MGA_0694 | 32,07 | 6 | 293,19 |
| MGA_0695 | 24,38 | 16 | 845,9 |
| MGA_0696 | 11,54 | 2 | 106,04 |
| MGA_0698 | 10,03 | 3 | 142,26 |
| MGA_0699 | 8,65 | 2 | 77,14 |
| MGA_0701 | 6,78 | 2 | 83,79 |
| MGA_0705 | 21,43 | 4 | 109,32 |
| MGA_0707 | 16,53 | 3 | 150,38 |
| MGA_0710 | 82 | 12 | 1056,81 |
| MGA_0711 | 74 | 5 | 230,57 |
| MGA_0712 | 11,31 | 3 | 118,47 |
| MGA_0714 | 28,74 | 3 | 143,89 |
| MGA_0716 | 38,19 | 4 | 205,21 |
| MGA_0717 | 87 | 17 | 896,51 |
| MGA_0721 | 24,64 | 3 | 137,08 |
| MGA_0723 | 13,64 | 2 | 102,17 |
| MGA_0725 | 27,06 | 2 | 72,55 |
| MGA_0726 | 94 | 11 | 495,75 |
| MGA_0728 | 22,94 | 2 | 69,8 |
| MGA_0729 | 34,41 | 6 | 295,86 |
| MGA_0733 | 42,86 | 5 | 224,29 |
| MGA_0734 | 12,02 | 2 | 72,25 |
| MGA_0735 | 50,42 | 5 | 204,78 |
| MGA_0737 | 10,13 | 2 | 76,84 |
| MGA_0739 | 44 | 6 | 299,3 |
| MGA_0740 | 7,23 | 3 | 67,75 |
| MGA_0743 | 33,18 | 6 | 259,91 |
| MGA_0746 | 37,77 | 11 | 556,11 |
| MGA_0753 | 15,93 | 7 | 341,7 |
| MGA_0758 | 15,26 | 4 | 130,61 |
| MGA_0762 | 47 | 9 | 382,29 |
| MGA_0763 | 27,54 | 10 | 427,97 |
| MGA_0766 | 48,89 | 4 | 179,65 |
| MGA_0768 | 18,23 | 10 | 464,8 |
| MGA_0771 | 43 | 19 | 690,75 |
| MGA_0774 | 58,72 | 6 | 259,44 |
| MGA_0777 | 1,72 | 2 | 37,37 |
| MGA_0778 | 26,83 | 19 | 820,83 |
| MGA_0782 | 87 | 24 | 2477,22 |
| MGA_0783 | 24,79 | 4 | 199,07 |
| MGA_0784 | 40,86 | 6 | 281 |
| MGA_0787 | 8,42 | 3 | 85,08 |
| MGA_0791 | 4,33 | 6 | 262,84 |
| MGA_0793 | 32,86 | 29 | 1167,49 |
| MGA_0806 | 22,78 | 3 | 94,27 |
| MGA_0808 | 7,72 | 2 | 47,47 |
| MGA_0809 | 12,43 | 3 | 180,07 |
| MGA_0810 | 36 | 7 | 268,41 |
| MGA_0811 | 10 | 2 | 58,59 |
| MGA_0818 | 16,16 | 8 | 286,62 |
| MGA_0821 | 5,69 | 2 | 107,64 |
| MGA_0824 | 42,98 | 4 | 193,97 |
| MGA_0829 | 6,44 | 2 | 105,14 |
| MGA_0833 | 12,56 | 4 | 218,88 |
| MGA_0834 | 24,32 | 18 | 899,66 |
| MGA_0838 | 8,52 | 2 | 75,96 |
| MGA_0839 | 38 | 10 | 290,32 |
| MGA_0843 | 8,81 | 2 | 69,16 |
| MGA_0848 | 16,05 | 2 | 111,87 |
| MGA_0849 | 24 | 10 | 367,03 |
| MGA_0851 | 15,67 | 2 | 100,18 |
| MGA_0855 | 9,98 | 7 | 366,65 |
| MGA_0860 | 28,76 | 11 | 643,58 |
| MGA_0863 | 18,18 | 6 | 191,1 |
| MGA_0864 | 22 | 5 | 157,39 |
| MGA_0866 | 33,33 | 4 | 151,15 |
| MGA_0869 | 36,44 | 4 | 214,13 |
| MGA_0870 | 48,68 | 6 | 361,97 |
| MGA_0872 | 89 | 13 | 789,34 |
| MGA_0874 | 25,62 | 3 | 150,52 |
| MGA_0877 | 10,86 | 4 | 210,04 |
| MGA_0878 | 18,45 | 7 | 249,55 |
| MGA_0879 | 13 | 7 | 184,45 |
| MGA_0884 | 20,41 | 3 | 162,74 |
| MGA_0887 | 22,98 | 7 | 290,26 |
| MGA_0893 | 11,02 | 5 | 227,27 |
| MGA_0898 | 4,8 | 2 | 85,97 |
| MGA_0900 | 57 | 10 | 396,92 |
| MGA_0901 | 10,75 | 2 | 139,64 |
| MGA_0902 | 57,07 | 8 | 384,43 |
| MGA_0910 | 4,51 | 2 | 52,77 |
| MGA_0911 | 5 | 2 | 53,97 |
| MGA_0913 | 12,09 | 3 | 146,82 |
| MGA_0914 | 14,05 | 3 | 120,53 |
| MGA_0916 | 10,63 | 3 | 122,81 |
| MGA_0917 | 9,87 | 9 | 374,2 |
| MGA_0919 | 30,95 | 12 | 484,47 |
| MGA_0923 | 30,58 | 6 | 239,02 |
| MGA_0925 | 11,34 | 5 | 284,99 |
| MGA_0927 | 11,83 | 3 | 127,65 |
| MGA_0928 | 4,07 | 4 | 142,62 |
| MGA_0931 | 18,55 | 5 | 228,51 |
| MGA_0932 | 61 | 12 | 447,77 |
| MGA_0934 | 20,84 | 17 | 844,94 |
| MGA_0939 | 19,59 | 15 | 760,6 |
| MGA_0947 | 25,3 | 10 | 452,9 |
| MGA_0948 | 4,98 | 2 | 118,04 |
| MGA_0950 | 3,73 | 2 | 66,87 |
| MGA_0953 | 5 | 2 | 91,6 |
| MGA_0954 | 3,65 | 2 | 49,55 |
| MGA_0955 | 42,13 | 6 | 299,98 |
| MGA_0958 | 28,3 | 3 | 132,08 |
| MGA_0963 | 5 | 2 | 69,71 |
| MGA_0965 | 33,33 | 4 | 249,27 |
| MGA_0966 | 11,35 | 5 | 256,95 |
| MGA_0972 | 4,86 | 2 | 145,12 |
| MGA_0973 | 34 | 15 | 473,67 |
| MGA_0974 | 3,01 | 2 | 51,49 |
| MGA_0977 | 3 | 2 | 83,26 |
| MGA_0981 | 2,82 | 2 | 93,93 |
| MGA_0986 | 2,9 | 2 | 69,97 |
| MGA_0987 | 36 | 15 | 433,27 |
| MGA_0993 | 7,07 | 3 | 137,85 |
| MGA_0995 | 13,27 | 2 | 61,58 |
| MGA_0996 | 8,01 | 2 | 87,1 |
| MGA_0998 | 18,65 | 3 | 164,59 |
| MGA_0999 | 92 | 19 | 1181,33 |
| MGA_1000 | 22,49 | 22 | 1121,4 |
| MGA_1005 | 20,06 | 17 | 755,17 |
| MGA_1014 | 39 | 11 | 508,12 |
| MGA_1017 | 12,02 | 3 | 178,34 |
| MGA_1018 | 23,39 | 5 | 191,25 |
| MGA_1022 | 6,54 | 2 | 53,07 |
| MGA_1033 | 82 | 27 | 3800,78 |
| MGA_1034 | 22,8 | 7 | 358,97 |
| MGA_1036 | 16,9 | 3 | 130,16 |
| MGA_1047 | 25 | 2 | 142,17 |
| MGA_1048 | 15,54 | 14 | 566,12 |
| MGA_1052 | 16,78 | 4 | 177,29 |
| MGA_1053 | 42 | 8 | 508,6 |
| MGA_1054n | 27 | 3 | 194,17 |
| MGA_1055 | 12,98 | 2 | 63,07 |
| MGA_1059 | 23 | 10 | 327,88 |
| MGA_1061 | 1,93 | 2 | 36,82 |
| MGA_1065 | 33,92 | 14 | 550,73 |
| MGA_1068 | 31,01 | 5 | 182,34 |
| MGA_1070 | 8,95 | 2 | 91,4 |
| MGA_1071 | 4,69 | 2 | 66,17 |
| MGA_1073 | 23,75 | 11 | 474,28 |
| MGA_1076 | 7,33 | 2 | 85,65 |
| MGA_1079 | 4,31 | 5 | 193,97 |
| MGA_1081 | 4,52 | 2 | 52,77 |
| MGA_1085 | 5,57 | 3 | 142,27 |
| MGA_1088 | 16,74 | 3 | 159,63 |
| MGA_1089 | 4 | 1 | 24,37 |
| MGA_1102 | 16 | 8 | 208,65 |
| MGA_1110 | 29,59 | 4 | 156,24 |
| MGA_1111 | 12,8 | 2 | 64,38 |
| MGA_1112 | 14,72 | 5 | 242,65 |
| MGA_1115 | 8,66 | 2 | 50,35 |
| MGA_1116 | 9,76 | 2 | 114,88 |
| MGA_1119 | 5 | 2 | 54,25 |
| MGA_1121 | 22,46 | 4 | 116,12 |
| MGA_1125 | 20,52 | 5 | 235,71 |
| MGA_1127 | 19,63 | 4 | 171,1 |
| MGA_1128 | 10,75 | 4 | 212,32 |
| MGA_1130 | 6,87 | 2 | 43,27 |
| MGA_1131 | 10 | 2 | 62,48 |
| MGA_1138 | 33,91 | 10 | 534,25 |
| MGA_1140 | 17,89 | 6 | 298,81 |
| MGA_1142 | 67,55 | 7 | 269,73 |
| MGA_1143 | 11,97 | 4 | 133,62 |
| MGA_1144 | 20 | 7 | 268,94 |
| MGA_1146 | 9,9 | 2 | 58,61 |
| MGA_1153 | 7,24 | 4 | 112,43 |
| MGA_1154 | 94 | 11 | 584,88 |
| MGA_1156 | 15 | 5 | 134,84 |
| MGA_1159 | 59,72 | 9 | 502,55 |
| MGA_1162 | 14,05 | 5 | 195,8 |
| MGA_1168 | 19,57 | 5 | 327,38 |
| MGA_1170 | 46,41 | 7 | 355,3 |
| MGA_1172 | 11,39 | 5 | 282,49 |
| MGA_1174 | 8,65 | 2 | 69,31 |
| MGA_1177 | 28,87 | 10 | 485,28 |
| MGA_1179 | 33,08 | 3 | 133,87 |
| MGA_1180 | 6,47 | 2 | 55,06 |
| MGA_1182 | 19,23 | 5 | 238,34 |
| MGA_1184 | 36,39 | 8 | 478,28 |
| MGA_1186 | 25,15 | 7 | 288,63 |
| MGA_1187 | 34,38 | 10 | 480,65 |
| MGA_1189 | 12,5 | 4 | 172,1 |
| MGA_1191 | 4,28 | 3 | 69,41 |
| MGA_1196 | 17,98 | 13 | 609,05 |
| MGA_1199 | 7,01 | 5 | 211,73 |
| MGA_1203 | 3,26 | 5 | 211,73 |
| MGA_1208 | 17,55 | 12 | 614,07 |
| MGA_1210 | 11 | 3 | 182,36 |
| MGA_1211 | 34,8 | 6 | 321,66 |
| MGA_1212 | 8,18 | 5 | 256,4 |
| MGA_1218 | 30,08 | 6 | 262,29 |
| MGA_1220 | 23,77 | 7 | 347,36 |
| MGA_1221 | 18,9 | 4 | 163,53 |
| MGA_1222 | 7,98 | 3 | 147,79 |
| MGA_1224 | 18,61 | 11 | 489,28 |
| MGA_1227 | 2,67 | 2 | 44,36 |
| MGA_1228 | 10,2 | 7 | 337,95 |
| MGA_1232 | 8,5 | 2 | 88,57 |
| MGA_1238 | 3,61 | 2 | 124,63 |
| MGA_1239 | 70 | 31 | 869,61 |
| MGA_1245 | 9,78 | 5 | 241,81 |
| MGA_1246 | 40 | 18 | 599,98 |
| MGA_1249 | 6,02 | 3 | 139,49 |
| MGA_1250 | 6,2 | 4 | 191,56 |
| MGA_1251 | 61 | 25 | 721,75 |
| MGA_1253 | 19 | 8 | 208,29 |
| MGA_1257 | 24 | 10 | 298,94 |
| MGA_1261 | 3,57 | 2 | 125,84 |
| MGA_1269 | 34 | 14 | 415,55 |
| MGA_1270 | 9,39 | 3 | 117,18 |
| MGA_1272 | 18,02 | 3 | 199,11 |
| MGA_1274 | 34,78 | 3 | 136,5 |
| MGA_1275 | 15,64 | 3 | 133,08 |
| MGA_1278 | 10,03 | 3 | 140,88 |
| MGA_1281 | 19,23 | 12 | 634,59 |
| MGA_1285 | 13 | 3 | 99,24 |
| MGA_1290 | 17,76 | 2 | 112,23 |
| MGA_1293 | 12,54 | 3 | 108,54 |
| MGA_1296 | 6,76 | 2 | 80,08 |
| MGA_1297 | 33,11 | 11 | 561,28 |
| MGA_1299 | 38,01 | 23 | 1122,29 |
| MGA_1303 | 31 | 7 | 275,56 |
| MGA_1309 | 26,74 | 7 | 313,44 |
| MGA_1313 | 22,97 | 13 | 690,5 |
| MGA_1331d | 5 | 2 | 62,44 |
