# Supplementary material for: Core Proteome of the Minimal Cell: Comparative Proteomics of Three Mollicute Species
Source: PLoS One. 2011 Jul 19;6(7):e21964. doi: 10.1371/journal.pone.0021964 (PMC3139596; doi:10.1371/journal.pone.0021964)
Supplement: Table S10 — COG's found in the core genome (the whole table) and the core proteome (highlighted in the “Core Proteome” column). (DOC) [file pone.0021964.s010.doc]

Table S10. COG’s found in the core genome (the whole table) and the core proteome (highlighted in the “Core Proteome” column).

| **COG** | **COG Category** | **Core Proteome** | **Function** | **Dispensable in M. genitallium** |
| --- | --- | --- | --- | --- |
| COG0006 | E | 1 | Xaa-Pro aminopeptidase | 0 |
| COG0008 | J | 1 | Glutamyl- and glutaminyl-tRNA synthetases | 0 |
| COG0013 | J | 1 | Alanyl-tRNA synthetase | 0 |
| COG0016 | J | 1 | Phenylalanyl-tRNA synthetase alpha subunit | 0 |
| COG0017 | J | 1 | Aspartyl/asparaginyl-tRNA synthetases | 0 |
| COG0018 | J | 1 | Arginyl-tRNA synthetase | 0 |
| COG0022 | C | 1 | Pyruvate/2-oxoglutarate dehydrogenase complex, dehydrogenase (E1) component, eukaryotic type, beta subunit | 0 |
| COG0024 | J | 1 | Methionine aminopeptidase | 0 |
| COG0035 | F | 1 | Uracil phosphoribosyltransferase | 0 |
| COG0048 | J | 1 | Ribosomal protein S12 | 0 |
| COG0049 | J | 1 | Ribosomal protein S7 | 0 |
| COG0050 | J | 1 | GTPases - translation elongation factors | 0 |
| COG0051 | J | 1 | Ribosomal protein S10 | 0 |
| COG0052 | J | 1 | Ribosomal protein S2 | 0 |
| COG0055 | C | 1 | F0F1-type ATP synthase, beta subunit | 0 |
| COG0056 | C | 1 | F0F1-type ATP synthase, alpha subunit | 0 |
| COG0057 | G | 1 | Glyceraldehyde-3-phosphate dehydrogenase/erythrose-4-phosphate dehydrogenase | 0 |
| COG0060 | J | 1 | Isoleucyl-tRNA synthetase | 0 |
| COG0072 | J | 1 | Phenylalanyl-tRNA synthetase beta subunit | 0 |
| COG0080 | J | 1 | Ribosomal protein L11 | 0 |
| COG0081 | J | 1 | Ribosomal protein L1 | 0 |
| COG0085 | K | 1 | DNA-directed RNA polymerase, beta subunit/140 kD subunit | 0 |
| COG0086 | K | 1 | DNA-directed RNA polymerase, beta' subunit/160 kD subunit | 0 |
| COG0087 | J | 1 | Ribosomal protein L3 | 0 |
| COG0088 | J | 1 | Ribosomal protein L4 | 0 |
| COG0089 | J | 1 | Ribosomal protein L23 | 0 |
| COG0090 | J | 1 | Ribosomal protein L2 | 0 |
| COG0091 | J | 1 | Ribosomal protein L22 | 0 |
| COG0092 | J | 1 | Ribosomal protein S3 | 0 |
| COG0093 | J | 1 | Ribosomal protein L14 | 0 |
| COG0094 | J | 1 | Ribosomal protein L5 | 0 |
| COG0095 | H | 1 | Lipoate-protein ligase A | 0 |
| COG0096 | J | 1 | Ribosomal protein S8 | 0 |
| COG0097 | J | 1 | Ribosomal protein L6P/L9E | 0 |
| COG0098 | J | 1 | Ribosomal protein S5 | 0 |
| COG0099 | J | 1 | Ribosomal protein S13 | 0 |
| COG0102 | J | 1 | Ribosomal protein L13 | 0 |
| COG0103 | J | 1 | Ribosomal protein S9 | 0 |
| COG0112 | E | 1 | Glycine/serine hydroxymethyltransferase | 0 |
| COG0124 | J | 1 | Histidyl-tRNA synthetase | 0 |
| COG0125 | F | 1 | Thymidylate kinase | 0 |
| COG0126 | G | 1 | 3-phosphoglycerate kinase | 0 |
| COG0143 | J | 1 | Methionyl-tRNA synthetase | 0 |
| COG0148 | G | 1 | Enolase | 0 |
| COG0149 | G | 1 | Triosephosphate isomerase | 0 |
| COG0162 | J | 1 | Tyrosyl-tRNA synthetase | 0 |
| COG0172 | J | 1 | Seryl-tRNA synthetase | 0 |
| COG0173 | J | 1 | Aspartyl-tRNA synthetase | 0 |
| COG0178 | L | 1 | Excinuclease ATPase subunit | 0 |
| COG0180 | J | 1 | Tryptophanyl-tRNA synthetase | 0 |
| COG0184 | J | 1 | Ribosomal protein S15P/S13E | 0 |
| COG0187 | L | 1 | Type IIA topoisomerase (DNA gyrase/topo II, topoisomerase IV), B subunit | 0 |
| COG0188 | L | 1 | Type IIA topoisomerase (DNA gyrase/topo II, topoisomerase IV), A subunit | 0 |
| COG0190 | H | 1 | 5,10-methylene-tetrahydrofolate dehydrogenase/Methenyl tetrahydrofolate cyclohydrolase | 0 |
| COG0191 | G | 1 | Fructose/tagatose bisphosphate aldolase | 0 |
| COG0192 | H | 1 | S-adenosylmethionine synthetase | 0 |
| COG0193 | J | 1 | Peptidyl-tRNA hydrolase | 0 |
| COG0194 | F | 1 | Guanylate kinase | 0 |
| COG0195 | K | 1 | Transcription elongation factor | 0 |
| COG0197 | J | 1 | Ribosomal protein L16/L10E | 0 |
| COG0198 | J | 1 | Ribosomal protein L24 | 0 |
| COG0200 | J | 1 | Ribosomal protein L15 | 0 |
| COG0202 | K | 1 | DNA-directed RNA polymerase, alpha subunit/40 kD subunit | 0 |
| COG0203 | J | 1 | Ribosomal protein L17 | 0 |
| COG0204 | I | 1 | 1-acyl-sn-glycerol-3-phosphate acyltransferase | 0 |
| COG0205 | G | 1 | 6-phosphofructokinase | 0 |
| COG0209 | F | 1 | Ribonucleotide reductase, alpha subunit | 0 |
| COG0215 | J | 1 | Cysteinyl-tRNA synthetase | 0 |
| COG0216 | J | 1 | Protein chain release factor A | 0 |
| COG0217 | S | 1 | Uncharacterized conserved protein | 0 |
| COG0218 | R | 1 | Predicted GTPase | 0 |
| COG0221 | C | 1 | Inorganic pyrophosphatase | 0 |
| COG0222 | J | 1 | Ribosomal protein L7/L12 | 0 |
| COG0224 | C | 1 | F0F1-type ATP synthase, gamma subunit | 0 |
| COG0231 | J | 1 | Translation elongation factor P (EF-P)/translation initiation factor 5A (eIF-5A) | 0 |
| COG0233 | J | 1 | Ribosome recycling factor | 0 |
| COG0242 | J | 1 | N-formylmethionyl-tRNA deformylase | 0 |
| COG0244 | J | 1 | Ribosomal protein L10 | 0 |
| COG0250 | K | 1 | Transcription antiterminator | 0 |
| COG0256 | J | 1 | Ribosomal protein L18 | 0 |
| COG0261 | J | 1 | Ribosomal protein L21 | 0 |
| COG0264 | J | 1 | Translation elongation factor Ts | 0 |
| COG0272 | L | 1 | NAD-dependent DNA ligase (contains BRCT domain type II) | 0 |
| COG0274 | F | 1 | Deoxyribose-phosphate aldolase | 0 |
| COG0275 | M | 1 | Predicted S-adenosylmethionine-dependent methyltransferase involved in cell envelope biogenesis | 0 |
| COG0282 | C | 1 | Acetate kinase | 0 |
| COG0283 | F | 1 | Cytidylate kinase | 0 |
| COG0290 | J | 1 | Translation initiation factor 3 (IF-3) | 0 |
| COG0292 | J | 1 | Ribosomal protein L20 | 0 |
| COG0301 | H | 1 | Thiamine biosynthesis ATP pyrophosphatase | 0 |
| COG0305 | L | 1 | Replicative DNA helicase | 0 |
| COG0322 | L | 1 | Nuclease subunit of the excinuclease complex | 0 |
| COG0335 | J | 1 | Ribosomal protein L19 | 0 |
| COG0336 | J | 1 | tRNA-(guanine-N1)-methyltransferase | 0 |
| COG0360 | J | 1 | Ribosomal protein S6 | 0 |
| COG0416 | I | 1 | Fatty acid/phospholipid biosynthesis enzyme | 0 |
| COG0423 | J | 1 | Glycyl-tRNA synthetase (class II) | 0 |
| COG0441 | J | 1 | Threonyl-tRNA synthetase | 0 |
| COG0442 | J | 1 | Prolyl-tRNA synthetase | 0 |
| COG0443 | O | 1 | Molecular chaperone | 0 |
| COG0444 | EP | 1 | ABC-type dipeptide/oligopeptide/nickel transport system, ATPase component | 0 |
| COG0445 | D | 1 | NAD/FAD-utilizing enzyme apparently involved in cell division | 0 |
| COG0446 | R | 1 | Uncharacterized NAD(FAD)-dependent dehydrogenases | 0 |
| COG0462 | FE | 1 | Phosphoribosylpyrophosphate synthetase | 0 |
| COG0465 | O | 1 | ATP-dependent Zn proteases | 0 |
| COG0466 | O | 1 | ATP-dependent Lon protease, bacterial type | 0 |
| COG0469 | G | 1 | Pyruvate kinase | 0 |
| COG0480 | J | 1 | Translation elongation factors (GTPases) | 0 |
| COG0486 | R | 1 | Predicted GTPase | 0 |
| COG0492 | O | 1 | Thioredoxin reductase | 0 |
| COG0495 | J | 1 | Leucyl-tRNA synthetase | 0 |
| COG0503 | F | 1 | Adenine/guanine phosphoribosyltransferases and related PRPP-binding proteins | 0 |
| COG0508 | C | 1 | Pyruvate/2-oxoglutarate dehydrogenase complex, dihydrolipoamide acyltransferase (E2) component, and related enzymes | 0 |
| COG0510 | M | 1 | Predicted choline kinase involved in LPS biosynthesis | 0 |
| COG0513 | LKJ | 1 | Superfamily II DNA and RNA helicases | 0 |
| COG0520 | E | 1 | Selenocysteine lyase | 0 |
| COG0522 | J | 1 | Ribosomal protein S4 and related proteins | 0 |
| COG0525 | J | 1 | Valyl-tRNA synthetase | 0 |
| COG0528 | F | 1 | Uridylate kinase | 0 |
| COG0532 | J | 1 | Translation initiation factor 2 (IF-2; GTPase) | 0 |
| COG0533 | O | 1 | Metal-dependent proteases with possible chaperone activity | 0 |
| COG0536 | R | 1 | Predicted GTPase | 0 |
| COG0537 | FGR | 1 | Diadenosine tetraphosphate (Ap4A) hydrolase and other HIT family hydrolases | 0 |
| COG0541 | U | 1 | Signal recognition particle GTPase | 0 |
| COG0550 | L | 1 | Topoisomerase IA | 0 |
| COG0552 | U | 1 | Signal recognition particle GTPase | 0 |
| COG0556 | L | 1 | Helicase subunit of the DNA excision repair complex | 0 |
| COG0557 | K | 1 | Exoribonuclease R | 0 |
| COG0561 | R | 1 | Predicted hydrolases of the HAD superfamily | 0 |
| COG0563 | F | 1 | Adenylate kinase and related kinases | 0 |
| COG0568 | K | 1 | DNA-directed RNA polymerase, sigma subunit (sigma70/sigma32) | 0 |
| COG0569 | P | 1 | K+ transport systems, NAD-binding component | 0 |
| COG0576 | O | 1 | Molecular chaperone GrpE (heat shock protein) | 0 |
| COG0587 | L | 1 | DNA polymerase III, alpha subunit (gram-negative type) | 0 |
| COG0593 | L | 1 | ATPase involved in DNA replication initiation | 0 |
| COG0595 | R | 1 | Predicted hydrolase of the metallo-beta-lactamase superfamily | 0 |
| COG0596 | R | 1 | Predicted hydrolases or acyltransferases (alpha/beta hydrolase superfamily) | 0 |
| COG0601 | EP | 1 | ABC-type dipeptide/oligopeptide/nickel transport systems, permease components | 0 |
| COG0618 | R | 1 | Exopolyphosphatase-related proteins | 0 |
| COG0629 | L | 1 | Single-stranded DNA-binding protein | 0 |
| COG0634 | F | 1 | Hypoxanthine-guanine phosphoribosyltransferase | 0 |
| COG0648 | L | 1 | Endonuclease IV | 0 |
| COG0653 | U | 1 | Preprotein translocase subunit SecA (ATPase, RNA helicase) | 0 |
| COG0682 | M | 1 | Prolipoprotein diacylglyceryltransferase | 0 |
| COG0691 | O | 1 | tmRNA-binding protein | 0 |
| COG0693 | R | 1 | Putative intracellular protease/amidase | 0 |
| COG0696 | G | 1 | Phosphoglyceromutase | 0 |
| COG0698 | G | 1 | Ribose 5-phosphate isomerase RpiB | 0 |
| COG0704 | P | 1 | Phosphate uptake regulator | 0 |
| COG0711 | C | 1 | F0F1-type ATP synthase, subunit b | 0 |
| COG0776 | L | 1 | Bacterial nucleoid DNA-binding protein | 0 |
| COG0782 | K | 1 | Transcription elongation factor | 0 |
| COG0813 | F | 1 | Purine-nucleoside phosphorylase | 0 |
| COG0858 | J | 1 | Ribosome-binding factor A | 0 |
| COG1071 | C | 1 | Pyruvate/2-oxoglutarate dehydrogenase complex, dehydrogenase (E1) component, eukaryotic type, alpha subunit | 0 |
| COG1080 | G | 1 | Phosphoenolpyruvate-protein kinase (PTS system EI component in bacteria) | 0 |
| COG1109 | G | 1 | Phosphomannomutase | 0 |
| COG1122 | P | 1 | ABC-type cobalt transport system, ATPase component | 0 |
| COG1160 | R | 1 | Predicted GTPases | 0 |
| COG1161 | R | 1 | Predicted GTPases | 0 |
| COG1173 | EP | 1 | ABC-type dipeptide/oligopeptide/nickel transport systems, permease components | 0 |
| COG1190 | J | 1 | Lysyl-tRNA synthetase (class II) | 0 |
| COG1214 | O | 1 | Inactive homolog of metal-dependent proteases, putative molecular chaperone | 0 |
| COG1307 | S | 1 | Uncharacterized protein conserved in bacteria | 0 |
| COG1420 | K | 1 | Transcriptional regulator of heat shock gene | 0 |
| COG1435 | F | 1 | Thymidine kinase | 0 |
| COG1461 | R | 1 | Predicted kinase related to dihydroxyacetone kinase | 0 |
| COG1488 | H | 1 | Nicotinic acid phosphoribosyltransferase | 0 |
| COG1493 | T | 1 | Serine kinase of the HPr protein, regulates carbohydrate metabolism | 0 |
| COG1692 | S | 1 | Uncharacterized protein conserved in bacteria | 0 |
| COG2176 | L | 1 | DNA polymerase III, alpha subunit (gram-positive type) | 0 |
| COG2812 | L | 1 | DNA polymerase III, gamma/tau subunits | 0 |
| COG2890 | J | 1 | Methylase of polypeptide chain release factors | 0 |
| COG3118 | O | 1 | Thioredoxin domain-containing protein | 0 |
| COG3839 | G | 1 | ABC-type sugar transport systems, ATPase components | 0 |
| COG3842 | E | 1 | ABC-type spermidine/putrescine transport systems, ATPase components | 0 |
| COG0012 | J | 1 | Predicted GTPase, probable translation factor | 1 |
| COG0021 | G | 1 | Transketolase | 1 |
| COG0030 | J | 1 | Dimethyladenosine transferase (rRNA methylation) | 1 |
| COG0036 | G | 1 | Pentose-5-phosphate-3-epimerase | 1 |
| COG0039 | C | 1 | Malate/lactate dehydrogenases | 1 |
| COG0084 | L | 1 | Mg-dependent DNase | 1 |
| COG0207 | F | 1 | Thymidylate synthase | 1 |
| COG0210 | L | 1 | Superfamily I DNA and RNA helicases | 1 |
| COG0213 | F | 1 | Thymidine phosphorylase | 1 |
| COG0226 | P | 1 | ABC-type phosphate transport system, periplasmic component | 1 |
| COG0313 | R | 1 | Predicted methyltransferases | 1 |
| COG0358 | L | 1 | DNA primase (bacterial type) | 1 |
| COG0481 | M | 1 | Membrane GTPase LepA | 1 |
| COG0484 | O | 1 | DnaJ-class molecular chaperone with C-terminal Zn finger domain | 1 |
| COG0542 | O | 1 | ATPases with chaperone activity, ATP-binding subunit | 1 |
| COG0544 | O | 1 | FKBP-type peptidyl-prolyl cis-trans isomerase (trigger factor) | 1 |
| COG0564 | J | 1 | Pseudouridylate synthases, 23S RNA-specific | 1 |
| COG0566 | J | 1 | rRNA methylases | 1 |
| COG0571 | K | 1 | dsRNA-specific ribonuclease | 1 |
| COG0718 | S | 1 | Uncharacterized protein conserved in bacteria | 1 |
| COG1112 | L | 1 | Superfamily I DNA and RNA helicases and helicase subunits | 1 |
| COG1117 | P | 1 | ABC-type phosphate transport system, ATPase component | 1 |
| COG1196 | D | 1 | Chromosome segregation ATPases | 1 |
| COG1249 | C | 1 | Pyruvate/2-oxoglutarate dehydrogenase complex, dihydrolipoamide dehydrogenase (E3) component, and related enzymes | 1 |
| COG1466 | L | 1 | DNA polymerase III, delta subunit | 1 |
| COG1744 | R | 1 | Uncharacterized ABC-type transport system, periplasmic component/surface lipoprotein | 1 |
| COG0166 | G | 1 | Glucose-6-phosphate isomerase | - |
| COG0240 | C | 1 | Glycerol-3-phosphate dehydrogenase | - |
| COG0353 | L | 1 | Recombinational DNA repair protein (RecF pathway) | - |
| COG0504 | F | 1 | CTP synthase (UTP-ammonia lyase) | - |
| COG0783 | P | 1 | DNA-binding ferritin-like protein (oxidative damage protectant) | - |
| COG1039 | L | 1 | Ribonuclease HIII | - |
| COG1057 | H | 1 | Nicotinic acid mononucleotide adenylyltransferase | - |
| COG1131 | V | 1 | ABC-type multidrug transport system, ATPase component | - |
| COG1704 | S | 1 | Uncharacterized conserved protein | - |
| COG2256 | L | 1 | ATPase related to the helicase subunit of the Holliday junction resolvase | - |
| COG0100 | J | 0 | Ribosomal protein S11 | 0 |
| COG0168 | P | 0 | Trk-type K+ transport systems, membrane components | 0 |
| COG0185 | J | 0 | Ribosomal protein S19 | 0 |
| COG0186 | J | 0 | Ribosomal protein S17 | 0 |
| COG0199 | J | 0 | Ribosomal protein S14 | 0 |
| COG0201 | U | 0 | Preprotein translocase subunit SecY | 0 |
| COG0211 | J | 0 | Ribosomal protein L27 | 0 |
| COG0220 | R | 0 | Predicted S-adenosylmethionine-dependent methyltransferase | 0 |
| COG0223 | J | 0 | Methionyl-tRNA formyltransferase | 0 |
| COG0227 | J | 0 | Ribosomal protein L28 | 0 |
| COG0228 | J | 0 | Ribosomal protein S16 | 0 |
| COG0229 | O | 0 | Conserved domain frequently associated with peptide methionine sulfoxide reductase | 0 |
| COG0236 | IQ | 0 | Acyl carrier protein | 0 |
| COG0238 | J | 0 | Ribosomal protein S18 | 0 |
| COG0254 | J | 0 | Ribosomal protein L31 | 0 |
| COG0255 | J | 0 | Ribosomal protein L29 | 0 |
| COG0262 | H | 0 | Dihydrofolate reductase | 0 |
| COG0267 | J | 0 | Ribosomal protein L33 | 0 |
| COG0291 | J | 0 | Ribosomal protein L35 | 0 |
| COG0295 | F | 0 | Cytidine deaminase | 0 |
| COG0319 | R | 0 | Predicted metal-dependent hydrolase | 0 |
| COG0333 | J | 0 | Ribosomal protein L32 | 0 |
| COG0344 | S | 0 | Predicted membrane protein | 0 |
| COG0356 | C | 0 | F0F1-type ATP synthase, subunit a | 0 |
| COG0361 | J | 0 | Translation initiation factor 1 (IF-1) | 0 |
| COG0482 | J | 0 | Predicted tRNA(5-methylaminomethyl-2-thiouridylate) methyltransferase, contains the PP-loop ATPase domain | 0 |
| COG0594 | J | 0 | RNase P protein component | 0 |
| COG0619 | P | 0 | ABC-type cobalt transport system, permease component CbiQ and related transporters | 0 |
| COG0692 | L | 0 | Uracil DNA glycosylase | 0 |
| COG0706 | U | 0 | Preprotein translocase subunit YidC | 0 |
| COG0712 | C | 0 | F0F1-type ATP synthase, delta subunit (mitochondrial oligomycin sensitivity protein) | 0 |
| COG0822 | C | 0 | NifU homolog involved in Fe-S cluster formation | 0 |
| COG1132 | V | 0 | ABC-type multidrug transport system, ATPase and permease components | 0 |
| COG1159 | R | 0 | GTPase | 0 |
| COG1175 | G | 0 | ABC-type sugar transport systems, permease components | 0 |
| COG1176 | E | 0 | ABC-type spermidine/putrescine transport system, permease component I | 0 |
| COG1177 | E | 0 | ABC-type spermidine/putrescine transport system, permease component II | 0 |
| COG1284 | S | 0 | Uncharacterized conserved protein | 0 |
| COG1624 | S | 0 | Uncharacterized conserved protein | 0 |
| COG1925 | G | 0 | Phosphotransferase system, HPr-related proteins | 0 |
| COG3611 | L | 0 | Replication initiation/membrane attachment protein | 0 |
| COG4603 | R | 0 | ABC-type uncharacterized transport system, permease component | 0 |
| COG0219 | J | 0 | Predicted rRNA methylase (SpoU class) | 1 |
| COG0225 | O | 0 | Peptide methionine sulfoxide reductase | 1 |
| COG0355 | C | 0 | F0F1-type ATP synthase, epsilon subunit (mitochondrial delta subunit) | 1 |
| COG0357 | M | 0 | Predicted S-adenosylmethionine-dependent methyltransferase involved in bacterial cell division | 1 |
| COG0389 | L | 0 | Nucleotidyltransferase/DNA polymerase involved in DNA repair | 1 |
| COG0468 | L | 0 | RecA/RadA recombinase | 1 |
| COG0558 | I | 0 | Phosphatidylglycerophosphate synthase | 1 |
| COG0573 | P | 0 | ABC-type phosphate transport system, permease component | 1 |
| COG0575 | I | 0 | CDP-diglyceride synthetase | 1 |
| COG0580 | G | 0 | Glycerol uptake facilitator and related permeases (Major Intrinsic Protein Family) | 1 |
| COG0632 | L | 0 | Holliday junction resolvasome, DNA-binding subunit | 1 |
| COG0732 | V | 0 | Restriction endonuclease S subunits | 1 |
| COG1079 | R | 0 | Uncharacterized ABC-type transport system, permease component | 1 |
| COG1162 | R | 0 | Predicted GTPases | 1 |
| COG1481 | S | 0 | Uncharacterized protein conserved in bacteria | 1 |
| COG2255 | L | 0 | Holliday junction resolvasome, helicase subunit | 1 |
| COG3331 | R | 0 | Penicillin-binding protein-related factor A, putative recombinase | 1 |
| COG0009 | J | 0 | Putative translation factor (SUA5) | - |
| COG0130 | J | 0 | Pseudouridine synthase | - |
| COG0266 | L | 0 | Formamidopyrimidine-DNA glycosylase | - |
| COG0286 | V | 0 | Type I restriction-modification system methyltransferase subunit | - |
| COG0610 | V | 0 | Type I site-specific restriction-modification system, R (restriction) subunit and related helicases | - |
| COG0736 | I | 0 | Phosphopantetheinyl transferase (holo-ACP synthase) | - |
| COG0775 | F | 0 | Nucleoside phosphorylase | - |
| COG0816 | L | 0 | Predicted endonuclease involved in recombination (possible Holliday junction resolvase in Mycoplasmas and B. subtilis) | - |
| COG1322 | S | 0 | Uncharacterized protein conserved in bacteria | - |
| COG1451 | R | 0 | Predicted metal-dependent hydrolase | - |
| COG1970 | M | 0 | Large-conductance mechanosensitive channel | - |
| COG2131 | F | 0 | Deoxycytidylate deaminase | - |
| COG3763 | S | 0 | Uncharacterized protein conserved in bacteria | - |

Proteins dispensable in *M. genitalium* identified by Glass et al are showed in the rightmost column (dash indicates absence of the respective gene or protein in *M. genitallium*).
