# Supplementary material for: Core Proteome of the Minimal Cell: Comparative Proteomics of Three Mollicute Species
Source: PLoS One. 2011 Jul 19;6(7):e21964. doi: 10.1371/journal.pone.0021964 (PMC3139596; doi:10.1371/journal.pone.0021964)
Supplement: Table S11 — COG's essential in Mycoplasma genitalium but not found in genome core. (DOC) [file pone.0021964.s011.doc]

Table S11. COG’s essential in Mycoplasma genitalium but not found in genome core.

| **COG** | **COG Category** | **Function** |
| --- | --- | --- |
| COG1192 | D | CobQ/CobB/MinD/ParA nucleotide binding domain |
| COG0562 | M | UDP-galactopyranose mutase |
| COG0171 | H | NH(3)-dependent NAD+ synthetase, putative |
| COG0061 | G | inorganic polyphosphate/ATP-NAD kinase, probable |
| COG0245 | I | conserved hypothetical protein |
| COG3118 | O | thioredoxin |
| COG1765 | O | OsmC-like protein |
| COG0721 | J | glutamyl-tRNA(Gln) and/or aspartyl-tRNA(Asn) amidotransferase, C subunit |
| COG2190 | G | PTS system, glucose-specific IIABC component |
| COG4487 | S | conserved hypothetical protein |
| COG1713 | H | nicotinamide-nucleotide adenylyltransferase/conserved hypothetical protein |
| COG0037 | D | tRNA(Ile)-lysidine synthetase |
| COG0206 | D | cell division protein FtsZ |
| COG1196 | D | HMW2 cytadherence accessory protein |
| COG0258 | L | 5'-3' exonuclease, putative |
| COG0550 | L | DNA topoisomerase I |
| COG1658 | L | small primase-like protein |
| COG1525 | L | Staphylococcal nuclease homologue, putative |
| COG0592 | L | DNA polymerase III, beta subunit |
| COG0280 | C | phosphate acetyltransferase |
| COG0554 | C | glycerol kinase |
| COG1210 | M | UTP-glucose-1-phosphate uridylyltransferase |
| COG1087 | M | UDP-glucose 4-epimerase |
| COG0212 | H | 5-formyltetrahydrofolate cyclo-ligase, putative |
| COG0196 | H | riboflavin biosynthesis protein RibF |
| COG0431 | R | NADPH-dependent FMN reductase domain protein |
| COG0262 | H | dihydrofolate reductase |
| COG1393 | P | Spx subfamily protein |
| COG0572 | F | uridine kinase |
| COG0208 | F | ribonucleoside-diphosphate reductase, beta chain |
| COG0317 | TK | GTP pyrophosphokinase |
| COG1780 | F | nrdI protein |
| COG0459 | O | chaperonin GroEL |
| COG0234 | O | chaperonin, 10 kDa (GroES) |
| COG0690 | U | preprotein translocase, SecE subunit |
| COG0576 | O | co-chaperone GrpE |
| COG0260 | E | cytosol aminopeptidase |
| COG0682 | M | prolipoprotein diacylglyceryl transferase |
| COG0463 | M | glycosyl transferase, group 2 family protein |
| COG0463 | M | glycosyl transferase, group 2 family protein |
| COG0631 | T | protein phosphatase 2C, putative |
| COG0735 | P | expressed protein of unknown function |
| COG0781 | K | transcription termination/antitermination protein NusB |
| COG3343 | K | DNA-directed RNA polymerase, delta subunit |
| COG0064 | J | glutamyl-tRNA(Gln) and/or aspartyl-tRNA(Asn) amidotransferase, B subunit |
| COG0154 | J | glutamyl-tRNA(Gln) and/or aspartyl-tRNA(Asn) amidotransferase, A subunit |
| COG0359 | J | ribosomal protein L9 |
| COG2868 | J | conserved hypothetical protein |
| COG0101 | J | tRNA pseudouridine synthase A |
| COG0482 | J | tRNA (5-methylaminomethyl-2-thiouridylate)-methyltransferase |
| COG0531 | E | amino acid-polyamine-organocation (APC) permease family protein |
| COG1136 | V | ABC transporter, ATP-binding protein |
| COG1136 | V | ABC transporter, ATP-binding protein |
| COG0474 | P | ATPase, P-type (transporting), HAD superfamily, subfamily IC |
| COG0444 | EP | oligopeptide ABC transporter, ATP-binding protein |
| COG0534 | V | membrane protein, putative |
| COG3845 | R | ABC transporter, ATP-binding protein |
| COG0395 | G | ABC transporter, permease protein |
| COG0392 | S | membrane protein, putative |
| COG1253 | R | conserved hypothetical protein |
| COG1078 | R | HD domain protein |
| COG1418 | R | uncharacterized domain HDIG |
| COG2001 | S | mraZ protein |
| COG0515 | L | serine/threonine protein kinase, putative |
