# Supplementary material for: Core Proteome of the Minimal Cell: Comparative Proteomics of Three Mollicute Species
Source: PLoS One. 2011 Jul 19;6(7):e21964. doi: 10.1371/journal.pone.0021964 (PMC3139596; doi:10.1371/journal.pone.0021964)
Supplement: Table S13 — Primers for Acholeplasma laidlawii ORFs. (DOC) [file pone.0021964.s013.doc]

Table S13. Primers for *Acholeplasma laidlawii* ORFs.

| **Locus** | **Forward** | **Reverse** |
| --- | --- | --- |
| ACL_0021 | TAGAAAGACTTGGCATCAATCC | TAAACCCTGAGTTCAATACTGG |
| ACL_0052 | CACTTATCATTTATGCTGGTGG | TACTTCCAGTACCTAAGAACTCTC |
| ACL_0053 | TTGGATTAGAAGCAATTAAAGATCG | TCAAATGCAACATTTGACAACC |
| ACL_0056 | GCAGTAGCAATTGTCTTAAACG | GTATGTGTAATAGCACCTAAGACAG |
| ACL_0120 | GTAGTAGCATCATCGGTTATGG | CATGATCATAGACTTGATACAGCAG |
| ACL_0122 | TTATGAAACATGTACATGAAACTTCAG | ACCAACTTTCTATATCTTGATAACTGTG |
| ACL_0131 | CCCAATATTACTTACAGAAGATGCC | CAGTTAAAGTGATACCATCATGCC |
| ACL_0135 | CCATTTACACCAAACAACTTGC | GGAAGTAGTAAAGGCTGAAGGT |
| ACL_0144 | GGGTTAGCACTTGCATATTTAGG | GTTGCTTGTTGATATGTCACCA |
| ACL_0148 | AATGTTGTATTACTCGCTATTATTGG | GCAAATACTAACAAAGATCCATAGAG |
| ACL_0161 | GGTGAAGATGAAAGAACTTTCC | CCATGAGTACTTTACTATGTACACG |
| ACL_0164 | ATCACTTCGCAATACTACTTCC | GCTACATGTAAATGGATATGTCCC |
| ACL_0165 | TCATGGCATATTCTTTAAGCTAGG | CAATACCGTAATCATGGATAAGTG |
| ACL_0173 | ATGATGTTCAAACAAGAACCCG | TAAATACACATCACTATACTGCATATCC |
| ACL_0175 | CGCAAGTCTATATTAAGAGTGC | TCTCTTATAACTCTTATCGGCC |
| ACL_0179 | CGCAAGATTTATCAAAGGATGG | CACCTTATTTCCTGTAGTACCC |
| ACL_0199 | CCTTAGGTGTTGTATTACCATCC | TTTCCTCGATTCATGAATAGTCC |
| ACL_0200 | AAATCGGTTTATTTACATTTGGTGG | TCCAACTTAATGCATATCTAACAGG |
| ACL_0207 | GTCAGAATTCATAGCTATTATTACACCC | AAACTTGTGAGATAGATTTAGCATACG |
| ACL_0212 | ATTAGTGTTGGTAAAGTTAAAGATAAGG | TTTCTTTAACAGCATGACTTAAACC |
| ACL_0213 | GTGGAGTTTAGAGACTTACAAGC | ATTAGCAATTGAATGTCTTCTGG |
| ACL_0231 | TTAACACTCGAAGATAGTTCCG | AGTTCTTGGTGGTTTAGTTTGG |
| ACL_0234 | AAGTGAACTTGAATCAACTGGG | GAACATCTACAGTGACTTCATTTCC |
| ACL_0239 | TTTGTGGCAAAGTTTGAATCTG | TTTCTATAAGCATGAGATCTAGTGG |
| ACL_0259 | GCAATCTTAGTAAGTGAGCTAGAG | GCTTCTACACTGGAAATAATGG |
| ACL_0260 | CATTTACACTGGTACTTGTAGC | CACTTACAACATATTCATCGCC |
| ACL_0299 | TTTACTTAAATTTGAATTACCTGGTG | CATATCTCATAACCTTTATCCTCC |
| ACL_0307 | TAATCATAAGGTAAGTGTTTATCCAG | TCATCTAGATCAAGAAACTTAATGG |
| ACL_0333 | TAATACGTATGATGCCATTATGACC | ACATCACACTATCTTCATTAAAGCC |
| ACL_0340 | CAGTAGAACTTGTTAAAGCAGG | CCTAGTACCTTATACTCTTTGCC |
| ACL_0348 | AGTGAACTATTAACGGATAAACAAC | TATTCATCCAACTTTCTTAACTCATC |
| ACL_0407 | TGAAAGTGTCGATCTTAAACGC | GGGTTAAACCCGTAATCTAATGC |
| ACL_0409 | TTTCATAAATCGTTTAAAGAGTTAACC | ATTCTAATATCTTGACTACTTACAGC |
| ACL_0411 | CCGTCTAGATCTTACTGATTTATGG | GGATACATCTTATGTTCCTTAGCC |
| ACL_0412 | TACGCTAGGTGCTAAGTTAGAC | CTGATGAGTTGGCTTCAAAGAG |
| ACL_0415 | GGATAGAGGCATTATTAAGTGG | CATCTAAGGCTAATCTAATGCC |
| ACL_0427 | AAATGTATGTACCAATTGTTGATCGT | CGCACTTTCATCATATGCCTTA |
| ACL_0438 | AAGAGATTGATTCTACTAATGCTGG | TGTATCATAATAATCCGCTCTATTCTC |
| ACL_0439 | TGAGAATCAAGGAATTTGGTGG | GGGTTAACCATTCAATCCACTG |
| ACL_0441 | CATGTTTCATCTTACAGTGAGC | TTCTCGATACGCCAAATATGAG |
| ACL_0446 | ATTCTATTAATGCATATACGAATTATGG | CTCCAATGTTATCTTTATGTACTCC |
| ACL_0511 | GAGCAAATACTCACATTTATGCC | ACTTCTAAGATAAACACAAACGG |
| ACL_0515 | TATGATTGAGCTACATACGTGG | TTCAGCATCGTAGTTTAATCCG |
| ACL_0521 | CTCCCAGTTGTTATACTATTTCTAGC | AAATGTTAGTGTTAAGAGTAAACCG |
| ACL_0525 | GATAGTCCCAACTTTCAACTACC | GTCCATTATCGGTCATAAATCTATAAGG |
| ACL_0570 | AAAGAGAAAGAATAGATTATGGAGTG | CTAGTATTCAAGTTCCATTTCTAAGTG |
| ACL_0576 | TACATCATATAGAACACTAGAACTGG | AGAAACAGATATGAATGAAACAAGG |
| ACL_0577 | CTGCATATTCAGTGTTTCTATCG | ATATGATCACTTTCTTCTATACTTGG |
| ACL_0578 | ATATGTATTAAATCAAAGCAAGTTACC | TACGCAACATAATGATATAAATATGAC |
| ACL_0579 | AATATTTGAGTGGTTTGAATGGC | CAACAATGCATCCAAATCAAATCC |
| ACL_0582 | ACATATAGATGACAAGATAGTGCC | TTTCAATGACTAAATCTTTCTTAAGTG |
| ACL_0583 | ATGTATGAAGAGGATTCTATGCAC | TTGTATTTCTTGATGCCGTTCAG |
| ACL_0584 | CAGTGACATTATGGCTTATCGG | CTACTTCAACAGTTCGCATACC |
| ACL_0587 | AATAACAGTAGTTCAACCGCAG | GCTGCTAAGACATCATCAAATCC |
| ACL_0588 | ACAAAGTTAAACTCATAGGATATCATGG | AACCACAATCTAATGACTTCTTCC |
| ACL_0593 | GAACAAAGCATTTCAATATGGG | CTTCATCACGATATTCATTGATCC |
| ACL_0596 | ATGAAAGTCAAATTTGAAACGAGAG | CATCACCTAATCCTCCTATCTCAC |
| ACL_0597 | TTGATTCAAGCGTTCAATAGTGG | GACTGTGTAAGTCTTGTCATGC |
| ACL_0598 | TCTCAACAGCATTTGATAAAGAC | AATTCTCCATGCTGTATAAACAC |
| ACL_0599 | AAACCAACACCAGAGGAACTTA | TTTAATAAGTGGTATGAATGCTGCATA |
| ACL_0602 | TTTCGGATCTAATTAGAAAGAAGAGTG | ACAGTCTTACCTATTAATAATGTATCGG |
| ACL_0604 | TATGCAAATGCCTAAGTTAGCC | AAACAGTCATATCATCGTCATCC |
| ACL_0605 | CCAAATATGACGAGACTTTGCC | TTGTTCATATTGAGTTATCTTCTCTTGG |
| ACL_0606 | TTCAGATCATCAAGATACATAATTTCC | TTATGCATGATTTCCAACTCAG |
| ACL_0607 | GAATATTTGCGACTTAAGTCGCT | ATTCAGACATTCTACCTATAAACTTGTC |
| ACL_0608 | GACTTGTCATCAACAACAGACC | CCATTTCAAACCCTAAGTCTTCC |
| ACL_0609 | AATGAACTTTATAGTAATAACAAAGCGG | CATTCATCGCTGATTCACTAAGG |
| ACL_0611 | TTAAAGATGTACCAATGAAGTATAACC | ATAAATCGAAGTCTTATCATAAGCAG |
| ACL_0612 | CAGGTACTTCTAATACATTCACAGG | TCCAGCTGAAGTATTTGTATCC |
| ACL_0614 | TCCAAATATTCGACTGACTTTACTG | CAACGTAGCCATAAATATCACTTACC |
| ACL_0615 | CTTGAACAGAAACTTGATGAAACTG | TTAGTTCCCACTTATAATCCTCC |
| ACL_0616 | GGACTCCTAAATCAAGTGTTACC | TCCTCCATCTTTATTTCATAAACTCG |
| ACL_0617 | GGGTTTGCAAGAGATTCTAACG | ACACCTATAACAGGTAGTGTTGG |
| ACL_0618 | AGAGGTGAGGATCTTAAACTAAGG | GTCTTCTTTACCTTCATTACCACC |
| ACL_0620 | GTCGTTTCACTAAGTAAAGCAG | GTTTAGTCGTAGGCTTGATACC |
| ACL_0622 | GCATCAACTTATCCAAATCTCG | CTGGTATGTTCACAATTGTTCC |
| ACL_0623 | ATGCAAAGATTACAGCACTTGG | CTTTGTCGTATCGAATTTAGCTCC |
| ACL_0624 | ATGAAGGAACGGGATTTATACC | ATGCTTAACATACCCAACTGTC |
| ACL_0625 | CTGCAAATACTGGAAACATTGG | GAGCTTGCTGTTACTTTACTTCC |
| ACL_0626 | AACTTACAATCAAGGGTTGGTG | CCCTCAACATCTAAATCATCTATCG |
| ACL_0627 | GCGGTTCAGATAACTACAAGAC | ATGACTGCATCCAAGTATTGAG |
| ACL_0635 | CTCAAATAGTTTCAAAGATTCTGG | ATCATCTATTCCATTATTCTTTCCTG |
| ACL_0648 | GGTCTAGCACAAATTATTGCAG | ATAGCAGTGAAATCACCTAGTG |
| ACL_0649 | TTCAACTGTCTATTACATGGCC | CCATCTTCTGGATTACCTAGTG |
| ACL_0675 | GGATTACAGCTTACAATACATATCAG | GCAGTTGTATTATAACTTGATGGG |
| ACL_0688 | TGATGTACACGATTTGGATGTC | CTTTATGATAGTAACACCGCTG |
| ACL_0695 | GATTTAAAGCTGATGATTGATGG | CATAAGTGATTTCGTCTAATAGGG |
| ACL_0696 | AGAACTTATCTATGCACCAACG | CTCCTGTAAAGTTCGTTGTCTC |
| ACL_0697 | AAACTACACTGAAACCTTACCG | TGAGTTGATCACTTACTGGACC |
| ACL_0713 | GGGACATTACTAGATACTTTAGATGAC | TCTTGATGTTTATTGGATACGACTG |
| ACL_0716 | TATCTGTCTGATGCATTTGTGG | CATGTTGATGTACACTATGAGC |
| ACL_0726 | TTATTATGGCATCTCCATGGAG | GATCCCAATTATGGTCATATGC |
| ACL_0728 | TGTTGCCATACTAAATTATATTGATGAC | TATAGTATTTCGTAGAATTCAGATCTCC |
| ACL_0750 | TTTCTCTGGATTTATGTTGGGT | TGCCAATGATTGGTATTAAACC |
| ACL_0754 | ACAAGATATCGTAGACTCAATCAGG | ACTTGAGGGATAATTGGTTTCG |
| ACL_0762 | GATCACTTTAGTTGTCACATCAC | CTATTAATGTCTATGATGAACCAGAC |
| ACL_0769 | ATTGGTTTGTATATCCAGTCTTAGC | ACATATGAGAATTTAAATCAGCAACC |
| ACL_0771 | GGAACGTGTTATTGACTTAACG | TTTATGAGTGCGTCTTTAGCAG |
| ACL_0785 | GGATTAAGCATGTCATGTTACCG | GTCTTAACCTTTCACTTACAACTCG |
| ACL_0833 | TACTACAGAACACCTTCTACTACC | TTCATAACTAAATGGTATTTGGTGAC |
| ACL_0847 | CATACACCTTAAAGGTCAGTGG | TCGGTGTAATTTCTAATCGCTG |
| ACL_0851 | AAAGATAATCCAAGACCATATGTACG | TTTACGTTCATTTCTTATTGTTGAAGC |
| ACL_0859 | CATGAAACTGGACAGTTTACAG | TCCATTCAGTTCCAGTATTAGC |
| ACL_0863 | AAACCAACTGTAGATGCTCT | CGCCAAGTGTTTGATCTTTC |
| ACL_0865 | CCAACAAATAGAAACTATGAAGATGG | ATTCTAAATCCTGTAGATTATAAACACC |
| ACL_0891 | TTTACAGCACTGCTTTATTTAATGG | ATAAATAATCAAATCCAGACCAAACC |
| ACL_0902 | AAACAAGTACTAAGTATATTAGACAGTC | TTCTTACAATTTGGCATGAACC |
| ACL_0903 | ACAACATCACATCTATATTAGAAATTGG | TTATCACAGATAATACTACCGCC |
| ACL_0909 | GATTACGATGTGAATATTGAGATGG | AATCTTCATTTCTTGAACCATTTAGTC |
| ACL_0913 | AAATAGAACATATAAAGCTACCACG | ATACAGAAGTTAAGTGGATTATACC |
| ACL_0917 | CTTATGATAACATCTTATATGGCTGG | GGTGCAAAGTTAATAGATTGCG |
| ACL_0922 | AGATCATAGTTTATGGGAGACG | ACTTCTTCAGATGCAACTATACG |
| ACL_0924 | TAACTATCATGAAGCTGAAATATTTGC | AGATCACCTGATTTAGTATAGTATTCC |
| ACL_0926 | AGATACTACTTTCCTATTAGATGTACC | ATCTATTCGCTTCAATTTCTAGAC |
| ACL_0927 | AAATACAGAAGAAATGTTTGTGAAA | TTAAATAGTAGTATGTATGTTCCACC |
| ACL_0928 | GCAAATAAAGACACATATCAACTTTGG | AGTTCTTACAATCTTTCTTCTTGGTG |
| ACL_0929 | AAACGGTCCTTAATAGTCGTCA | ATGGCTTTAACAGTTTACTCACAT |
| ACL_0934 | GAGCCGAATAAACTTGATAATACTG | ACATCCATGTACAATTATACTTAACTG |
| ACL_0948 | ACAGATGCATTAATGGATGCAG | AATCATCTGGATAGTAATGCGG |
| ACL_0950 | TTTAACCAATATAAAGAAGAAACAACAC | ATACCCAAGTAGATGTAGATAACC |
| ACL_0953 | ACTGTTACAAAGTATGCGACAC | CAACAATGACAATTAAGTCTTTATTACC |
| ACL_0959 | GTAGTTGTACGTACCATGAAGG | AGCTACTTCAAGTATCTCTTCTGG |
| ACL_0974 | CAGAAATAGTTGCATGTAGTACG | TTTCAACAAGTAATTTCAATTCGG |
| ACL_1002 | TTAGTAAGTATCATACGTTCCATTCC | GAAGAATAAGCAAGTAGACTTACC |
| ACL_1005 | CTAGGTGCTTATGGATATCAACG | TTTCTTCTAATGACCACCAACC |
| ACL_1006 | TTTAGGAGATCGCATAAGAGAC | GTTGATTCATCGCTGTAAACAC |
| ACL_1007 | GTACTTACACCATCTATGGAACC | TGATACCAATGACTAAGAGTCG |
| ACL_1012 | CCTGTATATGATGAAGCATCTACTC | CATAGATTTCATTCGTTGGTGC |
| ACL_1024 | TTATTTATGAAGATATTGTGGAGATTGG | GTACTTTAAGATCAATACCGCC |
| ACL_1030 | GGATACTGGTGTGAATAGATTTGC | GCATTTGACCAAACTTCTTAGG |
| ACL_1031 | AACACTAGATTCGATTAGAGCG | GTTCTTTACCCATTACACCTGG |
| ACL_1049 | TGGTAAGTTCTTAAGATCAACATCC | TTTCTTCTAATCACCTTAAATGCG |
| ACL_1056 | CAGTCTAGTTAGAACATTCAATCC | TATCATCATAGTTTCTAGTAAATGTTGG |
| ACL_1057 | AAAGGTGTTGCTTATCATGTGG | GATCTTTATCAGTGAATTTCTCGAC |
| ACL_1058 | GCCTTAAGAAAGTAGCTAAAGG | CTACTTCTTGGTCTTTCATTCC |
| ACL_1060 | TCTTTATTTGCACTACAAATGCC | TCAGCACACCATTATGATTATCTG |
| ACL_1070 | ATGAATCAGTCATAAAGGCAGC | AGATAATGATGCCGTCATTTGG |
| ACL_1073 | AATCAATTTCAATACAAATAAATTGGC | ATCAACTATATCGAGTATCAATATCTC |
| ACL_1080 | GGTTGGAACTATCTTGTTTATACGG | TTCTACCTATCTTTACCCAACCAG |
| ACL_1082 | GAACACCCGAATATCATAACTG | CTTCAAGTATGTCTATGCTTGG |
| ACL_1115 | ATGCAGAAACCTATGTAGAACG | CGTCATGAGGTATCTGTTTAATGC |
| ACL_1121 | ATTATATCATTTGTTGATTCATTGATGC | TAGATTGCCTATCTTAATTGATATGGTC |
| ACL_1175 | ATTAATTCGTGAGGTTATTCAACTCG | CCAAACATCTTTATTAGCTTCATATTGC |
| ACL_1196 | ATTCCTAGATTATAAACAAGCCG | AGTTTCATCAAATACTGGAATGG |
| ACL_1210 | CAGATGGTGGCATCATTTATCC | TTTAGTTGATAAACTGACCTTTATTCG |
| ACL_1211 | CATATTGATCAACTACAGACAGTCG | CACATGATGTTTAGCAAGTGTG |
| ACL_1229 | GTTATCAGAGCTTCAAGATCTTGG | CAAGTTCAGTATCACTTGGTAAGG |
| ACL_1240 | GGGATAAGAAATTTATGAATGCAC | AATATTATAAGTTCCTAATTCCAAATGG |
| ACL_1242 | CAGATAAAGTCTATGTTGCCTTACC | CAGATAGGTAAAGATACTTAGCGAC |
| ACL_1246 | TGATAAGGTTCGTGCTTATGAG | TTAATGCCCATACCATAACCAC |
| ACL_1256 | AGATATGGTCAAGATGCACTCC | CTATCCATAAGCATTTGTGCTGG |
| ACL_1257 | ACGAAGTGGAAAGAATCTACTC | GTCTTTGTTCAATAGAGTCTGG |
| ACL_1261 | AAGTAAATATAACAACGGTGTTAAAGAC | CAACGATTGCTTGAAGATAATTCC |
| ACL_1263 | TGAGACCTTATACGGATAGAGG | TGTCAATGAATAGTAGTCGATTAATCTC |
| ACL_1265 | TTATAGTCATGTTGATCATAATTTCGAG | TCTTTAGAGTTTCTTCTATTGAATCC |
| ACL_1266 | AGTACACCTACATCTCTTGAACTG | CTGAAGTGGCAATGAATCTAATCTC |
| ACL_1273 | GACTTCCAAGGAGTATTAACTAGAC | TCTAAAGCATCGAAATAGCGTG |
| ACL_1287 | AAATCAATTAAAGATGACTCAAACC | ATCCTTCATATTAAACTCAATAATTCTC |
| ACL_1322 | ATGTAGTTTGGGAAACTTATAATAATGC | AGCATCTACAGTAAGCTTAACAG |
| ACL_1328 | TTAAATGGGCTGGTAACTTAGG | CTCTAATTCAAGTGGTTTACCGTC |
| ACL_1335 | ACTTGAGCAGTCATATTTCACG | AGAGTAGCATCAAATAATCCGC |
| ACL_1342 | CGGCATCTTAGGTGATAAAGAC | CAAATACAGTTTGAGATGCACC |
| ACL_1355 | AAGTATTTGGTGCTTCTTTAGG | TAACAGTCACTATTACAGCACC |
| ACL_1359 | TACTGGAATACAACCCTATGGTG | CCTCTTTCTTTGCTTTAACCCA |
| ACL_1398 | GATGAAGATACAGTGATTCGTGG | AAATAAGGTATTGCAGCAACCG |
| dnaE | CTTCTACAATTGAAGGTATACCTCG | TTGAAAGACACCTTCTACATCG |
| dnaK | CAGGTGATAACAAACTTGGTGG | CTAAAGCACGTCTAACAGATACC |
| eno | GCTAGAGAAGTACTTGATAGCC | TAGCGTTTGCACCTAATTTACC |
| gap | CGTTTAATGGTAGATAACCCAG | AACTACAACTTTCTTAGCACCC |
| gyrA | CTATCTAAGATAGCTGGTGAAATGG | GACCTGTAATACCAAGAATTTGACC |
| gyrB | GGAAGTACAGATAAATCAGGAACG | AAACTGCATAGCAAGTTCTACC |
| polC | GTTCTAGCTCAAACTCAAGAAGG | CTGCTTGAATGACTTCTCTACC |
| rpoB | CAATTACCAACTGGTGTAAACG | CTAACTCATCGTTAGTCACACC |
