# Supplementary material for: Core Proteome of the Minimal Cell: Comparative Proteomics of Three Mollicute Species
Source: PLoS One. 2011 Jul 19;6(7):e21964. doi: 10.1371/journal.pone.0021964 (PMC3139596; doi:10.1371/journal.pone.0021964)
Supplement: Table S14 — Primers for Mycoplasma gallisepticum ORFs. (DOC) [file pone.0021964.s014.doc]

Table S14. Primers for *Mycoplasma gallisepticum* ORFs.

| **Locus** | **Forward** | **Reverse** |
| --- | --- | --- |
| MGA_0016 | ATCATCAACCTTGAAGGGATTGCTTT | TTGCGCTGGCTAACCCCTTTA |
| MGA_0018 | TGAAGATCACATTAATGAAGCACAGCA | TCACTGCTACTGAGTGAGGCAC |
| MGA_0019 | AACCCAGTAATGACAACAATTCCTGAT | AGAAAATGCTGAAATCATAACCCCACT |
| MGA_0021 | AGCGATCATCTTTCTTGTGAGAATCAAC | GCTAATCGTTCCCACATTCTGATCG |
| MGA_0022 | TTGCGGCATTGCTGTAATCAGAA | TGCCAGCAAAGCGTTCTTCAAA |
| MGA_0028 | AGTGGCTGATCAAAACTATTATGCAGT | TCGTGATCAGATCTTCGTTACGGT |
| MGA_0046 | TGGTGCAAATTTTGTTTTGGCGTT | ACCAAGCCCTTTTAGTGAATCGGT |
| MGA_0049 | ATCAAAGCCTTTCTAGCAACGAACA | CACTTGGATAACCAAAGCTGCTAAAGT |
| MGA_0052 | TGAGATGAAGCTGAGATGATGGCA | CTAGCCAGCTGACCAACCCT |
| MGA_0054 | CAGTGGCTATGCACATTGCTGT | ACGCCAGCAACACTTGAATCTC |
| MGA_0082 | TGGTGCCACAAAGATTAGTCCCAA | AGGTTACCCAAATCACAAACGATAATCA |
| MGA_0099 | CAACCTTGAAGCTGTTGATTGA | CCTAGCCAAATAATCGACGAGA |
| MGA_0103 | TGCGTACGAGTGCTTCATTG | GCAATTGGTCTAGAACTATCTGGAAA |
| MGA_0107 | TCGCACAAGGCAATGAATTT | AAACTTACTTGGTGAGATCGGTGT |
| MGA_0111 | GAATTGGTTGTTGGGTGAAGG | ACGATCCGGCCAAAGATG |
| MGA_0115 | GAACACCAAACCAAGCAGGT | TCCCAAGCCTACTAATGGCATA |
| MGA_0123 | CCACGACTTGTTGGTGCAA | CTAAAGAGGCTGAAGGGTGGTT |
| MGA_0132 | TGCGGTGTGAGTGTCTCTAGC | CAAATGCGATTGCTCAACCT |
| Enolase | GGTGGAGCTCACGCTGATAA | GCTGCACAGTCAAGTGCAAA |
| MGA_0217 | TGTTTGATTATTGTGGTTATTGCTTT | GTCGCATTGTTGTGGTCGTA |
| MGA_0221 | CAATACAGTGCGACCCAACC | GCCATCGCTTGTCACAGATT |
| MGA_0247 | GGAATTGCTACTGAACCTGGATT | ATTGTGAGTGGAGCGCCTAA |
| dnaK | TGGAGCTTCGCCACCACCGT | GCTGAAGCTAACAAAGAACGTGACGCA |
| MGA_0280 | TTTAACTGAAGATAAGCGTTTGGAA | CACCACCAGGGATTGGAATA |
| MGA_0281 | TGTTCTTTCATCTGGTCAAGCA | GGCAGCTCGCTTACGAAGTA |
| MGA_0284 | CGCTAAGCAAGAATGCGATG | TGCCTTACGGACTTCGATTATG |
| MGA_0287 | CGCTTTATGCGGTATTCCAA | TCCTTCCCACACCTGTTCC |
| MGA_0289 | TGGTATTCTAAGACTAGTAGCCACGAA | AATCACCGCACACCAAAGAT |
| MGA_0298 | CGTTTGGGATCTTAGCGTTG | TAGCCGCATTGATTTGCTCT |
| MGA_0310 | TCAGATCCCGCAAGAAACAT | GCGATAGCTTCGAACCAGAA |
| MGA_0312 | GTTGAACGGGTTAAGCGTGA | AAGCACGGCTTTGCATATTG |
| MGA_0313 | CTTGCAATTTGGCAGACTAGGT | GATCGCTGCTCAAGCAATCT |
| MGA_0315 | TAGCTGTGATCGTTGCACCA | AAGAACACCACGGGATAGGC |
| MGA_0323 | TTTGATCGCGCAAGCACAACAGATACA | CGGGATCGCTACCACTGGACCAA |
| MGA_0329 | ACATGACAAATACCGGCAGCTAATGACGTA | TGATTCAACGTTGTCAAAGGTTTGTGCTGT |
| MGA_0337 | TCTTGGATATGGAACAGACTTCAA | GACTGCTTGTTTGGGTTGGT |
| MGA_0344 | ATCTGCAAGCGATTTGGCTA | CCTTTTCCATGAGCGTGATT |
| MGA_0346 | CAGCTTCATTGCTTGCTTCA | CAAGAATGAGTAAAATCACCAAACC |
| MGA_0361 | TTTAGCAGTCATTGCTTTGTGATT | ATTCGAACTTTCATGTTGCTTGT |
| MGA_0365 | AGCCCCTGCACTGATCCTTGGTG | CGACCAAATAAAGTCGTAGCGCGGTC |
| MGA_0436 | CGGTGCTTCAACTGGAGGGATGGA | AGAAAGCAGAGACCACTATCGCTGAAACG |
| MGA_0474 | TCGCACTAACAAATTGGGTAAGA | GATCCCGATCGCACTAAAGA |
| MGA_0477 | TCATCAGGGTTCTAGCTGAAGG | TGATGATGATTCCAGCTGATTG |
| MGA_0482 | CAGAAACCCAGAATGCTGCT | TTCGAAATGCTTGATTGGTAAGA |
| MGA_0484 | TGATCCAGTTGCTGATGAAGAA | TCTGCTAGATTGAAGTTGCGAAT |
| MGA_0485 | CACCAAATGCAGATCCACAA | TGTTCAGCCGGATCTTTGTT |
| MGA_0487 | TGCCACCGTACAGATCCAAGACTCAG | TCTGGTTTGTTGATTGCTCGCATTGCT |
| MGA_0507 | CAAACGACCAACCGCACTAC | AAGATGACTCTTGATGCAAACCA |
| MGA_0508 | TGGCTGCTGCCATTCCCATTGC | CGATTGGTCCACCGTGTGCTGC |
| MGA_0514 | TTTGAATACCGTGTCTGATCTGG | TGGCATAAGCATCATCAGGA |
| MGA_0518 | GCTGCAACCACTGTGCCTAT | TGGTTGATTCCGGATTAGTTTG |
| MGA_0523 | GCCGCATCCGCCAGAGCATT | CGAGCTAATGGGTAAGATTTGCATCGTTGG |
| MGA_0549 | TGAGCTACTTATCCATCAATTCAAGA | CTTGCAAATGCCATTATTACTAAACTC |
| MGA_0552 | CAGTTTAGGTAACGGTCGTG | AAGGCAAGCGAGTTAATGTA |
| MGA_0553 | TCTGCTGGTTTAATTTCAGG | AGCAGCTTGCTCCTTAGTTA |
| MGA_0558 | GAGAAACAAAGAAAGGCAAAGA | TTGATTTCGAACCATCAGATTT |
| MGA_0562 | ACAAGATGCAACAAGAGCAG | GATCCAGGCATATCACGATT |
| MGA_0564 | TTTCAAGGTCCATTTGGAGA | TAAATCAACCGCGTTACCTT |
| MGA_0567 | ATACGACCCAGAACTTGCTT | CGAATCGCTTCTTCATTTGT |
| MGA_0576 | TCGTTCCCTTTGTAGCCTAT | ACACCAAAGCCTAAACCAAC |
| MGA_0578 | TGGAACCTAAACCTGAACCAAGACCCGT | GAGCAGCTGTGTCATCAGCACCGT |
| MGA_0583 | TTCAAGGAGCATTTGGAGAT | CCATAAAGTTCTGGACCTCAA |
| MGA_0584 | ATGCTTCAAATGCAAACGAT | TGTTAATTTGCCACCAGGAT |
| MGA_0588 | ACGGGAATGGTAAAGCACAAGACGCA | GGATCGTTAGGTTGCTGAGGTGTAGTTGC |
| MGA_0626 | TCATTCCAAGCATTAGTCCA | TTTCTTCATAATCGCAGCTT |
| MGA_0631 | ACCCTAGAATTGGTGTTGGT | TTCAGCAATCTTACCTTGGA |
| MGA_0635 | TCACCTACCCAACTGTTTAGC | CAGCATTACTTGGAGCTGAG |
| MGA_0650 | GCGTCATAATGAAGTGGCTA | AACTCTCCCAACTCGATGAC |
| MGA_0656 | TAGCAACCAAGGGACAACTA | TATATAAAGGCGCAGGTGTT |
| MGA_0657 | GGCTTTGATAGCCACAAACT | GGGCAAAGATTGTTAGATCG |
| MGA_0680 | TTCCTTGGTTGACTTCAGGT | TAATTGCTGCATCTGGGTTA |
| MGA_0689 | CCCGTGAAGCTTCTTACGCTTTGGG | TGTTCTGCGCGTTTGCTTGGGT |
| MGA_0704 | TAATCAGATCTGCAGCACCA | TTAAGATTCCGTTTGCATTG |
| MGA_0754 | GGAGTTTACTCACCCGATCA | TTATCGGAACAATTTCGTGA |
| MGA_0760 | AGCTGCACTTCACAACTTCTACACGACA | TGGGCTATTGGCCCGTGGTCAT |
| MGA_0765 | GATCATGGATGGGTACAACC | GTTGATTTGGCCTGAACTTT |
| MGA_0789 | GGCGTTTGTGATGCTTGAAAGTACCAAGT | TCCCAGCTCCACCACCAGGGA |
| MGA_0797 | CCTGGTGGAAATAACTTCGT | CAGTTTGAATGCTGCGATTA |
| MGA_0798 | CGCGAAATGATTTATGTGTT | AAGCTGCCTTGGCTATTATT |
| MGA_0800 | AATTCCCTTTGTTGGATCAG | TCCTTGAATTGTTGCTAACG |
| MGA_0805 | GTTGTTTGCTTCAATCATCG | AAGGTTGGTTCGTCATAACC |
| MGA_0816 | AATCCTAGCATCAAATCAAACA | GCGGTAATGCTGTTCTGTAA |
| MGA_0817 | TTTGCTGTTGCTGGTTTAAT | AAATACGGAAGGACTGATGC |
| MGA_0830 | GGCATTATGCAAAGATTTGTT | CCAGCAATTAACAAAGCACA |
| MGA_0831 | CCCACACTGATAGTTATGATCGTCTTGGCA | TGGCTGTGGTGTTGAATTGACTGGC |
| MGA_0832 | GGTGGGGTTGATTCTGCAGTTTGTGC | CACGTCTGGATTTGGGGTCAGACCTT |
| MGA_0837 | ATTCCGATTATGCGTGATGA | TTTAGCTTTAGGCCCATCAA |
| MGA_0844 | TGCGATTATTTGTCCTCCAGATCGGGTT | TCGCGTGATGAATCGGTGCTCCA |
| MGA_0847 | AAGCGGGAACGTCTATAAGG | ATCATCCATCGGAGCAAAG |
| MGA_0865 | GGAAAGTTATCGCATTCCAA | TTGGCTCAATTCTCCTTCTCT |
| MGA_0867 | TGAAGGTGGTAGACACCAAAGAAGAGTGGA | ACCTACTGTGCCTGGTTGAATGGTTGT |
| MGA_0907 | TTCTACAGCTTCTGCGAATGA | AAACGAAATGCCGATCATAA |
| MGA_0908 | TTGCAATCAGTGGTTTGGTT | AAAGGAACTGTTGCTGCGTA |
| MGA_0956 | AATCCCACCTGTATGCTGGGATCGCT | ACCCCGCTTAAACCAAAGGTAAGAAAGACT |
| MGA_0994 | AGAAAAGCCTATGATGGTGATGGCGGA | ACGCTTGCAATTCTATCTCCAGCACTGA |
| MGA_0997 | GGGTTAGCAAACGTGATTGA | CAGCCCAATCGTTTCATTTA |
| rpoB | CGAATTTGTGACGACCTGCT | TCGAATCAGACCGCATTAGC |
| MGA_1010 | ACTGTTGCAGCTGGTACGGGGA | CGTTGGATCGAATGACTGGCAACAAAAAGA |
| MGA_1011 | ACCGCCAACCAGGGATTCCGTT | TCCCTTTAACACGCGCCCTACGA |
| MGA_1019 | AATATACCGCCACAGGAACC | AAATTGTTCGACTTTGCTGTTT |
| MGA_1027 | AACCTGAGAGGAGTATTTCTATGAACA | TGCTTGACTATAATTGAACTCGTTG |
| MGA_1029 | CGCGATCGAGTTGGCAATCGTGA | AGCGCGAGTTTGATATCAGTGTTAGGTGG |
| MGA_1031 | AACGATCTATTACCAAGCCTGA | TTGATGCTGATTGAGCGACT |
| MGA_1161 | CTCACTGGGATGGCAGTTGT | TCAACGGGTTGAGCTTGTCA |
| MGA_1164 | ACGTGCTGGCTGGGTCGATCT | TCGTTGGTTCTCGATCATTTGTTGACCCT |
| GAPDH | AGGACGTGCTGCTGCATTTA | AAGATCGAACCGTGGGTTTC |
| MGA_1188 | GGTGTGGCTAATGCGTTTATGATCGTGAC | CAGCAATCCGGTCATTACCCATCCCA |
| MGA_1260 | TCAAAGGTTTTATTATCAGC | TAACGGCTTATTTATTTAGG |
| MGA_1263 | TCTAGTAGTCATAACGCGCCG | GGCGCATGATGAAATCAAAGTTAAG |
| MGA_1265 | TTTCAGATGTTGGGTGATGCTGGGAAGA | GATTGCAGCCAATGAACCAGCATGGA |
| MGA_1267 | AACACCCCGCATGCTAAGAT | TGCATCAAAGCAATCAACATCGT |
| MGA_1268 | TCCCGCGATGACTTATGCAACTGGA | CCACATCAAAGATCCCTGGTGAGATGAACC |
| MGA_1271 | AGATTTATTGGGATCGCTCATCAGA | GCACTCGCAGTTTTGCGATA |
| MGA_1315 | ACGAGATCACCAACACCAAAGT | TCGTTCGTTCAACCAATTGTAGC |
| MGA_1321d | CTGGAAATGAAGTTGCCGCT | TGCCTTTGACCGATAAATTCAGG |
| MGA_1322d | GCTTGATCACTGTATTAGTGCCC | ATTGCCGTTCGACCAGATTG |
| MGA_1325 | TGAGGGAAAGAAGAAACTCCCA | TTTCAAGCGACCTTTCACGC |
